# Gun violence incidence during the COVID-19 pandemic is higher than before the pandemic in the United States

**DOI:** 10.1038/s41598-021-98813-z

**Published:** 2021-10-21

**Authors:** Paddy Ssentongo, Claudio Fronterre, Anna E. Ssentongo, Shailesh Advani, Emily S. Heilbrunn, Joshua P. Hazelton, John S. Oh, Jennifer S. McCall-Hosenfeld, Vernon M. Chinchilli

**Affiliations:** 1 Department of Public Health Sciences, Penn State College of Medicine and Milton S. Hershey Medical Center, 90 Hope Drive, Suite 2200, Hershey, PA 17033 USA; 2 Center for Neural Engineering, Department of Engineering, Science and Mechanics, The Pennsylvania State University, State College, PA USA; 3 Centre for Health Informatics, Computing and Statistics, Lancaster University, Lancaster, UK; 4 Department of Surgery, Division of Trauma Surgery, Penn State College of Medicine and Milton S. Hershey Medical Center, Hershey, PA USA; 5 Department of Oncology, Georgetown University School of Medicine, Georgetown University, Washington, DC USA; 6 Social Behavioral Research Branch, National Human Genome Research Institute, National Institutes of Health, Bethesda, MD USA; 7 Department of Medicine, Penn State College of Medicine and Milton S. Hershey Medical Center, Hershey, PA USA

**Keywords:** Epidemiology, Viral infection

## Abstract

During the coronavirus disease 2019 (COVID-19) pandemic, gun violence (GV) in the United States (U.S.) was postulated to increase strain on already taxed healthcare resources, such as blood products, intensive care beds, personal protective equipment, and even hospital staff. This report aims to estimate the relative risk of GV in the U.S. during the pandemic compared to before the pandemic. Daily police reports corresponding to gun-related injuries and deaths in the 50 states and the District of Columbia from February 1st, 2019, to March 31st, 2021 were obtained from the GV Archive. Generalized linear mixed-effects models in the form of Poisson regression analysis were utilized to estimate the state-specific rates of GV. Nationally, GV rates were 30% higher between March 01, 2020, and March 31, 2021 (during the pandemic), compared to the same period in 2019 (before the pandemic) [intensity ratio (IR) = 1.30; 95% CI 1.29, 1.32; p < 0.0001]. The risk of GV was significantly higher in 28 states and significantly lower in only one state. National and state-specific rates of GV were higher during the COVID-19 pandemic compared to the same timeframe 1 year prior. State-specific steps to mitigate violence, or at a minimum adequately prepare for its toll during the COVID-19 pandemic, should be taken.

## Introduction

The coronavirus disease 2019 (COVID-19) global pandemic and the policies implemented to address it significantly impacted the psychological, physical, emotional, and financial well-being of almost all individuals living in the United States (U.S.)^1,2^. As of June 17th, 2021, the U.S. had 33 million confirmed cases of COVID-19 and more than 600,000 deaths despite stay-at-home orders enacted in March 2020 and continued emphasis on social distancing, hygiene methods and vaccination. As of April 20, 2020, 42 states and the District of Colombia were under stay-at-home advisories or shelter-in-place policies, affecting approximately 96% of the population in the U.S.^3^. While the purpose of these orders was to decrease disease transmission, there were unintended consequences. The orders forced businesses to close, leaving millions unemployed^4^. Furthermore, the physical distancing necessary to curb transmission of the virus also disrupted social support networks. Combined, these forces may have created a climate with the potential to increase firearm-related suicides^5^. In addition, unemployment and financial strain, increased unscheduled time, and increased substance abuse may result in increased risk-taking behaviors, elevating the risk of violent crimes^5,6^.

An assessment of gun purchases in the U.S. revealed that during the COVID-19 stay-at-home orders, there was a significant increase in the number of criminal background checks for gun purchases. From March until June 2020, the FBI conducted 13,674,878 background checks for gun purchases, indicating a 42% increase in comparison to the same timeframe in 2019^7^. This suggests greater firearm access among the U.S. population during the pandemic, and access to firearms is independently associated with the risk of gun-related suicide and homicide^8^. A meta-analysis of 16 observational studies found a threefold greater odds of suicide and a twofold greater odds of homicide among participants who had firearm access compared to those who did not^9^.

Despite the increased risk factors for GV, some cities are reporting a paradoxical decrease in GV perhaps due to the stay-at-home orders. However, other cities are experiencing a rapid increase in overall crime. Recent data support the notion that in some U.S. cities, gun violence (GV) is reaching an all-time high. Philadelphia is just one example of this, with 141 shootings in March of 2020, “making it Philadelphia's worst March for [GV] in 5 years”^10^. In addition, numbers of injured individuals from GV rose from 23,000 in 2014 to 31,000 in 2017; it declined to 28,000 in 2018 and slightly rose to 30,000 in 2019 before dramatically increasing to 39,000 in 2020. These trends were similar for deaths (Supplementary file 1)^11^. To date, no comprehensive study has systematically assessed GV rates during the COVID-19 pandemic and stay-at-home orders across all states in the U.S. We hypothesized that there would be an increase in GV rates during the COVID-19 pandemic across the U.S. in comparison to the pre-pandemic year of 2019.

## Methods

### Data sources

The Gun Violence Archive (GVA) is an independent not-for-profit organization that compiles comprehensive and accurate information about GV in the US^11^https://www.gunviolencearchive.org. The GVA provided all data corresponding to gun-related injuries and deaths in the U.S. from February 1st, 2019, to March 31st, 2021. This data is collected via law enforcement, media, government, and commercial sources and then verified by independent researchers. To obtain the dataset, ESH completed a Data Request Form as directed by the GVA. The GVA provided comprehensive comma-separated values file documents, consolidating all gun violence events through the study period. No written agreements related to confidentiality or data use were necessary. Information about daily events, location of the incident (street address, city, and state) and the number of individuals killed or injured were the data points of interest. No clearances were required because all incidents are publicly and freely available online. Two authors (ESH and AES) randomly selected 1% of incidents and verified the accuracy of the data using the news report published and reporting about the incidents.

The COVID-19 Dashboard by the Centers for Systems Science and Engineering (CSSE) at Johns Hopkins University provides freely available data related to COVID-19^4^. State population data and other demographic characteristics (age, sex, and race) were extracted from the U.S. Census Bureau, Department of Commerce database^12^.

### Outcome and measures

The primary outcome of interest was the rate of GV during the COVID 19 pandemic versus 13-month period prior, both at the state and national levels. The results were reported as intensity ratios (IR). GV event rates were measured as counts of gun-related injuries and/or deaths per 1,000,000 population. The secondary outcome was the correlation of GV events and the number of COVID-19 cases at the state level.

### Statistical analysis

Our data set consists of daily counts of GV events within each of the 50 states and the District of Columbia from January 01, 2019, through March 31, 2021. We combined the daily data from this 27-month period into bi-monthly amounts (January 01, 2019, through January 15, 2019; January 16, 2019, through January 31, 2019; etc.), which yielded 54 time intervals. Although we were interested in comparing the rate of GV between the 13 months of March 01, 2020, through March 31, 2021 (during the pandemic) to the 13 months of February 01, 2019, through February 29, 2020 (prior to the pandemic), we fit the entire time series (January 01, 2019, through March 31, 2021) to improve numerical stability.

For the primary analysis to estimate the IR of GV comparing pre-pandemic and pandemic periods, we applied a generalized linear mixed-effects model in the form of a Poisson regression analysis with a logarithm link function for each state with the following model characteristics:

- the logarithm of the state’s population as an offset
- cubic polynomial splines to model the event rate over the 54 time intervals
- knot points for splines at months 3, 6, 9, 12, 15, 18, 21, and 24

We embedded a cubic polynomial spline function within the Poisson regression of the generalized linear mixed-effects model to model the GV counts during the 27-month observation period (January 01, 2019, through March 31, 2021). The cubic polynomial spline function we applied consists of nine segments with knot points selected at 3-month intervals. Let $t$ denote the elapsed number of months since January 01, 2019, such that $t\in \left[\mathrm{0,27}\right]$, and we designate the knot points as

$${t}_{\left(1\right)}=3,{t}_{\left(2\right)}=6,{t}_{\left(3\right)}=9,{t}_{\left(4\right)}=12,{t}_{\left(5\right)}=15,{t}_{\left(6\right)}=18,{t}_{\left(7\right)}=21,{t}_{\left(8\right)}=24$$

The unknown parameters to estimate in the model are an intercept parameter $\left({\beta }_{0}\right)$, a linear parameter $\left({\beta }_{1}\right)$, a quadratic parameter $\left({\beta }_{2}\right)$, and nine cubic parameters $\left({\beta }_{3(1)},{\beta }_{3(2)},\dots ,{\beta }_{3(9)}\right)$. Denoting the GV count as the exponentiated value of $f\left(t\right)$ at time $t$, the cubic polynomial spline function is as follows:

$$\begin{gathered} f\left( t \right) = \beta_{0} + t\beta_{1} + t^{2} \beta_{2} + t^{3} \beta_{3\left( 1 \right)} \qquad 0 \le t \le t_{\left( 1 \right)} \hfill \\ \begin{array}{*{20}c} {f\left( t \right) = \beta_{0} + t\beta_{1} + t^{2} \beta_{2} + t^{3} \beta_{3\left( 1 \right)} } \\ {\quad \quad \quad + \left( {t - t_{\left( 1 \right)} } \right)^{3} \left( {\beta_{3\left( 2 \right)} - \beta_{3\left( 1 \right)} } \right) } \\ \end{array} \quad t_{\left( 1 \right)} \le t \le t_{\left( 2 \right)} \hfill \\ \begin{array}{*{20}c} {f\left( t \right) = \beta_{0} + t\beta_{1} + t^{2} \beta_{2} + t^{3} \beta_{3\left( 1 \right)} } \\ {\quad \quad \quad + \left( {t - t_{\left( 1 \right)} } \right)^{3} \left( {\beta_{3\left( 2 \right)} - \beta_{3\left( 1 \right)} } \right) } \\ {\quad \quad \quad + \left( {t - t_{\left( 2 \right)} } \right)^{3} \left( {\beta_{3\left( 3 \right)} - \beta_{3\left( 2 \right)} } \right)} \\ \end{array} \quad t_{\left( 2 \right)} \le t \le t_{\left( 3 \right)} \hfill \\ \quad \quad \quad \quad \quad \vdots \hfill \\ \begin{array}{*{20}c} {f\left( t \right) = \beta_{0} + t\beta_{1} + t^{2} \beta_{2} + t^{3} \beta_{3\left( 1 \right)} } \\ {\quad \quad \quad + \left( {t - t_{\left( 1 \right)} } \right)^{3} \left( {\beta_{3\left( 2 \right)} - \beta_{3\left( 1 \right)} } \right)} \\ {\quad \quad \quad + \left( {t - t_{\left( 3 \right)} } \right)^{3} \left( {\beta_{3\left( 4 \right)} - \beta_{3\left( 3 \right)} } \right)} \\ {\quad \quad \quad + \left( {t - t_{\left( 4 \right)} } \right)^{3} \left( {\beta_{3\left( 5 \right)} - \beta_{3\left( 4 \right)} } \right)} \\ {\quad \quad \quad + \left( {t - t_{\left( 5 \right)} } \right)^{3} \left( {\beta_{3\left( 6 \right)} - \beta_{3\left( 5 \right)} } \right) } \\ {\quad \quad \quad + \left( {t - t_{\left( 6 \right)} } \right)^{3} \left( {\beta_{3\left( 7 \right)} - \beta_{3\left( 6 \right)} } \right) } \\ {\quad \quad \quad + \left( {t - t_{\left( 7 \right)} } \right)^{3} \left( {\beta_{3\left( 8 \right)} - \beta_{3\left( 7 \right)} } \right) } \\ {\quad \quad \quad + \left( {t - t_{\left( 8 \right)} } \right)^{3} \left( {\beta_{3\left( 9 \right)} - \beta_{3\left( 8 \right)} } \right) } \\ {} \\ \end{array} \quad t_{\left( 8 \right)} \le t \le 27 \hfill \\ \end{gathered}$$

At ${t}_{\left(i\right)}$, the *i*th knot point, $i=\mathrm{1,2},\dots ,8$, $f(t)$ has the following properties:

- $$\mathop {\lim }\nolimits_{{t \uparrow t_{{\left( i \right)}} }} f\left( t \right) = \mathop {\lim }\nolimits_{{t \downarrow t_{{\left( i \right)}} }} f\left( t \right)$$
- $$\mathop{\lim }\nolimits_{{t \uparrow t_{{\left( i \right)}} }} f^{\prime}\left( {\text{t}} \right){{ = }} \mathop{\lim }\nolimits_{{{\text{t}} \downarrow {\text{t}}_{{\left( {\text{i}} \right)}} }} {\text{f}}^{\prime}\left( {\text{t}} \right)$$
- $$\mathop {\lim }\nolimits_{{t \uparrow t_{{\left( i \right)}} }} f^{\prime\prime}\left( {\text{t}} \right){{ = }}\mathop {\lim }\nolimits_{{{\text{t}} \downarrow {\text{t}}_{{\left( {\text{i}} \right)}} }} {\text{f}}^{\prime\prime}\left( {\text{t}} \right)$$

In other words, $f\left(t\right)$ and its first two derivatives are continuous at each knot point.

We fit cubic polynomial splines to account for the curvilinear changes in the event rate over the 27-month period within each state. As is typical of cubic polynomial splines, we imposed the smoothing conditions such that the splines and their first two derivatives are continuous at the knot points.

Next, we constructed test statistics based on the model-based estimates to construct 24 distinct comparisons:

- March 01, 2020 through March 15, 2020 versus March 01, 2019 through March 15, 2019
- March 16, 2020 through March 31, 2020 versus March 16, 2019 through March 31, 2019
- …
- February 16, 2021, through February 28, 2021, versus February 16, 2020, through February 29, 2020

We did not apply any multiple comparison adjustments. More importantly, we constructed an overall comparison of the 13-month pandemic period March 01, 2020 through March 31, 2021 versus the 13-month pre-pandemic period February 01, 2019 through February 29, 2020.

For the overall U.S. analysis, we applied a generalized linear mixed-effects model in the form of a Poisson regression analysis as described above with three additional features:

- a first-order autoregressive process to account for the correlation across the time intervals
- random effect for state
- four covariates based on census data (each state’s median age, Black-White ratio, Hispanic-White ratio, and male–female ratio).

For the secondary outcome of correlation of the number of COVID-19 cases and the number of GV events, we constructed a data set with the daily numbers of COVID-19 and GV cases from each state for the period February 01, 2020, through March 10, 2021. We constructed a bivariate generalized linear mixed-effects model in the form of Poisson regression with a cubic polynomial function. The statistical model is bivariate because it simultaneously analyzes the two sets of correlated longitudinal variables (COVID-19 cases and gun-violence events). For the analysis of each state’s data, we included a time-dependent binary variable (no/yes) as to the status of the state’s stay-at-home order. For the analysis of the overall U.S. data, we included the four covariates (state’s median age, Black-White ratio, Hispanic-White ratio, and male–female ratio) based on census data. In all these bivariate models, we estimated the correlation between COVID-19 cases and gun-violence events.

Comparison of the spatial distributions of GV during the pandemic vs. pre-pandemic was performed using spatial relative risk surfaces^13^. Statistical significance level was set at p < 0.01 for spatial relative risk surface and p < 0.05 for all other analyses. All analyses were performed with the R statistical language (R Development Core Team 2020 Version 3.0.6) and SAS Version 9.4

### Consent for publication

No consent to publish was needed for this study as we did not use any details, images or videos related to individual participants.

## Results

We identified 92,731 gun violence events resulting in injury or death in the U.S. from January 01, 2019, through March 31, 2021. Table 1 indicates the numbers of events according to the 13-month pre-pandemic period and the 13-month pandemic period.

**Table 1:** The table indicates that there was a 31.2% increase in the number of incidents between the pandemic and pre-pandemic periods.

	Incidents	Deaths	Injured
Pre-pandemic (February 01, 2019, through February 29, 2020)	38,919	16,687	32,348
Pandemic (March 01, 2020 through March 31, 2021)	51,063	21,504	43,288

### Risk of gun violence during COVID-19 pandemic period

**Figure 1:**
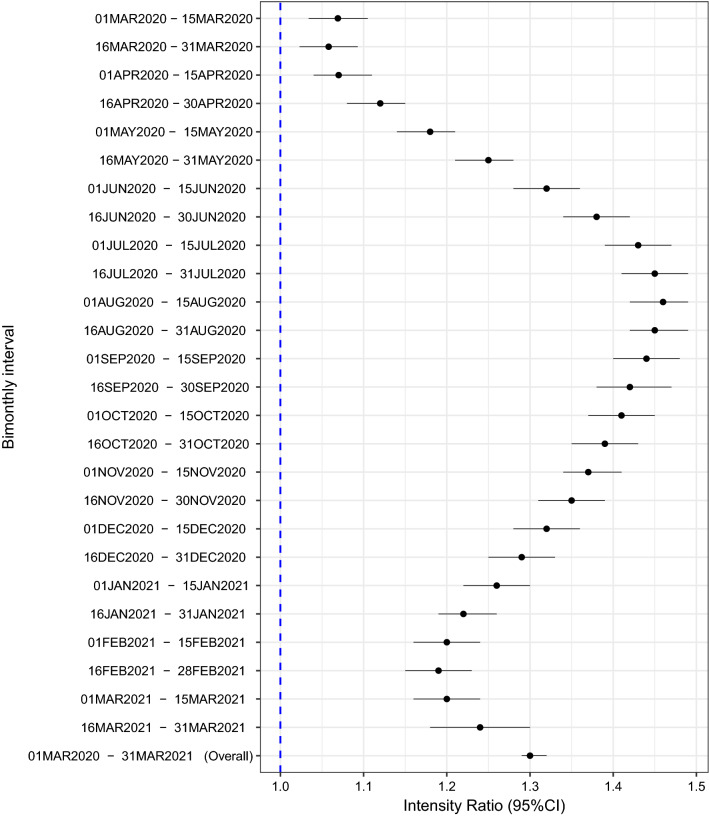
**Bimonthly intervals for intensity of GV during and before the pandemic**. Bimonthly interval-specific intensity ratio (IR) and their 95% confidence intervals of GV. The dashed blue line in the forest plot represents the null estimate. IR greater than one indicates higher intensity of GV durin﻿g COVID-19 pandemic compared to pre-pandemic.

On a national level, the risk of GV was 30% higher during 13-month pandemic period March 01, 2020 through March 31, 2021, compared to the 13-month pre-pandemic period February 01, 2019 through February 29, 2020 (IR = 1.30; 95% CI 1.29, 1.32; p < 0.0001, Fig. 1). The risk of GV in the U.S. was consistently higher for all bi-monthly intervals from March 01, 2020 through March 31, 2021, in comparison to the baseline period (Fig. 1).

**Figure 2:**
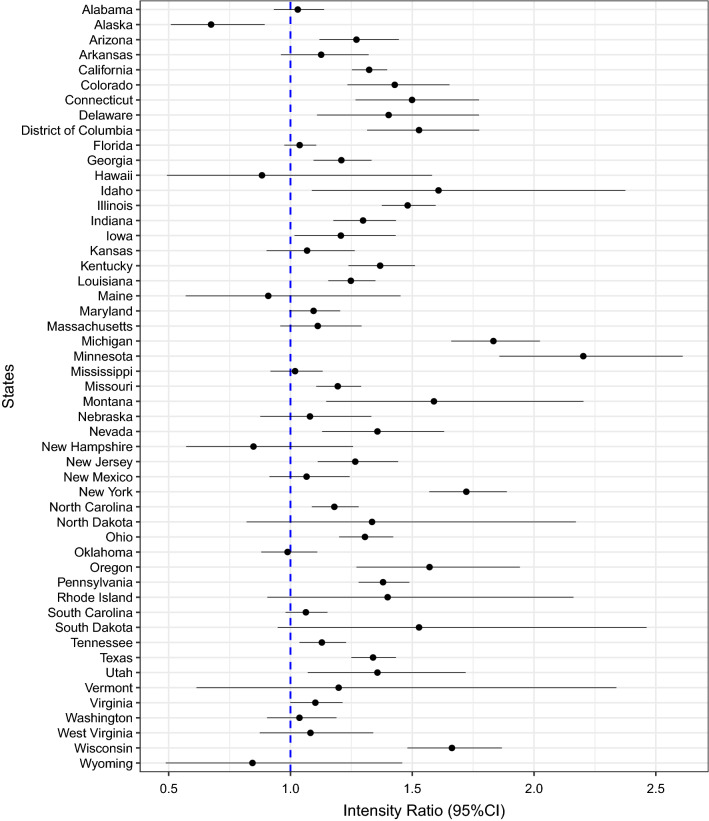
**State-specific intensity of GV during and before the pandemic**. State-specific intensity ratio (IR) and their 95% confidence intervals of GV. The dashed blue line in the forest plots represents the null estimate. IR greater than one indicates ﻿higher intensity of GV during the COVID-19 pandemic compared to the pre-pandemic period.

States with a significantly higher risk of GV during the pandemic compared to the same period pre-pandemic included: Arizona, California, Colorado, Connecticut, Delaware, District of Columbia, Georgia, Idaho, Illinois, Indiana, Iowa, Kentucky, Louisiana, Michigan, Minnesota, Missouri, Montana, Nevada, New Jersey, New York, North Carolina, Ohio, Oregon, Pennsylvania, Tennessee, Texas, Utah, and Wisconsin (Fig. 2). Conversely, Alaska was the only state that showed a lower risk of GV during the pandemic than pre-pandemic. Complete estimates of state-specific bimonthly rates of GV during pandemic compared to the pre-pandemic periods are reported in Supplementary file 1.

**Figure 3:**
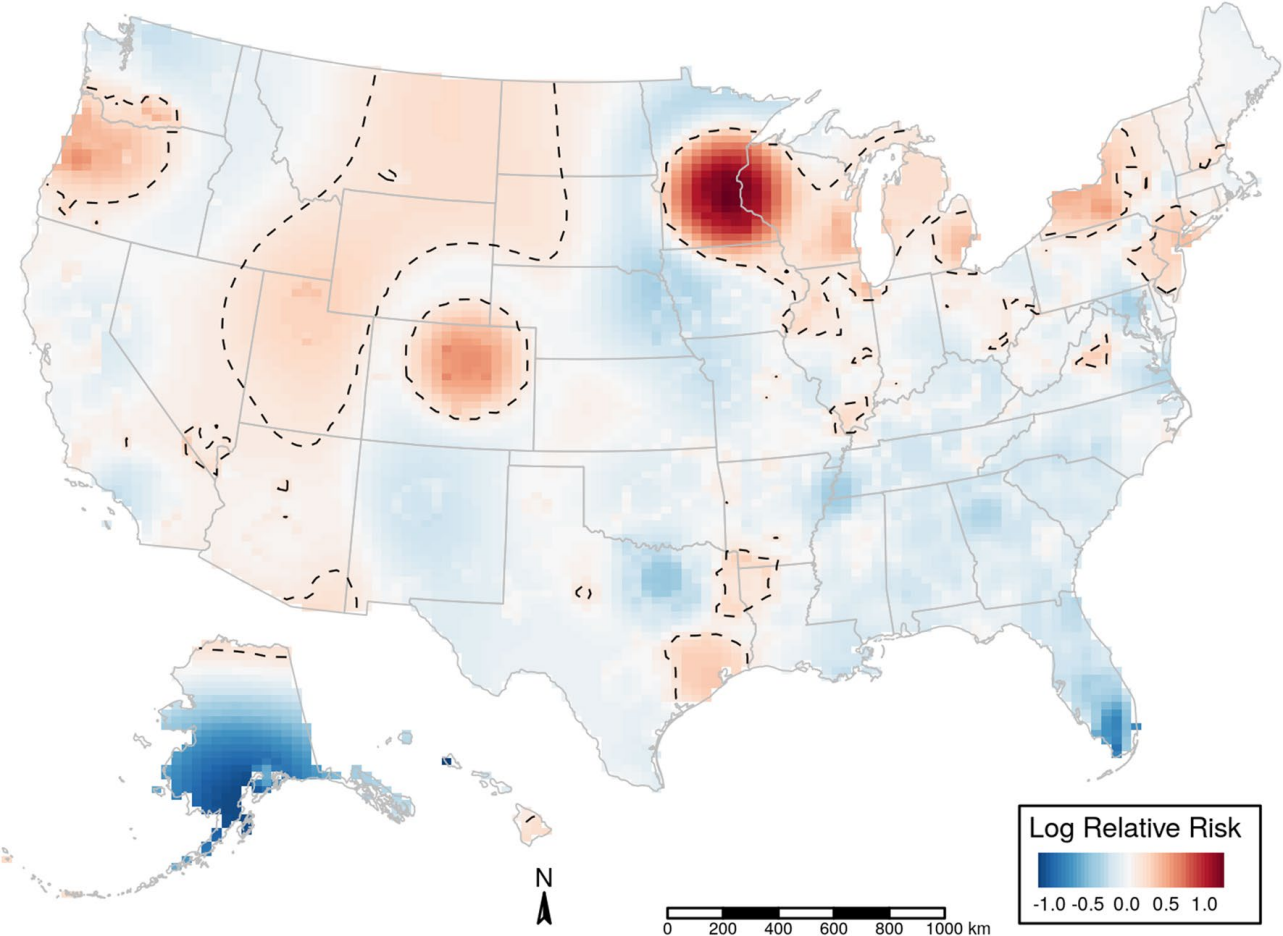
**Spatial relative risk of gun violence during the pandemic vs. pre-pandemic.** Map shows the intensity (or risk) difference which was estimated by comparing the smoothed intensity of GV events during the pandemic (March 01, 2020, through March 31, 2021) vs. before the pandemic (February 01, 2019, through February 29, 2020) across 51 states. If the difference is ~ 0, the risk of GV is unrelated to spatial location. Evidence of spatial variation in risk occurs where the intensities differ. Difference values > 0 indicate higher risk, and values < 0 indicate lower risk. Dotted lines highlight areas of significantly higher risk of GV during the pandemic.

We examined the spatial distribution of GV using the global position system (GPS) coordinates of the event. Within some states, there were hotspot of higher GV risk (p < 0.01). These spatial clusters are heterogenous (Fig. 3).

### Correlation of COVID-19 cases and GV rates per state

In the correlation analysis, overall a modest correlation between COVID-19 cases and GV rates (r = 0.32, p = 0.0001) was observed. However, in the multivariable analysis adjusting for the population, mean age, gender proportion, and lockdown orders, we only observed a relatively weak positive correlation between COVID-19 cases and GV in Hawaii (r = 0.14; 95% CI 0.04, 0.23; p = 0.006). There were no other state with statistically significant positive correlations between COVID-19 cases and GV.

## Discussion

We found a strong association between the COVID-19 pandemic time frame and an increase in gun violence in the U.S. compared to the pre-pandemic period. We identified significantly higher rates of GV in 28 states. While stay-at-home orders and social distancing measures are vital to contain the spread of COVID 19, we also need to be aware of the unintended social and economic stressors that may lead to gun violence.

The current increase in GV seen across the U.S. may be attributed to (1) increased psychological stress resulting from COVID-19 or (2) the increase in firearm sales. Recent reports suggest a substantial increase in the burden of depressive symptoms in the U.S. associated with the COVID-19^14,15^. This could potentially lead to an increase in fire-arm-related suicides. It is hypothesized that psychological stress and depressive symptoms may be due to the heightened need to maintain physical distancing from family and friends, thereby limiting social interactions. Our data did not directly address this, but there were also protests against racial injustice during this same period. Although the protests were largely peaceful, there were reports in the media about gun incidents among protestors and counter protestors.

Increased access to firearms is another plausible reason for the higher rates of GV during the pandemic. Since the COVID-19 pandemic also led to the closure of businesses, the National Instant Criminal Background Check System (NICS) recorded a surge in gun sales driven by public panic and unfounded fears that guns would soon be in short supply. An estimated 41% increase in sales of handguns were recorded in March 2020 as compared to March 2019^7^. Several states determined such stores to be essential businesses, leading them to remain open.

### Public health implications

Gun violence is a frequently ignored public health epidemic. The spike in gun violence in the era of the COVID-19 pandemic come as a stark reminder that we cannot afford to ignore it any longer. Unlike the COVID-19 pandemic, which still carries a low threat of death in children and young adults, the threat of being killed by a firearm is a much more significant concern in this population.

### Strengths and limitations

Our study included several strengths and limitations. It is the first study to report the rate of gun-related incidents in the United States during the COVID-19 pandemic and compare them to the pre-pandemic period. Including data on all incidents reported to police across all 50 states is another strength of the study. Due to the interdependence between events, auto-regressive covariance structure, non-linear effects, and non-Gaussian distributions, the Poisson GLMM model with cubic polynomial spline we used for the data analysis was appropriate and advantageous in estimating time and space events, including removing the confounding effect of both time and space and non-linearity^16^. The geospatial analysis method we fitted is granular. We did not map the state-specific counts of events to avoid aggregation of data, thereby preventing ecological fallacy and modifiable areal unit problems. One limitation of using police reports is that the homicide/suicide investigation is often still pending at the time of the report. Therefore, it was not possible to determine whether these recent incidents were due to suicide or homicide. In addition, although we adjusted for other major confounders in the models (state’s median age, race composition of the state), it is possible that residual confounding remained and could bias the estimates observed. Lastly, the high-order polynomial models we used to estimate the risk of GV have the disadvantages of model overfitting. Despite this, our study remains strong. It is the first of its kind to identify a substantial change in fire-arm-related incidents during the pandemic and estimate the relative spatial risk using GPS location of the events.

### Conclusion

Overall, U.S. and state-specific rates of gun violence are higher during the COVID-19 pandemic compared to the same period pre-pandemic.

## Supplementary Information

Supplementary Information.
