# Supplementary material for: Gun violence incidence during the COVID-19 pandemic is higher than before the pandemic in the United States
Source: Sci Rep. 2021 Oct 21;11:20654. doi: 10.1038/s41598-021-98813-z (PMC8531009; doi:10.1038/s41598-021-98813-z)
Supplement: Supplementary file 1 — Supplementary Information. [file 41598_2021_98813_MOESM1_ESM.pdf]

Supplementary information to:

Gun Violence Incidence During the COVID-19 Pandemic Is Higher Than Before the Pandemic in the United States

Paddy Ssentongo, MD, PhD, MPH[1,2]§*, Claudio Fronterre, PhD[3]*, Anna E. Ssentongo, MPH[1,4]*, Shailesh Advani, MD, PhD[5,6]*, Emily S. Heilbrunn, BS[1], Joshua P. Hazelton, DO,[4] John S. Oh[4], Jennifer S. McCall-Hosenfeld, MD, MSc[7]¶, Vernon M. Chinchilli, PhD[1]¶

*¶ Contributed Equally

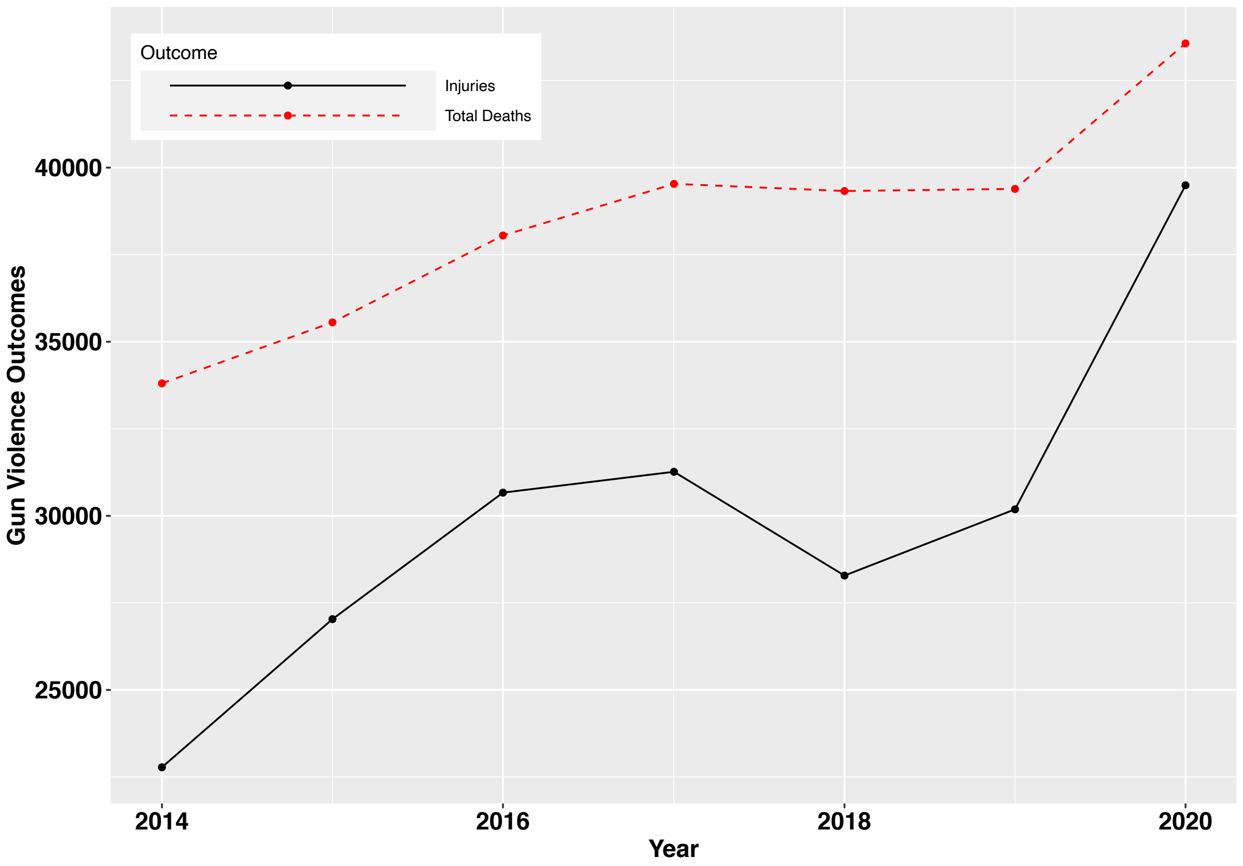

Supplemental Figure 1. Trends of injuries and deaths from gun violence in the United States. Source of data: https://www.gunviolencearchive.org/

Descriptive Statistics for Pre-Pandemic and Pandemic (January 01, 2019 through March 31, 2021)

The MEANS Procedure

Variable	Sum
n_incidents	92731
n_killed	39449
n_injured	77760

Descriptive Statistics for Pre-Pandemic (February 01, 2019 through February 29, 2020)

The MEANS Procedure

Variable	Sum
n_incidents	38919
n_killed	16687
n_injured	32348

Descriptive Statistics for Pandemic (March 01, 2020 through March 31, 2021)

The MEANS Procedure

Variable	Sum
n_incidents	51063
n_killed	21504
n_injured	43288

Alabama
Bimonthly Data

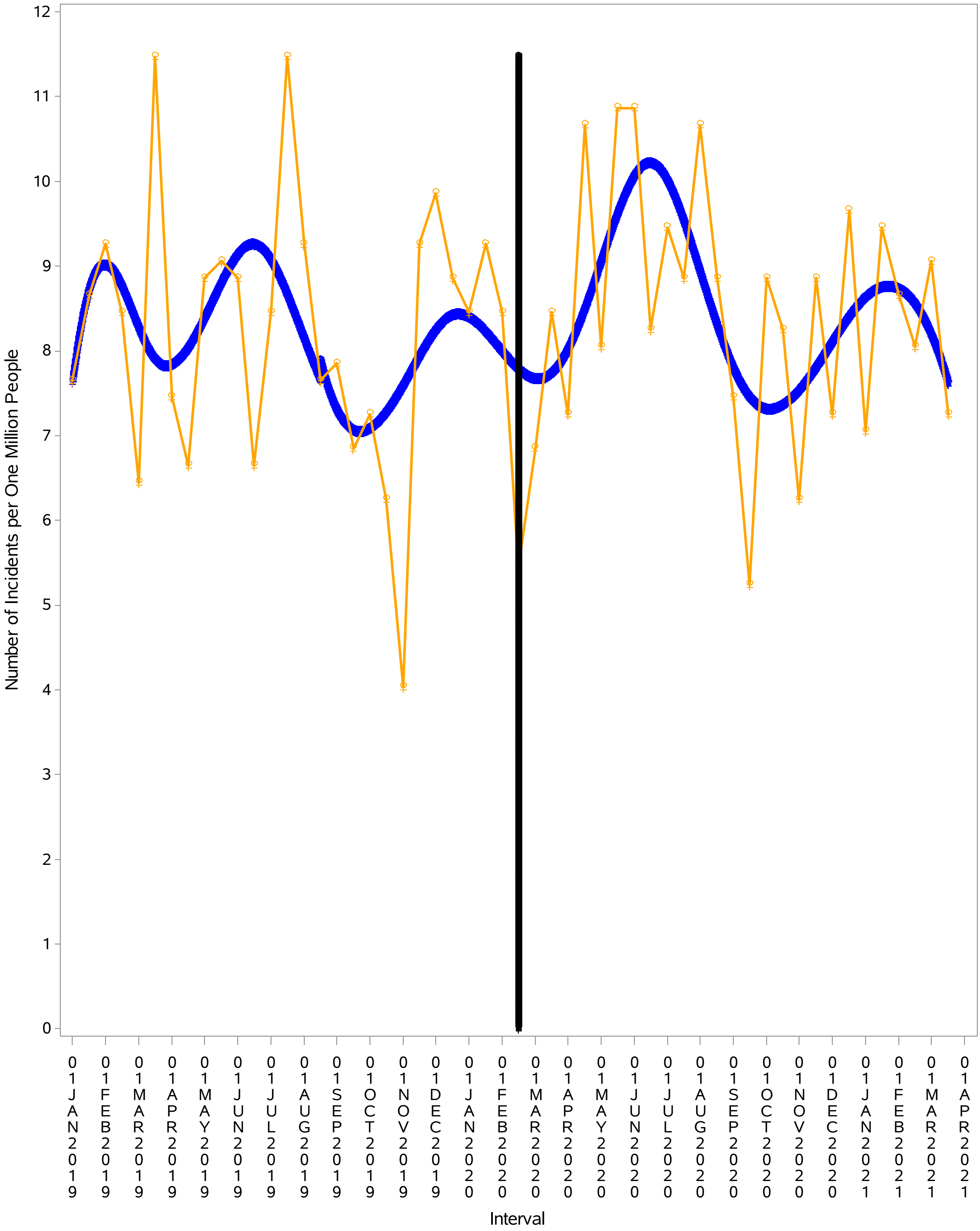

Alabama
Bimonthly Data

Comparison	IntensityRatio	IntensityRatio_LowerCL	IntensityRatio_UpperCL	P_Value
[01MAR2020 thru 15MAR2020] vs [01MAR2019 thru 15MAR2019]	0.928	0.735	1.170	0.5181
[16MAR2020 thru 31MAR2020] vs [16MAR2019 thru 31MAR2019]	0.979	0.771	1.245	0.8621
[01APR2020 thru 15APR2020] vs [01APR2019 thru 15APR2019]	1.024	0.804	1.304	0.8449
[16APR2020 thru 30APR2020] vs [16APR2019 thru 30APR2019]	1.057	0.843	1.325	0.6231
[01MAY2020 thru 15MAY2020] vs [01MAY2019 thru 15MAY2019]	1.080	0.879	1.326	0.4561
[16MAY2020 thru 31MAY2020] vs [16MAY2019 thru 31MAY2019]	1.094	0.895	1.337	0.3725
[01JUN2020 thru 15JUN2020] vs [01JUN2019 thru 15JUN2019]	1.101	0.890	1.362	0.3661
[16JUN2020 thru 30JUN2020] vs [16JUN2019 thru 30JUN2019]	1.104	0.883	1.380	0.3782
[01JUL2020 thru 15JUL2020] vs [01JUL2019 thru 15JUL2019]	1.104	0.888	1.371	0.3655
[16JUL2020 thru 31JUL2020] vs [16JUL2019 thru 31JUL2019]	1.101	0.898	1.349	0.3463
[01AUG2020 thru 15AUG2020] vs [01AUG2019 thru 15AUG2019]	1.095	0.897	1.336	0.3653
[16AUG2020 thru 31AUG2020] vs [16AUG2019 thru 31AUG2019]	1.085	0.876	1.343	0.445
[01SEP2020 thru 15SEP2020] vs [01SEP2019 thru 15SEP2019]	1.067	0.845	1.348	0.5751
[16SEP2020 thru 30SEP2020] vs [16SEP2019 thru 30SEP2019]	1.054	0.825	1.346	0.667
[01OCT2020 thru 15OCT2020] vs [01OCT2019 thru 15OCT2019]	1.034	0.819	1.306	0.7742
[16OCT2020 thru 31OCT2020] vs [16OCT2019 thru 31OCT2019]	1.011	0.814	1.256	0.9165
[01NOV2020 thru 15NOV2020] vs [01NOV2019 thru 15NOV2019]	0.994	0.804	1.228	0.9512
[16NOV2020 thru 30NOV2020] vs [16NOV2019 thru 30NOV2019]	0.983	0.786	1.230	0.8771
[01DEC2020 thru 15DEC2020] vs [01DEC2019 thru 15DEC2019]	0.983	0.774	1.247	0.8843
[16DEC2020 thru 31DEC2020] vs [16DEC2019 thru 31DEC2019]	0.997	0.786	1.265	0.9816
[01JAN2021 thru 15JAN2021] vs [01JAN2020 thru 15JAN2020]	1.028	0.821	1.287	0.807
[16JAN2021 thru 31JAN2021] vs [16JAN2020 thru 31JAN2020]	1.064	0.849	1.334	0.5794
[01FEB2021 thru 15FEB2021] vs [01FEB2020 thru 15FEB2020]	1.093	0.859	1.392	0.4602
[16FEB2021 thru 28FEB2021] vs [16FEB2020 thru 29FEB2020]	1.099	0.859	1.405	0.4451
[01MAR2020 thru 31MAR2021] vs [01FEB2019 thru 29FEB2020]	1.030	0.932	1.138	0.5577

Alaska
Bimonthly Data

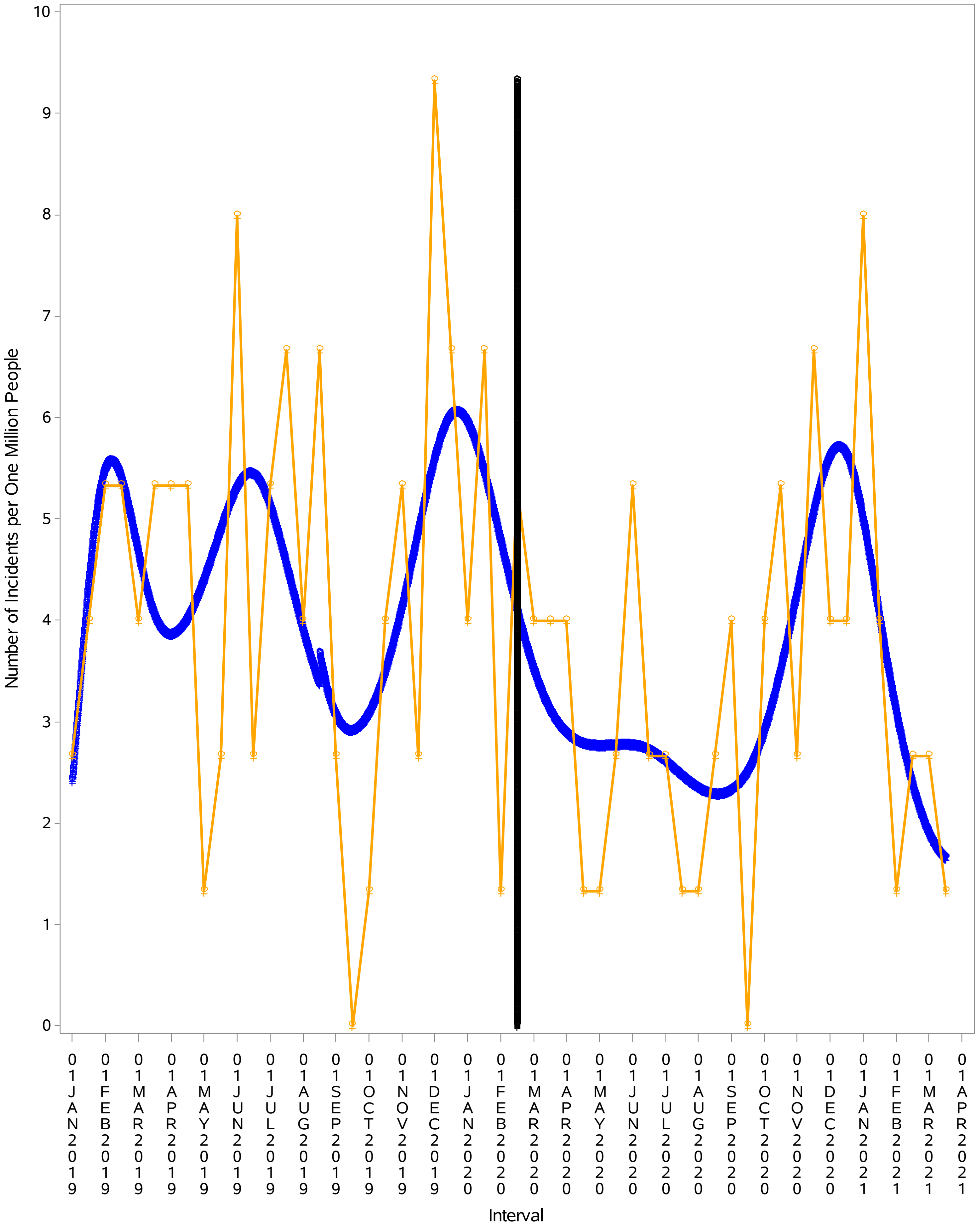

Alaska
Bimonthly Data

Comparison	IntensityRatio	IntensityRatio_LowerCL	IntensityRatio_UpperCL	P_Value
**[01MAR2020 thru 15MAR2020] vs [01MAR2019 thru 15MAR2019]**	0.754	0.409	1.388	0.3556
**[16MAR2020 thru 31MAR2020] vs [16MAR2019 thru 31MAR2019]**	0.769	0.406	1.459	0.4132
**[01APR2020 thru 15APR2020] vs [01APR2019 thru 15APR2019]**	0.748	0.390	1.432	0.3719
**[16APR2020 thru 30APR2020] vs [16APR2019 thru 30APR2019]**	0.694	0.374	1.285	0.2377
**[01MAY2020 thru 15MAY2020] vs [01MAY2019 thru 15MAY2019]**	0.628	0.351	1.123	0.1135
**[16MAY2020 thru 31MAY2020] vs [16MAY2019 thru 31MAY2019]**	0.566	0.315	1.019	0.0577
**[01JUN2020 thru 15JUN2020] vs [01JUN2019 thru 15JUN2019]**	0.521	0.278	0.979	0.0431
**[16JUN2020 thru 30JUN2020] vs [16JUN2019 thru 30JUN2019]**	0.501	0.259	0.969	0.0405
**[01JUL2020 thru 15JUL2020] vs [01JUL2019 thru 15JUL2019]**	0.509	0.268	0.968	0.04
**[16JUL2020 thru 31JUL2020] vs [16JUL2019 thru 31JUL2019]**	0.544	0.297	0.997	0.0489
**[01AUG2020 thru 15AUG2020] vs [01AUG2019 thru 15AUG2019]**	0.602	0.331	1.094	0.094
**[16AUG2020 thru 31AUG2020] vs [16AUG2019 thru 31AUG2019]**	0.679	0.359	1.285	0.2277
**[01SEP2020 thru 15SEP2020] vs [01SEP2019 thru 15SEP2019]**	0.761	0.382	1.517	0.4284
**[16SEP2020 thru 30SEP2020] vs [16SEP2019 thru 30SEP2019]**	0.862	0.422	1.760	0.6773
**[01OCT2020 thru 15OCT2020] vs [01OCT2019 thru 15OCT2019]**	0.950	0.489	1.844	0.8763
**[16OCT2020 thru 31OCT2020] vs [16OCT2019 thru 31OCT2019]**	1.005	0.558	1.812	0.9854
**[01NOV2020 thru 15NOV2020] vs [01NOV2019 thru 15NOV2019]**	1.039	0.607	1.778	0.887
**[16NOV2020 thru 30NOV2020] vs [16NOV2019 thru 30NOV2019]**	1.039	0.606	1.781	0.8868
**[01DEC2020 thru 15DEC2020] vs [01DEC2019 thru 15DEC2019]**	1.003	0.573	1.759	0.9901
**[16DEC2020 thru 31DEC2020] vs [16DEC2019 thru 31DEC2019]**	0.934	0.535	1.631	0.8053
**[01JAN2021 thru 15JAN2021] vs [01JAN2020 thru 15JAN2020]**	0.838	0.485	1.449	0.5184
**[16JAN2021 thru 31JAN2021] vs [16JAN2020 thru 31JAN2020]**	0.737	0.402	1.353	0.3169
**[01FEB2021 thru 15FEB2021] vs [01FEB2020 thru 15FEB2020]**	0.648	0.322	1.303	0.217
**[16FEB2021 thru 28FEB2021] vs [16FEB2020 thru 29FEB2020]**	0.580	0.275	1.222	0.1478
**[01MAR2020 thru 31MAR2021] vs [01FEB2019 thru 29FEB2020]**	0.674	0.509	0.894	0.0073

Arizona
Bimonthly Data

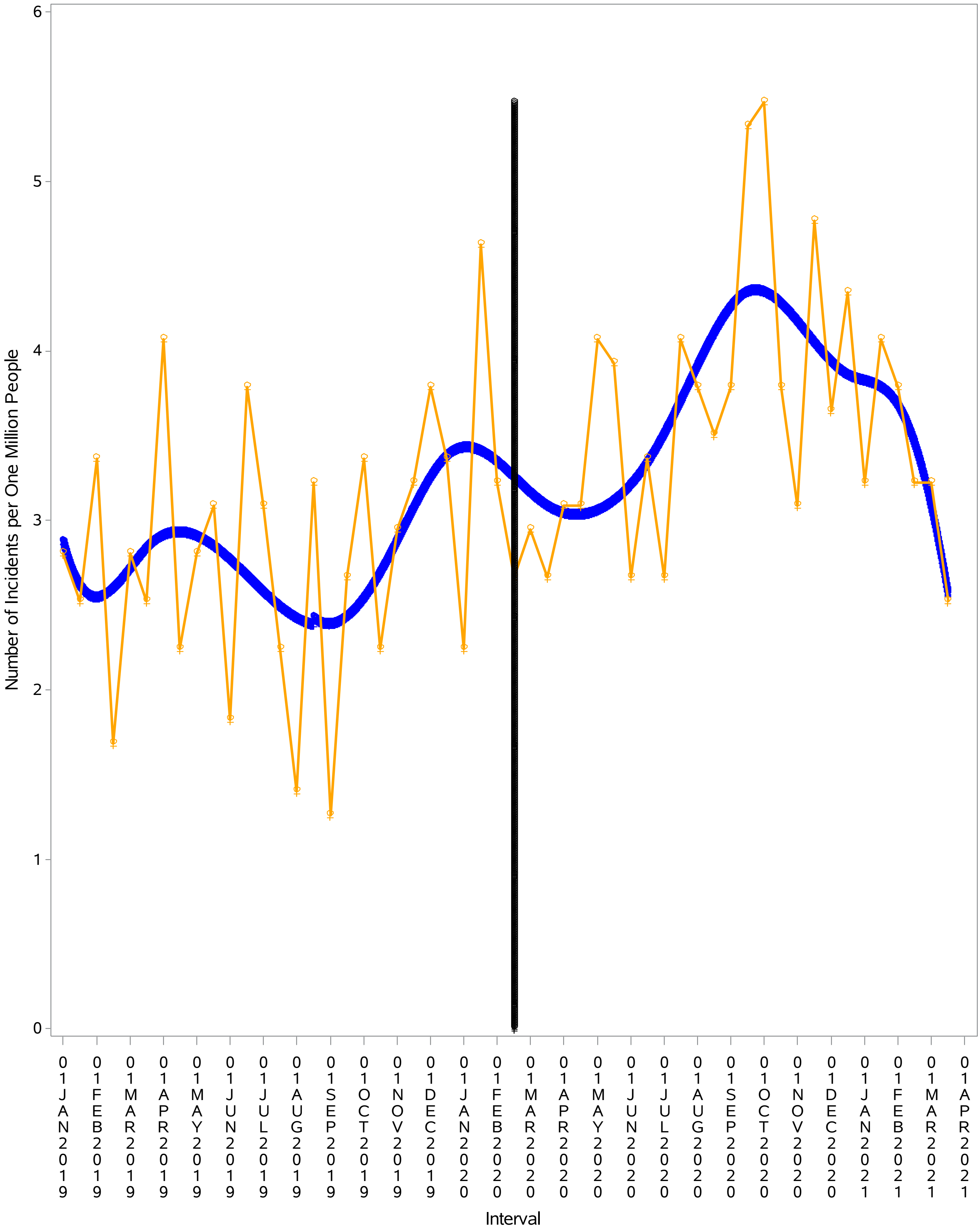

Arizona
Bimonthly Data

Comparison	IntensityRatio	IntensityRatio_LowerCL	IntensityRatio_UpperCL	P_Value
**[01MAR2020 thru 15MAR2020] vs [01MAR2019 thru 15MAR2019]**	1.165	0.859	1.580	0.3189
**[16MAR2020 thru 31MAR2020] vs [16MAR2019 thru 31MAR2019]**	1.088	0.798	1.483	0.585
**[01APR2020 thru 15APR2020] vs [01APR2019 thru 15APR2019]**	1.045	0.764	1.430	0.7761
**[16APR2020 thru 30APR2020] vs [16APR2019 thru 30APR2019]**	1.035	0.771	1.390	0.8151
**[01MAY2020 thru 15MAY2020] vs [01MAY2019 thru 15MAY2019]**	1.052	0.801	1.382	0.709
**[16MAY2020 thru 31MAY2020] vs [16MAY2019 thru 31MAY2019]**	1.094	0.835	1.434	0.5057
**[01JUN2020 thru 15JUN2020] vs [01JUN2019 thru 15JUN2019]**	1.160	0.868	1.552	0.3074
**[16JUN2020 thru 30JUN2020] vs [16JUN2019 thru 30JUN2019]**	1.250	0.919	1.701	0.1499
**[01JUL2020 thru 15JUL2020] vs [01JUL2019 thru 15JUL2019]**	1.363	1.011	1.838	0.0427
**[16JUL2020 thru 31JUL2020] vs [16JUL2019 thru 31JUL2019]**	1.489	1.131	1.961	0.0056
**[01AUG2020 thru 15AUG2020] vs [01AUG2019 thru 15AUG2019]**	1.616	1.247	2.094	0.0006
**[16AUG2020 thru 31AUG2020] vs [16AUG2019 thru 31AUG2019]**	1.723	1.321	2.247	0.0002
**[01SEP2020 thru 15SEP2020] vs [01SEP2019 thru 15SEP2019]**	1.784	1.343	2.370	0.0002
**[16SEP2020 thru 30SEP2020] vs [16SEP2019 thru 30SEP2019]**	1.788	1.329	2.405	0.0003
**[01OCT2020 thru 15OCT2020] vs [01OCT2019 thru 15OCT2019]**	1.711	1.290	2.268	0.0004
**[16OCT2020 thru 31OCT2020] vs [16OCT2019 thru 31OCT2019]**	1.589	1.225	2.059	0.0008
**[01NOV2020 thru 15NOV2020] vs [01NOV2019 thru 15NOV2019]**	1.448	1.127	1.860	0.0047
**[16NOV2020 thru 30NOV2020] vs [16NOV2019 thru 30NOV2019]**	1.315	1.012	1.710	0.041
**[01DEC2020 thru 15DEC2020] vs [01DEC2019 thru 15DEC2019]**	1.209	0.914	1.599	0.1778
**[16DEC2020 thru 31DEC2020] vs [16DEC2019 thru 31DEC2019]**	1.141	0.861	1.512	0.3494
**[01JAN2021 thru 15JAN2021] vs [01JAN2020 thru 15JAN2020]**	1.115	0.851	1.460	0.4199
**[16JAN2021 thru 31JAN2021] vs [16JAN2020 thru 31JAN2020]**	1.110	0.843	1.460	0.449
**[01FEB2021 thru 15FEB2021] vs [01FEB2020 thru 15FEB2020]**	1.099	0.819	1.475	0.5188
**[16FEB2021 thru 28FEB2021] vs [16FEB2020 thru 29FEB2020]**	1.060	0.785	1.432	0.6962
**[01MAR2020 thru 31MAR2021] vs [01FEB2019 thru 29FEB2020]**	1.271	1.119	1.445	0.0005

Arkansas
Bimonthly Data

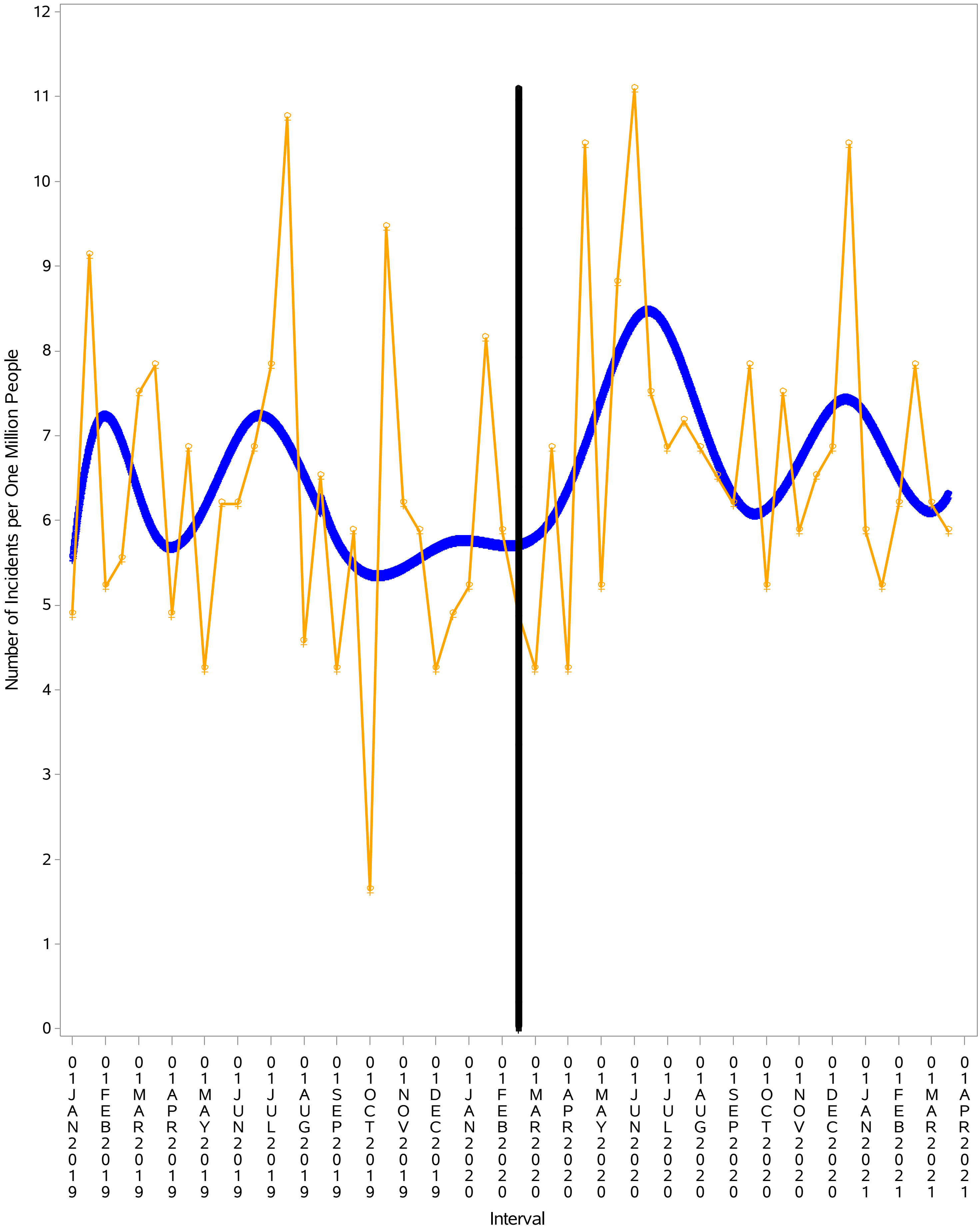

Arkansas
Bimonthly Data

Comparison	IntensityRatio	IntensityRatio_LowerCL	IntensityRatio_UpperCL	P_Value
**[01MAR2020 thru 15MAR2020] vs [01MAR2019 thru 15MAR2019]**	0.920	0.634	1.334	0.6528
**[16MAR2020 thru 31MAR2020] vs [16MAR2019 thru 31MAR2019]**	1.032	0.701	1.518	0.8699
**[01APR2020 thru 15APR2020] vs [01APR2019 thru 15APR2019]**	1.123	0.760	1.658	0.5533
**[16APR2020 thru 30APR2020] vs [16APR2019 thru 30APR2019]**	1.180	0.821	1.698	0.3621
**[01MAY2020 thru 15MAY2020] vs [01MAY2019 thru 15MAY2019]**	1.208	0.870	1.678	0.2526
**[16MAY2020 thru 31MAY2020] vs [16MAY2019 thru 31MAY2019]**	1.211	0.882	1.662	0.2304
**[01JUN2020 thru 15JUN2020] vs [01JUN2019 thru 15JUN2019]**	1.196	0.857	1.670	0.2849
**[16JUN2020 thru 30JUN2020] vs [16JUN2019 thru 30JUN2019]**	1.172	0.826	1.665	0.3646
**[01JUL2020 thru 15JUL2020] vs [01JUL2019 thru 15JUL2019]**	1.147	0.816	1.613	0.4212
**[16JUL2020 thru 31JUL2020] vs [16JUL2019 thru 31JUL2019]**	1.124	0.817	1.545	0.4639
**[01AUG2020 thru 15AUG2020] vs [01AUG2019 thru 15AUG2019]**	1.106	0.809	1.510	0.5198
**[16AUG2020 thru 31AUG2020] vs [16AUG2019 thru 31AUG2019]**	1.096	0.784	1.531	0.5847
**[01SEP2020 thru 15SEP2020] vs [01SEP2019 thru 15SEP2019]**	1.093	0.758	1.577	0.6255
**[16SEP2020 thru 30SEP2020] vs [16SEP2019 thru 30SEP2019]**	1.112	0.757	1.635	0.5797
**[01OCT2020 thru 15OCT2020] vs [01OCT2019 thru 15OCT2019]**	1.147	0.795	1.655	0.4552
**[16OCT2020 thru 31OCT2020] vs [16OCT2019 thru 31OCT2019]**	1.187	0.844	1.668	0.3156
**[01NOV2020 thru 15NOV2020] vs [01NOV2019 thru 15NOV2019]**	1.233	0.884	1.718	0.2107
**[16NOV2020 thru 30NOV2020] vs [16NOV2019 thru 30NOV2019]**	1.272	0.896	1.807	0.1733
**[01DEC2020 thru 15DEC2020] vs [01DEC2019 thru 15DEC2019]**	1.295	0.891	1.884	0.1705
**[16DEC2020 thru 31DEC2020] vs [16DEC2019 thru 31DEC2019]**	1.292	0.886	1.883	0.1777
**[01JAN2021 thru 15JAN2021] vs [01JAN2020 thru 15JAN2020]**	1.256	0.876	1.800	0.2085
**[16JAN2021 thru 31JAN2021] vs [16JAN2020 thru 31JAN2020]**	1.200	0.833	1.728	0.3187
**[01FEB2021 thru 15FEB2021] vs [01FEB2020 thru 15FEB2020]**	1.140	0.772	1.683	0.5026
**[16FEB2021 thru 28FEB2021] vs [16FEB2020 thru 29FEB2020]**	1.087	0.732	1.615	0.6726
**[01MAR2020 thru 31MAR2021] vs [01FEB2019 thru 29FEB2020]**	1.126	0.961	1.321	0.138

California

Bimonthly Data

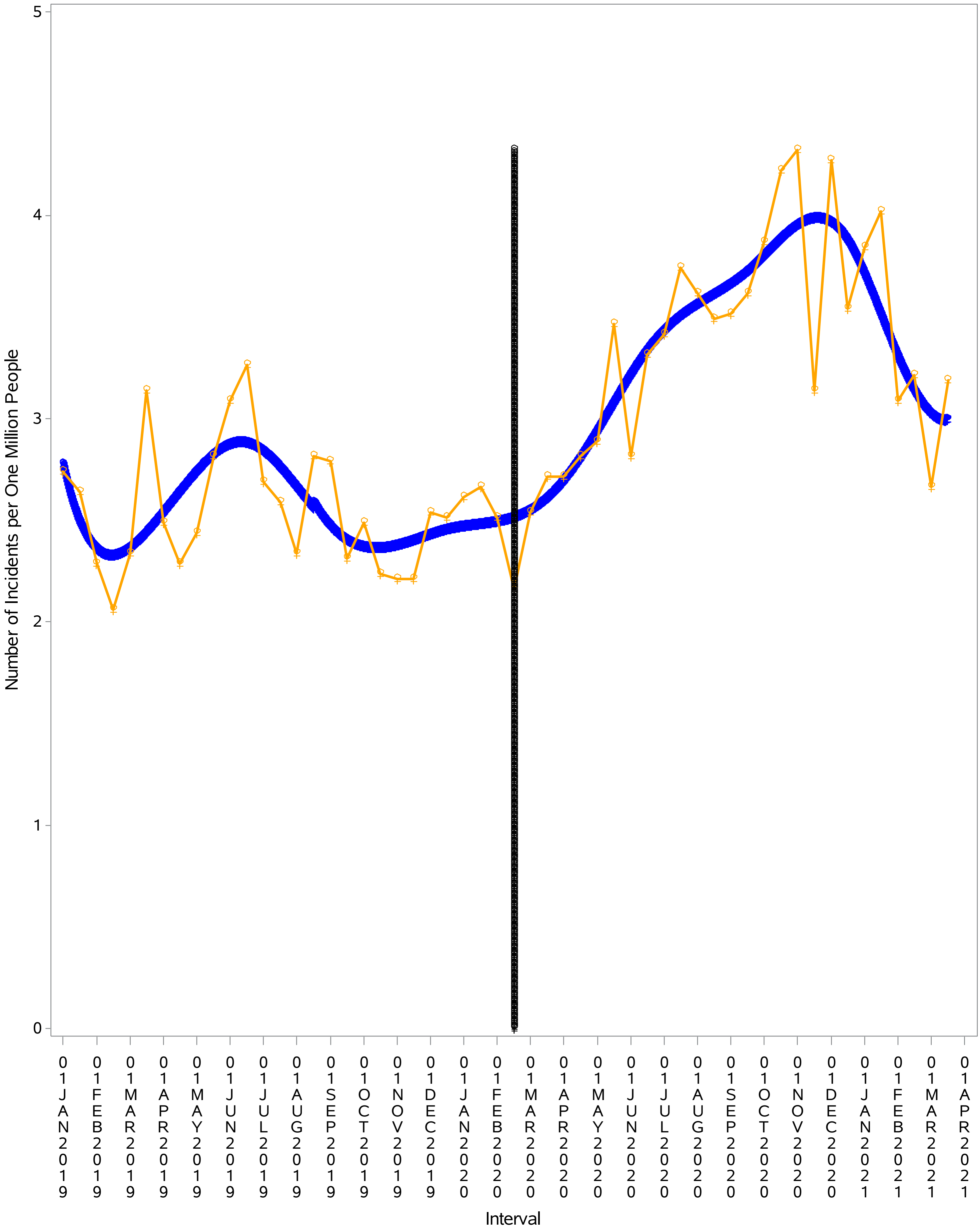

California
Bimonthly Data

Comparison	IntensityRatio	IntensityRatio_LowerCL	IntensityRatio_UpperCL	P_Value
**[01MAR2020 thru 15MAR2020] vs [01MAR2019 thru 15MAR2019]**	1.079	0.942	1.234	0.2644
**[16MAR2020 thru 31MAR2020] vs [16MAR2019 thru 31MAR2019]**	1.068	0.932	1.225	0.3345
**[01APR2020 thru 15APR2020] vs [01APR2019 thru 15APR2019]**	1.063	0.926	1.219	0.3782
**[16APR2020 thru 30APR2020] vs [16APR2019 thru 30APR2019]**	1.064	0.936	1.209	0.3345
**[01MAY2020 thru 15MAY2020] vs [01MAY2019 thru 15MAY2019]**	1.073	0.955	1.206	0.2273
**[16MAY2020 thru 31MAY2020] vs [16MAY2019 thru 31MAY2019]**	1.091	0.975	1.222	0.1266
**[01JUN2020 thru 15JUN2020] vs [01JUN2019 thru 15JUN2019]**	1.119	0.993	1.261	0.0649
**[16JUN2020 thru 30JUN2020] vs [16JUN2019 thru 30JUN2019]**	1.158	1.021	1.313	0.0238
**[01JUL2020 thru 15JUL2020] vs [01JUL2019 thru 15JUL2019]**	1.209	1.069	1.366	0.0032
**[16JUL2020 thru 31JUL2020] vs [16JUL2019 thru 31JUL2019]**	1.270	1.134	1.422	0.0001
**[01AUG2020 thru 15AUG2020] vs [01AUG2019 thru 15AUG2019]**	1.338	1.202	1.490	< 0.0001
**[16AUG2020 thru 31AUG2020] vs [16AUG2019 thru 31AUG2019]**	1.411	1.261	1.578	< 0.0001
**[01SEP2020 thru 15SEP2020] vs [01SEP2019 thru 15SEP2019]**	1.480	1.312	1.671	< 0.0001
**[16SEP2020 thru 30SEP2020] vs [16SEP2019 thru 30SEP2019]**	1.549	1.364	1.759	< 0.0001
**[01OCT2020 thru 15OCT2020] vs [01OCT2019 thru 15OCT2019]**	1.605	1.421	1.812	< 0.0001
**[16OCT2020 thru 31OCT2020] vs [16OCT2019 thru 31OCT2019]**	1.642	1.468	1.838	< 0.0001
**[01NOV2020 thru 15NOV2020] vs [01NOV2019 thru 15NOV2019]**	1.662	1.490	1.854	< 0.0001
**[16NOV2020 thru 30NOV2020] vs [16NOV2019 thru 30NOV2019]**	1.659	1.478	1.863	< 0.0001
**[01DEC2020 thru 15DEC2020] vs [01DEC2019 thru 15DEC2019]**	1.632	1.441	1.848	< 0.0001
**[16DEC2020 thru 31DEC2020] vs [16DEC2019 thru 31DEC2019]**	1.579	1.392	1.791	< 0.0001
**[01JAN2021 thru 15JAN2021] vs [01JAN2020 thru 15JAN2020]**	1.502	1.330	1.696	< 0.0001
**[16JAN2021 thru 31JAN2021] vs [16JAN2020 thru 31JAN2020]**	1.414	1.251	1.598	< 0.0001
**[01FEB2021 thru 15FEB2021] vs [01FEB2020 thru 15FEB2020]**	1.326	1.165	1.509	0.0001
**[16FEB2021 thru 28FEB2021] vs [16FEB2020 thru 29FEB2020]**	1.247	1.094	1.423	0.0015
**[01MAR2020 thru 31MAR2021] vs [01FEB2019 thru 29FEB2020]**	1.323	1.252	1.397	< 0.0001

Colorado
Bimonthly Data

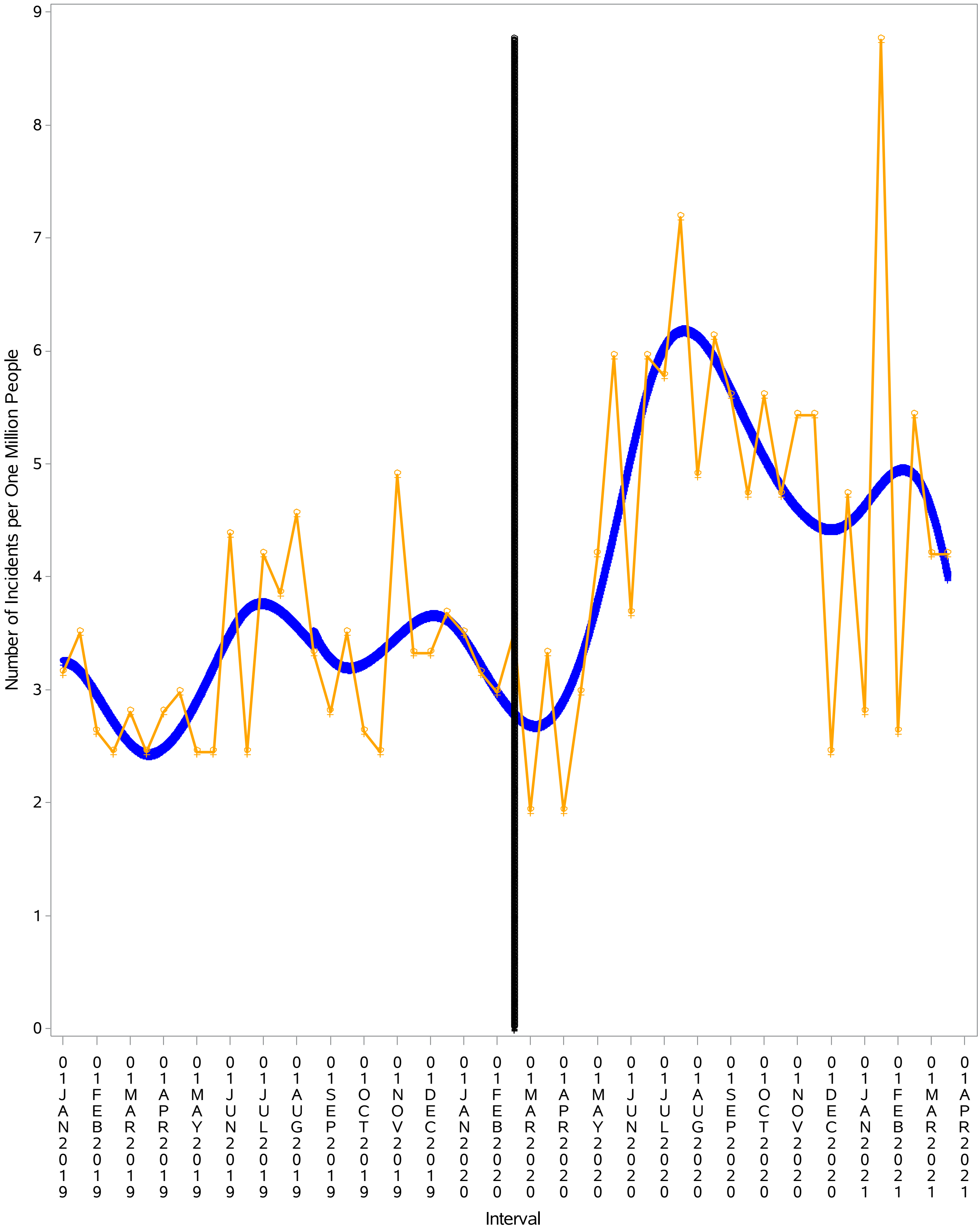

Colorado
Bimonthly Data

Comparison	IntensityRatio	IntensityRatio_LowerCL	IntensityRatio_UpperCL	P_Value
**[01MAR2020 thru 15MAR2020] vs [01MAR2019 thru 15MAR2019]**	1.065	0.722	1.572	0.7443
**[16MAR2020 thru 31MAR2020] vs [16MAR2019 thru 31MAR2019]**	1.117	0.748	1.667	0.5802
**[01APR2020 thru 15APR2020] vs [01APR2019 thru 15APR2019]**	1.175	0.784	1.760	0.426
**[16APR2020 thru 30APR2020] vs [16APR2019 thru 30APR2019]**	1.237	0.850	1.799	0.2586
**[01MAY2020 thru 15MAY2020] vs [01MAY2019 thru 15MAY2019]**	1.303	0.935	1.816	0.1148
**[16MAY2020 thru 31MAY2020] vs [16MAY2019 thru 31MAY2019]**	1.374	1.010	1.868	0.0435
**[01JUN2020 thru 15JUN2020] vs [01JUN2019 thru 15JUN2019]**	1.447	1.057	1.981	0.0222
**[16JUN2020 thru 30JUN2020] vs [16JUN2019 thru 30JUN2019]**	1.524	1.099	2.113	0.0128
**[01JUL2020 thru 15JUL2020] vs [01JUL2019 thru 15JUL2019]**	1.601	1.167	2.197	0.0045
**[16JUL2020 thru 31JUL2020] vs [16JUL2019 thru 31JUL2019]**	1.671	1.250	2.235	0.0009
**[01AUG2020 thru 15AUG2020] vs [01AUG2019 thru 15AUG2019]**	1.724	1.308	2.272	0.0003
**[16AUG2020 thru 31AUG2020] vs [16AUG2019 thru 31AUG2019]**	1.748	1.310	2.332	0.0003
**[01SEP2020 thru 15SEP2020] vs [01SEP2019 thru 15SEP2019]**	1.725	1.262	2.359	0.0011
**[16SEP2020 thru 30SEP2020] vs [16SEP2019 thru 30SEP2019]**	1.671	1.201	2.325	0.0031
**[01OCT2020 thru 15OCT2020] vs [01OCT2019 thru 15OCT2019]**	1.563	1.138	2.145	0.0069
**[16OCT2020 thru 31OCT2020] vs [16OCT2019 thru 31OCT2019]**	1.440	1.071	1.936	0.0169
**[01NOV2020 thru 15NOV2020] vs [01NOV2019 thru 15NOV2019]**	1.327	0.992	1.776	0.0565
**[16NOV2020 thru 30NOV2020] vs [16NOV2019 thru 30NOV2019]**	1.245	0.913	1.698	0.1618
**[01DEC2020 thru 15DEC2020] vs [01DEC2019 thru 15DEC2019]**	1.209	0.866	1.688	0.2581
**[16DEC2020 thru 31DEC2020] vs [16DEC2019 thru 31DEC2019]**	1.236	0.882	1.732	0.2126
**[01JAN2021 thru 15JAN2021] vs [01JAN2020 thru 15JAN2020]**	1.340	0.972	1.849	0.0732
**[16JAN2021 thru 31JAN2021] vs [16JAN2020 thru 31JAN2020]**	1.498	1.087	2.066	0.0148
**[01FEB2021 thru 15FEB2021] vs [01FEB2020 thru 15FEB2020]**	1.662	1.184	2.332	0.0042
**[16FEB2021 thru 28FEB2021] vs [16FEB2020 thru 29FEB2020]**	1.758	1.243	2.487	0.0021
**[01MAR2020 thru 31MAR2021] vs [01FEB2019 thru 29FEB2020]**	1.428	1.234	1.653	< 0.0001

Connecticut
Bimonthly Data

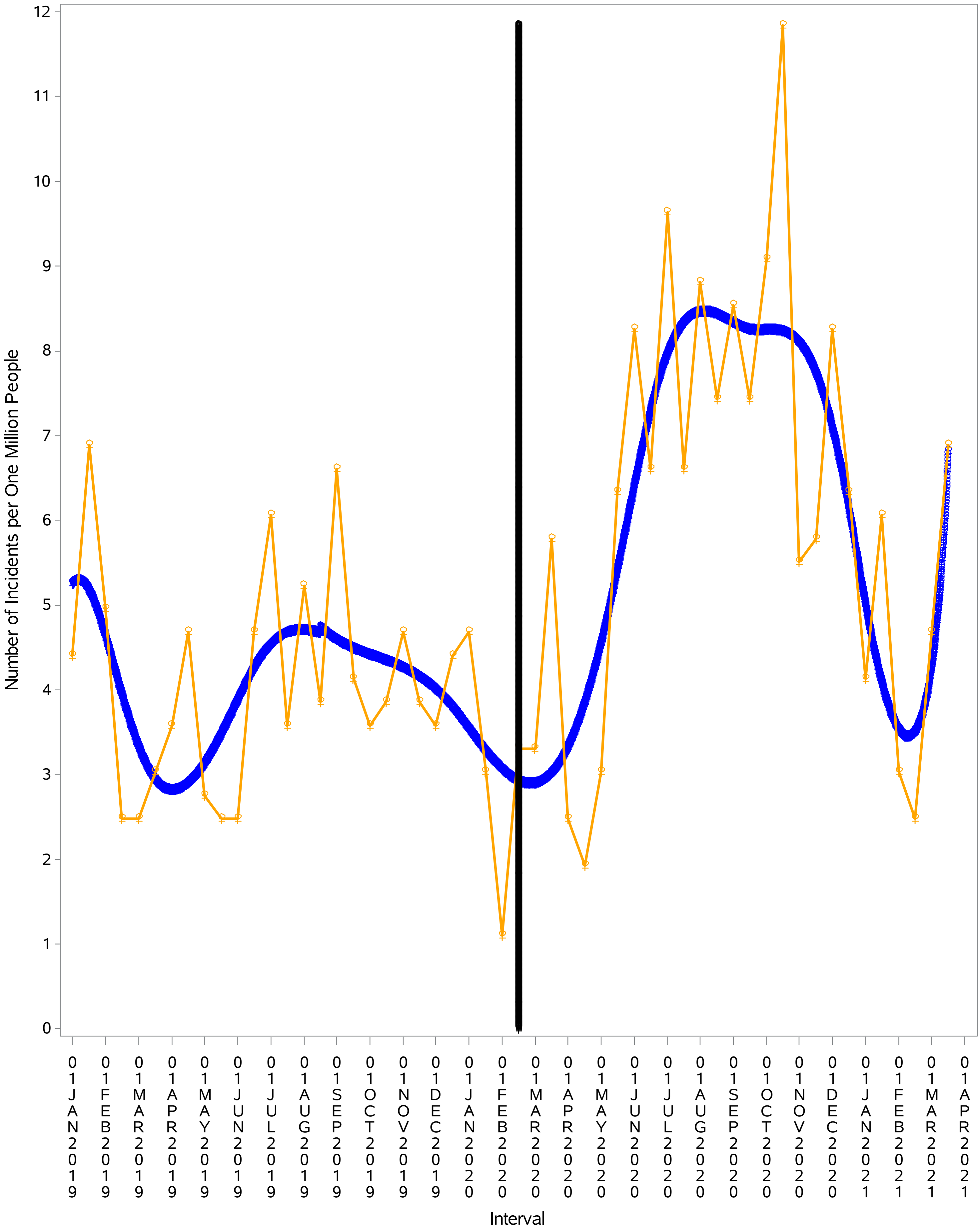

Connecticut
Bimonthly Data

Comparison	IntensityRatio	IntensityRatio_LowerCL	IntensityRatio_UpperCL	P_Value
**[01MAR2020 thru 15MAR2020] vs [01MAR2019 thru 15MAR2019]**	0.873	0.560	1.362	0.5412
**[16MAR2020 thru 31MAR2020] vs [16MAR2019 thru 31MAR2019]**	1.027	0.641	1.645	0.9091
**[01APR2020 thru 15APR2020] vs [01APR2019 thru 15APR2019]**	1.182	0.733	1.907	0.4834
**[16APR2020 thru 30APR2020] vs [16APR2019 thru 30APR2019]**	1.327	0.852	2.065	0.2043
**[01MAY2020 thru 15MAY2020] vs [01MAY2019 thru 15MAY2019]**	1.455	0.985	2.150	0.0594
**[16MAY2020 thru 31MAY2020] vs [16MAY2019 thru 31MAY2019]**	1.563	1.092	2.238	0.0159
**[01JUN2020 thru 15JUN2020] vs [01JUN2019 thru 15JUN2019]**	1.649	1.148	2.369	0.008
**[16JUN2020 thru 30JUN2020] vs [16JUN2019 thru 30JUN2019]**	1.713	1.178	2.491	0.0059
**[01JUL2020 thru 15JUL2020] vs [01JUL2019 thru 15JUL2019]**	1.755	1.224	2.517	0.003
**[16JUL2020 thru 31JUL2020] vs [16JUL2019 thru 31JUL2019]**	1.781	1.284	2.472	0.0009
**[01AUG2020 thru 15AUG2020] vs [01AUG2019 thru 15AUG2019]**	1.796	1.324	2.437	0.0004
**[16AUG2020 thru 31AUG2020] vs [16AUG2019 thru 31AUG2019]**	1.806	1.321	2.470	0.0004
**[01SEP2020 thru 15SEP2020] vs [01SEP2019 thru 15SEP2019]**	1.814	1.296	2.541	0.0009
**[16SEP2020 thru 30SEP2020] vs [16SEP2019 thru 30SEP2019]**	1.837	1.288	2.620	0.0013
**[01OCT2020 thru 15OCT2020] vs [01OCT2019 thru 15OCT2019]**	1.871	1.332	2.628	0.0006
**[16OCT2020 thru 31OCT2020] vs [16OCT2019 thru 31OCT2019]**	1.893	1.377	2.601	0.0002
**[01NOV2020 thru 15NOV2020] vs [01NOV2019 thru 15NOV2019]**	1.897	1.382	2.604	0.0002
**[16NOV2020 thru 30NOV2020] vs [16NOV2019 thru 30NOV2019]**	1.860	1.319	2.622	0.0007
**[01DEC2020 thru 15DEC2020] vs [01DEC2019 thru 15DEC2019]**	1.767	1.214	2.573	0.0039
**[16DEC2020 thru 31DEC2020] vs [16DEC2019 thru 31DEC2019]**	1.611	1.092	2.377	0.0174
**[01JAN2021 thru 15JAN2021] vs [01JAN2020 thru 15JAN2020]**	1.411	0.956	2.082	0.0816
**[16JAN2021 thru 31JAN2021] vs [16JAN2020 thru 31JAN2020]**	1.239	0.821	1.871	0.2997
**[01FEB2021 thru 15FEB2021] vs [01FEB2020 thru 15FEB2020]**	1.153	0.736	1.805	0.5253
**[16FEB2021 thru 28FEB2021] vs [16FEB2020 thru 29FEB2020]**	1.199	0.761	1.891	0.4249
**[01MAR2020 thru 31MAR2021] vs [01FEB2019 thru 29FEB2020]**	1.499	1.267	1.774	< 0.0001

Delaware
Bimonthly Data

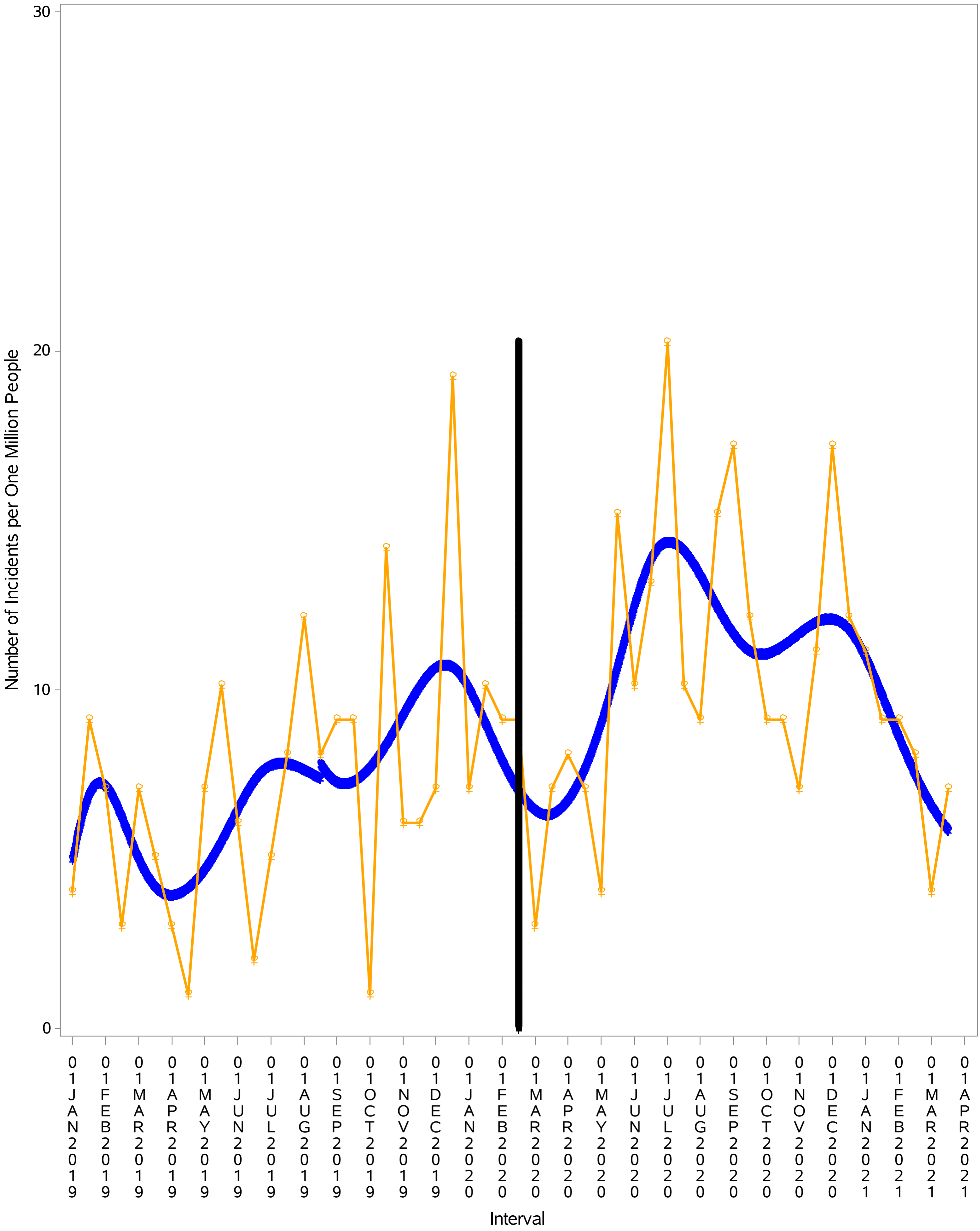

Delaware
Bimonthly Data

Comparison	IntensityRatio	IntensityRatio_LowerCL	IntensityRatio_UpperCL	P_Value
**[01MAR2020 thru 15MAR2020] vs [01MAR2019 thru 15MAR2019]**	1.292	0.690	2.419	0.4151
**[16MAR2020 thru 31MAR2020] vs [16MAR2019 thru 31MAR2019]**	1.516	0.775	2.964	0.2174
**[01APR2020 thru 15APR2020] vs [01APR2019 thru 15APR2019]**	1.711	0.858	3.414	0.1239
**[16APR2020 thru 30APR2020] vs [16APR2019 thru 30APR2019]**	1.847	0.969	3.520	0.0618
**[01MAY2020 thru 15MAY2020] vs [01MAY2019 thru 15MAY2019]**	1.921	1.088	3.391	0.0254
**[16MAY2020 thru 31MAY2020] vs [16MAY2019 thru 31MAY2019]**	1.943	1.162	3.251	0.0126
**[01JUN2020 thru 15JUN2020] vs [01JUN2019 thru 15JUN2019]**	1.928	1.155	3.218	0.0133
**[16JUN2020 thru 30JUN2020] vs [16JUN2019 thru 30JUN2019]**	1.892	1.116	3.209	0.0192
**[01JUL2020 thru 15JUL2020] vs [01JUL2019 thru 15JUL2019]**	1.850	1.109	3.085	0.0196
**[16JUL2020 thru 31JUL2020] vs [16JUL2019 thru 31JUL2019]**	1.802	1.126	2.884	0.0153
**[01AUG2020 thru 15AUG2020] vs [01AUG2019 thru 15AUG2019]**	1.747	1.122	2.722	0.0148
**[16AUG2020 thru 31AUG2020] vs [16AUG2019 thru 31AUG2019]**	1.684	1.066	2.661	0.0266
**[01SEP2020 thru 15SEP2020] vs [01SEP2019 thru 15SEP2019]**	1.600	0.977	2.619	0.0611
**[16SEP2020 thru 30SEP2020] vs [16SEP2019 thru 30SEP2019]**	1.528	0.911	2.565	0.1059
**[01OCT2020 thru 15OCT2020] vs [01OCT2019 thru 15OCT2019]**	1.441	0.880	2.359	0.1428
**[16OCT2020 thru 31OCT2020] vs [16OCT2019 thru 31OCT2019]**	1.345	0.855	2.117	0.1944
**[01NOV2020 thru 15NOV2020] vs [01NOV2019 thru 15NOV2019]**	1.260	0.815	1.949	0.2898
**[16NOV2020 thru 30NOV2020] vs [16NOV2019 thru 30NOV2019]**	1.189	0.754	1.874	0.4469
**[01DEC2020 thru 15DEC2020] vs [01DEC2019 thru 15DEC2019]**	1.135	0.699	1.842	0.6001
**[16DEC2020 thru 31DEC2020] vs [16DEC2019 thru 31DEC2019]**	1.103	0.676	1.799	0.6886
**[01JAN2021 thru 15JAN2021] vs [01JAN2020 thru 15JAN2020]**	1.093	0.676	1.765	0.7107
**[16JAN2021 thru 31JAN2021] vs [16JAN2020 thru 31JAN2020]**	1.093	0.657	1.820	0.7252
**[01FEB2021 thru 15FEB2021] vs [01FEB2020 thru 15FEB2020]**	1.090	0.622	1.911	0.7582
**[16FEB2021 thru 28FEB2021] vs [16FEB2020 thru 29FEB2020]**	1.068	0.596	1.914	0.8199
**[01MAR2020 thru 31MAR2021] vs [01FEB2019 thru 29FEB2020]**	1.403	1.109	1.774	0.0058

District of Columbia
Bimonthly Data

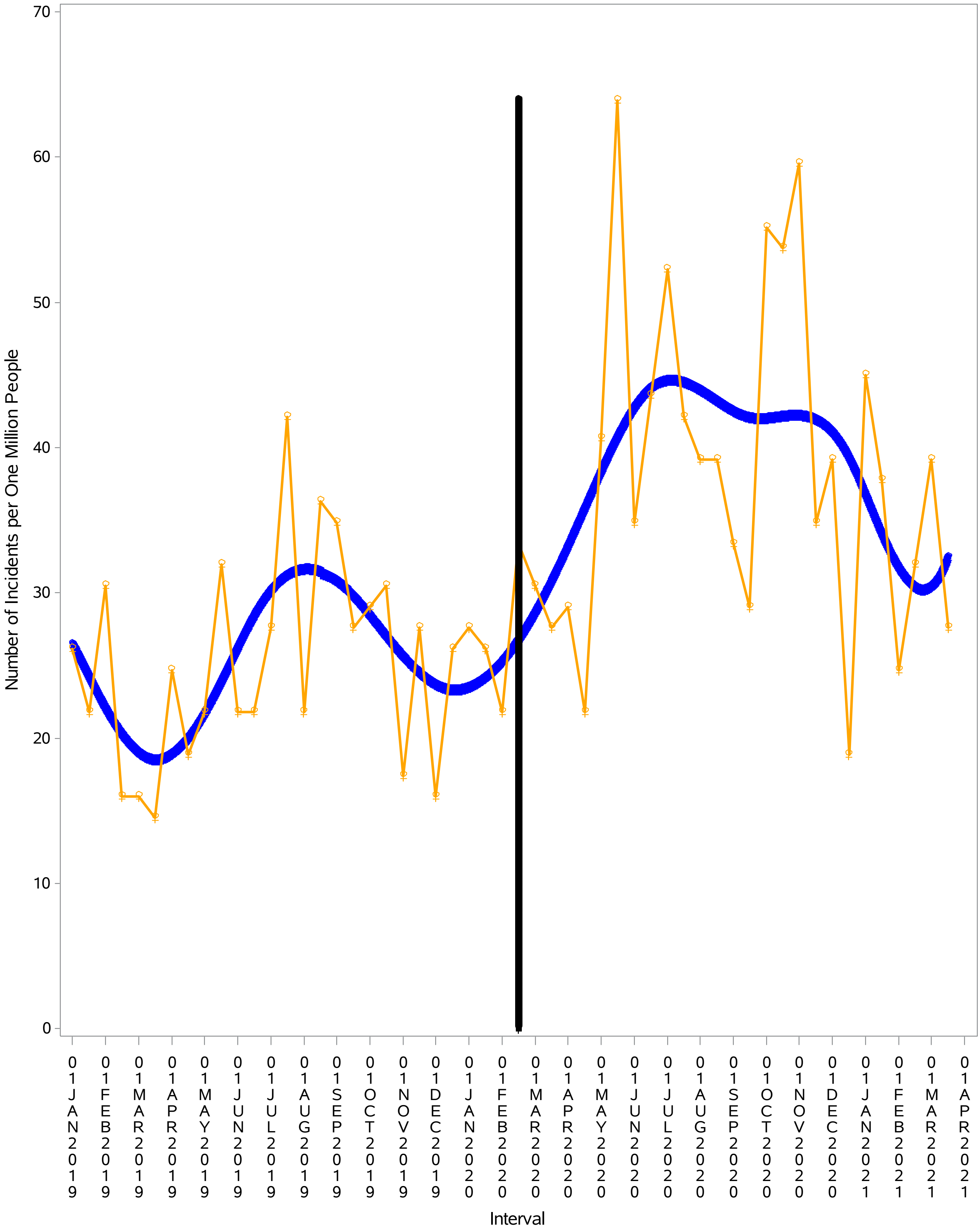

District of Columbia
Bimonthly Data

Comparison	IntensityRatio	IntensityRatio_LowerCL	IntensityRatio_UpperCL	P_Value
**[01MAR2020 thru 15MAR2020] vs [01MAR2019 thru 15MAR2019]**	1.507	1.027	2.210	0.0366
**[16MAR2020 thru 31MAR2020] vs [16MAR2019 thru 31MAR2019]**	1.664	1.124	2.465	0.0123
**[01APR2020 thru 15APR2020] vs [01APR2019 thru 15APR2019]**	1.760	1.181	2.623	0.0066
**[16APR2020 thru 30APR2020] vs [16APR2019 thru 30APR2019]**	1.791	1.234	2.598	0.0029
**[01MAY2020 thru 15MAY2020] vs [01MAY2019 thru 15MAY2019]**	1.767	1.269	2.462	0.0012
**[16MAY2020 thru 31MAY2020] vs [16MAY2019 thru 31MAY2019]**	1.708	1.253	2.327	0.0011
**[01JUN2020 thru 15JUN2020] vs [01JUN2019 thru 15JUN2019]**	1.630	1.187	2.239	0.0034
**[16JUN2020 thru 30JUN2020] vs [16JUN2019 thru 30JUN2019]**	1.550	1.113	2.159	0.0107
**[01JUL2020 thru 15JUL2020] vs [01JUL2019 thru 15JUL2019]**	1.481	1.075	2.041	0.0175
**[16JUL2020 thru 31JUL2020] vs [16JUL2019 thru 31JUL2019]**	1.427	1.063	1.914	0.019
**[01AUG2020 thru 15AUG2020] vs [01AUG2019 thru 15AUG2019]**	1.390	1.054	1.833	0.021
**[16AUG2020 thru 31AUG2020] vs [16AUG2019 thru 31AUG2019]**	1.373	1.031	1.829	0.0311
**[01SEP2020 thru 15SEP2020] vs [01SEP2019 thru 15SEP2019]**	1.380	1.012	1.882	0.0419
**[16SEP2020 thru 30SEP2020] vs [16SEP2019 thru 30SEP2019]**	1.414	1.020	1.961	0.0383
**[01OCT2020 thru 15OCT2020] vs [01OCT2019 thru 15OCT2019]**	1.481	1.082	2.026	0.0154
**[16OCT2020 thru 31OCT2020] vs [16OCT2019 thru 31OCT2019]**	1.563	1.165	2.098	0.0038
**[01NOV2020 thru 15NOV2020] vs [01NOV2019 thru 15NOV2019]**	1.648	1.230	2.208	0.0013
**[16NOV2020 thru 30NOV2020] vs [16NOV2019 thru 30NOV2019]**	1.713	1.250	2.347	0.0013
**[01DEC2020 thru 15DEC2020] vs [01DEC2019 thru 15DEC2019]**	1.733	1.232	2.436	0.0022
**[16DEC2020 thru 31DEC2020] vs [16DEC2019 thru 31DEC2019]**	1.684	1.190	2.383	0.0042
**[01JAN2021 thru 15JAN2021] vs [01JAN2020 thru 15JAN2020]**	1.563	1.117	2.188	0.0104
**[16JAN2021 thru 31JAN2021] vs [16JAN2020 thru 31JAN2020]**	1.407	1.001	1.979	0.0494
**[01FEB2021 thru 15FEB2021] vs [01FEB2020 thru 15FEB2020]**	1.256	0.877	1.799	0.2073
**[16FEB2021 thru 28FEB2021] vs [16FEB2020 thru 29FEB2020]**	1.136	0.792	1.627	0.4798
**[01MAR2020 thru 31MAR2021] vs [01FEB2019 thru 29FEB2020]**	1.528	1.315	1.775	< 0.0001

Florida
Bimonthly Data

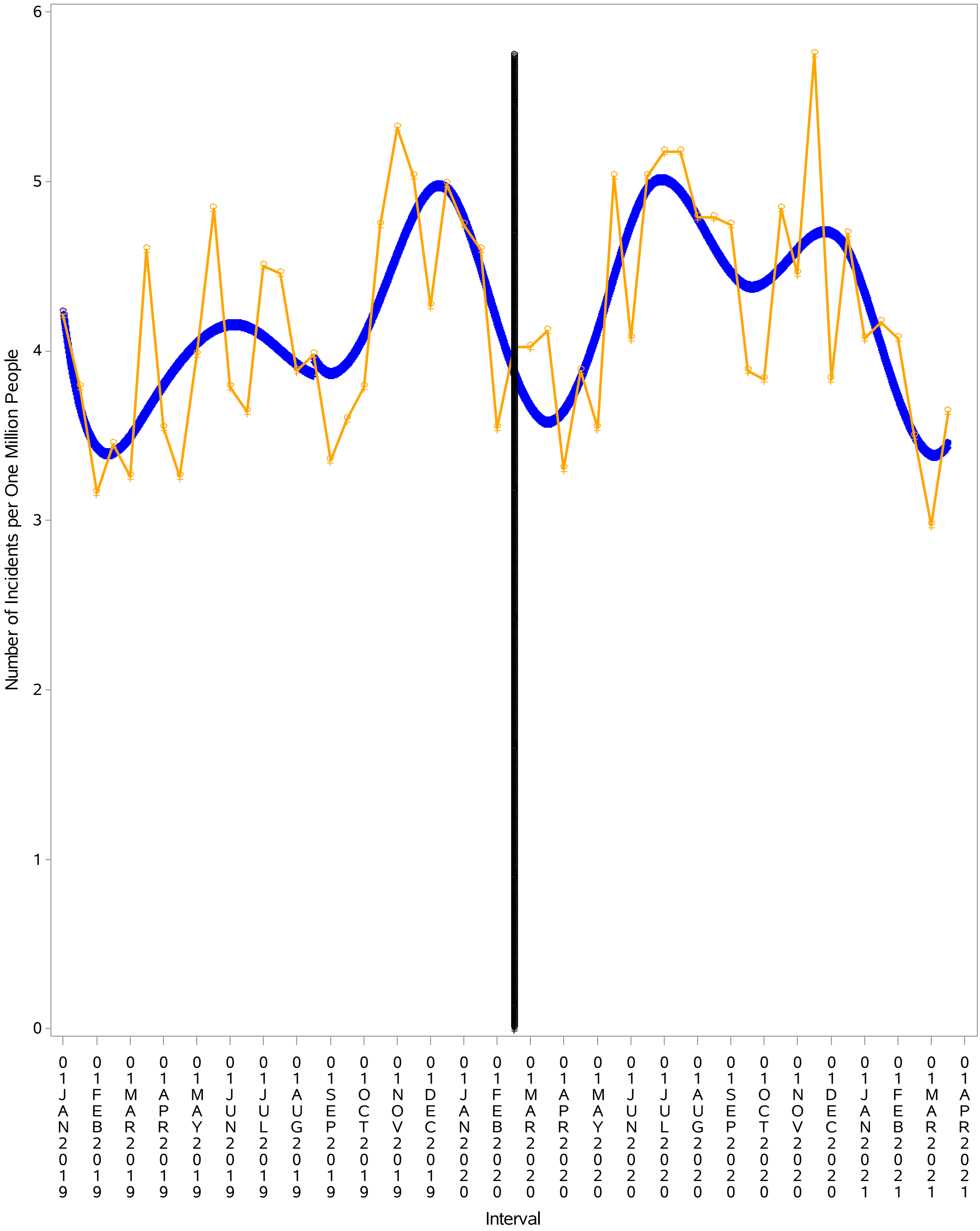

Florida
Bimonthly Data

Comparison	IntensityRatio	IntensityRatio_LowerCL	IntensityRatio_UpperCL	P_Value
**[01MAR2020 thru 15MAR2020] vs [01MAR2019 thru 15MAR2019]**	1.049	0.898	1.225	0.5377
**[16MAR2020 thru 31MAR2020] vs [16MAR2019 thru 31MAR2019]**	0.982	0.839	1.149	0.8183
**[01APR2020 thru 15APR2020] vs [01APR2019 thru 15APR2019]**	0.960	0.820	1.125	0.6089
**[16APR2020 thru 30APR2020] vs [16APR2019 thru 30APR2019]**	0.976	0.842	1.132	0.7459
**[01MAY2020 thru 15MAY2020] vs [01MAY2019 thru 15MAY2019]**	1.019	0.891	1.166	0.7779
**[16MAY2020 thru 31MAY2020] vs [16MAY2019 thru 31MAY2019]**	1.078	0.946	1.229	0.252
**[01JUN2020 thru 15JUN2020] vs [01JUN2019 thru 15JUN2019]**	1.142	0.995	1.312	0.0594
**[16JUN2020 thru 30JUN2020] vs [16JUN2019 thru 30JUN2019]**	1.196	1.034	1.384	0.0171
**[01JUL2020 thru 15JUL2020] vs [01JUL2019 thru 15JUL2019]**	1.227	1.065	1.413	0.0057
**[16JUL2020 thru 31JUL2020] vs [16JUL2019 thru 31JUL2019]**	1.233	1.082	1.406	0.0024
**[01AUG2020 thru 15AUG2020] vs [01AUG2019 thru 15AUG2019]**	1.220	1.076	1.383	0.0026
**[16AUG2020 thru 31AUG2020] vs [16AUG2019 thru 31AUG2019]**	1.192	1.046	1.359	0.0097
**[01SEP2020 thru 15SEP2020] vs [01SEP2019 thru 15SEP2019]**	1.153	1.001	1.327	0.0486
**[16SEP2020 thru 30SEP2020] vs [16SEP2019 thru 30SEP2019]**	1.116	0.963	1.293	0.1391
**[01OCT2020 thru 15OCT2020] vs [01OCT2019 thru 15OCT2019]**	1.079	0.939	1.241	0.2767
**[16OCT2020 thru 31OCT2020] vs [16OCT2019 thru 31OCT2019]**	1.040	0.915	1.184	0.5377
**[01NOV2020 thru 15NOV2020] vs [01NOV2019 thru 15NOV2019]**	1.006	0.888	1.141	0.9191
**[16NOV2020 thru 30NOV2020] vs [16NOV2019 thru 30NOV2019]**	0.976	0.854	1.114	0.71
**[01DEC2020 thru 15DEC2020] vs [01DEC2019 thru 15DEC2019]**	0.949	0.823	1.094	0.4587
**[16DEC2020 thru 31DEC2020] vs [16DEC2019 thru 31DEC2019]**	0.926	0.803	1.068	0.2839
**[01JAN2021 thru 15JAN2021] vs [01JAN2020 thru 15JAN2020]**	0.908	0.791	1.043	0.1671
**[16JAN2021 thru 31JAN2021] vs [16JAN2020 thru 31JAN2020]**	0.897	0.776	1.037	0.1381
**[01FEB2021 thru 15FEB2021] vs [01FEB2020 thru 15FEB2020]**	0.895	0.763	1.049	0.1663
**[16FEB2021 thru 28FEB2021] vs [16FEB2020 thru 29FEB2020]**	0.903	0.766	1.064	0.2167
**[01MAR2020 thru 31MAR2021] vs [01FEB2019 thru 29FEB2020]**	1.038	0.975	1.106	0.2372

Georgia
Bimonthly Data

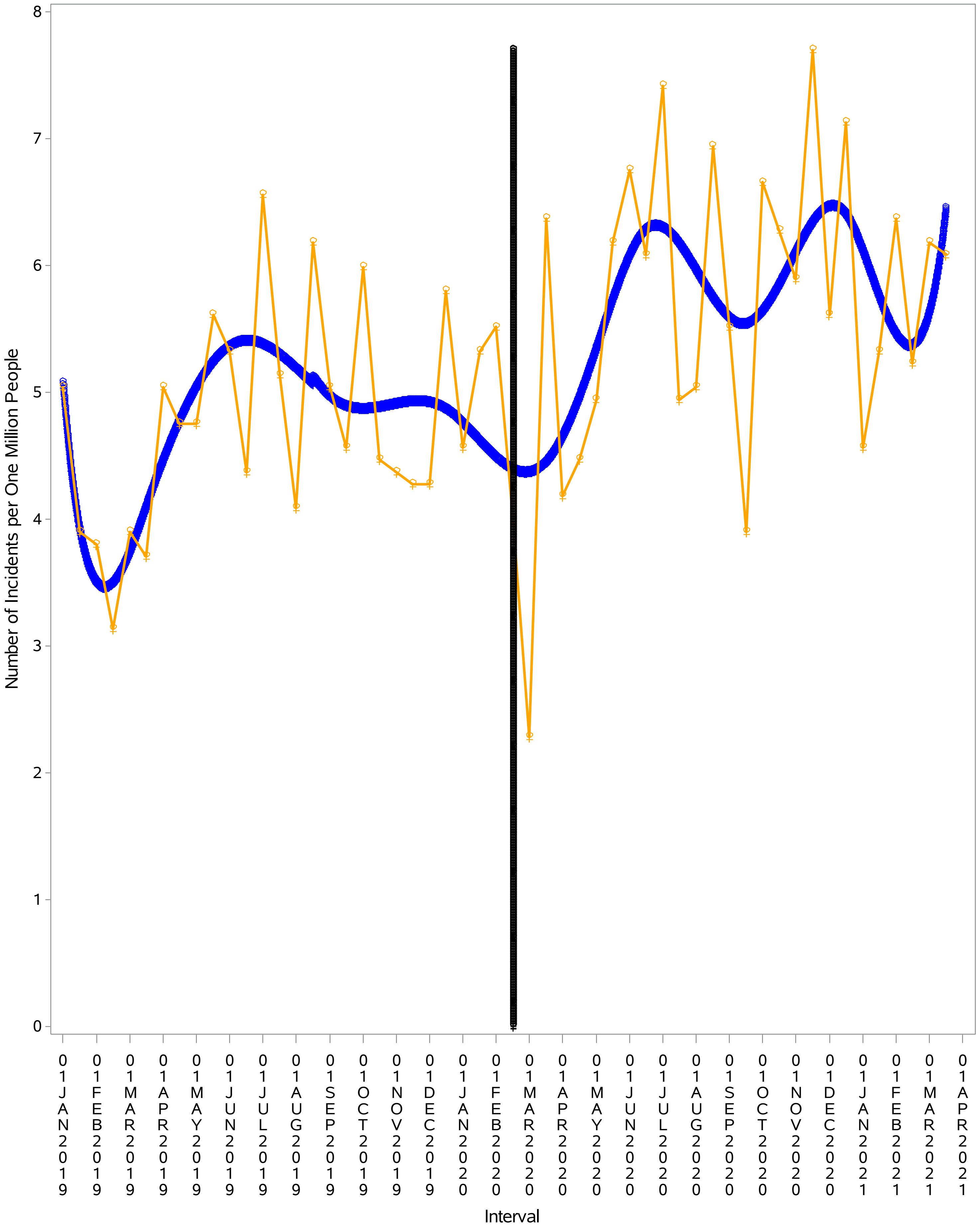

Georgia
Bimonthly Data

Comparison	IntensityRatio	IntensityRatio_LowerCL	IntensityRatio_UpperCL	P_Value
**[01MAR2020 thru 15MAR2020] vs [01MAR2019 thru 15MAR2019]**	1.169	0.909	1.502	0.217
**[16MAR2020 thru 31MAR2020] vs [16MAR2019 thru 31MAR2019]**	1.085	0.845	1.391	0.5143
**[01APR2020 thru 15APR2020] vs [01APR2019 thru 15APR2019]**	1.043	0.814	1.337	0.733
**[16APR2020 thru 30APR2020] vs [16APR2019 thru 30APR2019]**	1.039	0.825	1.309	0.7401
**[01MAY2020 thru 15MAY2020] vs [01MAY2019 thru 15MAY2019]**	1.060	0.860	1.306	0.5783
**[16MAY2020 thru 31MAY2020] vs [16MAY2019 thru 31MAY2019]**	1.095	0.896	1.338	0.3683
**[01JUN2020 thru 15JUN2020] vs [01JUN2019 thru 15JUN2019]**	1.133	0.917	1.399	0.2396
**[16JUN2020 thru 30JUN2020] vs [16JUN2019 thru 30JUN2019]**	1.162	0.930	1.451	0.1803
**[01JUL2020 thru 15JUL2020] vs [01JUL2019 thru 15JUL2019]**	1.171	0.944	1.453	0.1477
**[16JUL2020 thru 31JUL2020] vs [16JUL2019 thru 31JUL2019]**	1.164	0.953	1.421	0.1325
**[01AUG2020 thru 15AUG2020] vs [01AUG2019 thru 15AUG2019]**	1.149	0.949	1.392	0.1505
**[16AUG2020 thru 31AUG2020] vs [16AUG2019 thru 31AUG2019]**	1.134	0.928	1.386	0.2136
**[01SEP2020 thru 15SEP2020] vs [01SEP2019 thru 15SEP2019]**	1.124	0.905	1.397	0.2832
**[16SEP2020 thru 30SEP2020] vs [16SEP2019 thru 30SEP2019]**	1.132	0.901	1.422	0.2778
**[01OCT2020 thru 15OCT2020] vs [01OCT2019 thru 15OCT2019]**	1.160	0.934	1.441	0.1734
**[16OCT2020 thru 31OCT2020] vs [16OCT2019 thru 31OCT2019]**	1.198	0.980	1.464	0.0761
**[01NOV2020 thru 15NOV2020] vs [01NOV2019 thru 15NOV2019]**	1.244	1.023	1.513	0.0296
**[16NOV2020 thru 30NOV2020] vs [16NOV2019 thru 30NOV2019]**	1.286	1.045	1.583	0.0188
**[01DEC2020 thru 15DEC2020] vs [01DEC2019 thru 15DEC2019]**	1.313	1.050	1.642	0.0179
**[16DEC2020 thru 31DEC2020] vs [16DEC2019 thru 31DEC2019]**	1.314	1.049	1.648	0.0189
**[01JAN2021 thru 15JAN2021] vs [01JAN2020 thru 15JAN2020]**	1.285	1.034	1.596	0.0245
**[16JAN2021 thru 31JAN2021] vs [16JAN2020 thru 31JAN2020]**	1.244	0.998	1.551	0.0517
**[01FEB2021 thru 15FEB2021] vs [01FEB2020 thru 15FEB2020]**	1.216	0.961	1.540	0.1015
**[16FEB2021 thru 28FEB2021] vs [16FEB2020 thru 29FEB2020]**	1.222	0.961	1.555	0.0995
**[01MAR2020 thru 31MAR2021] vs [01FEB2019 thru 29FEB2020]**	1.209	1.095	1.333	0.0004

Hawaii
Bimonthly Data

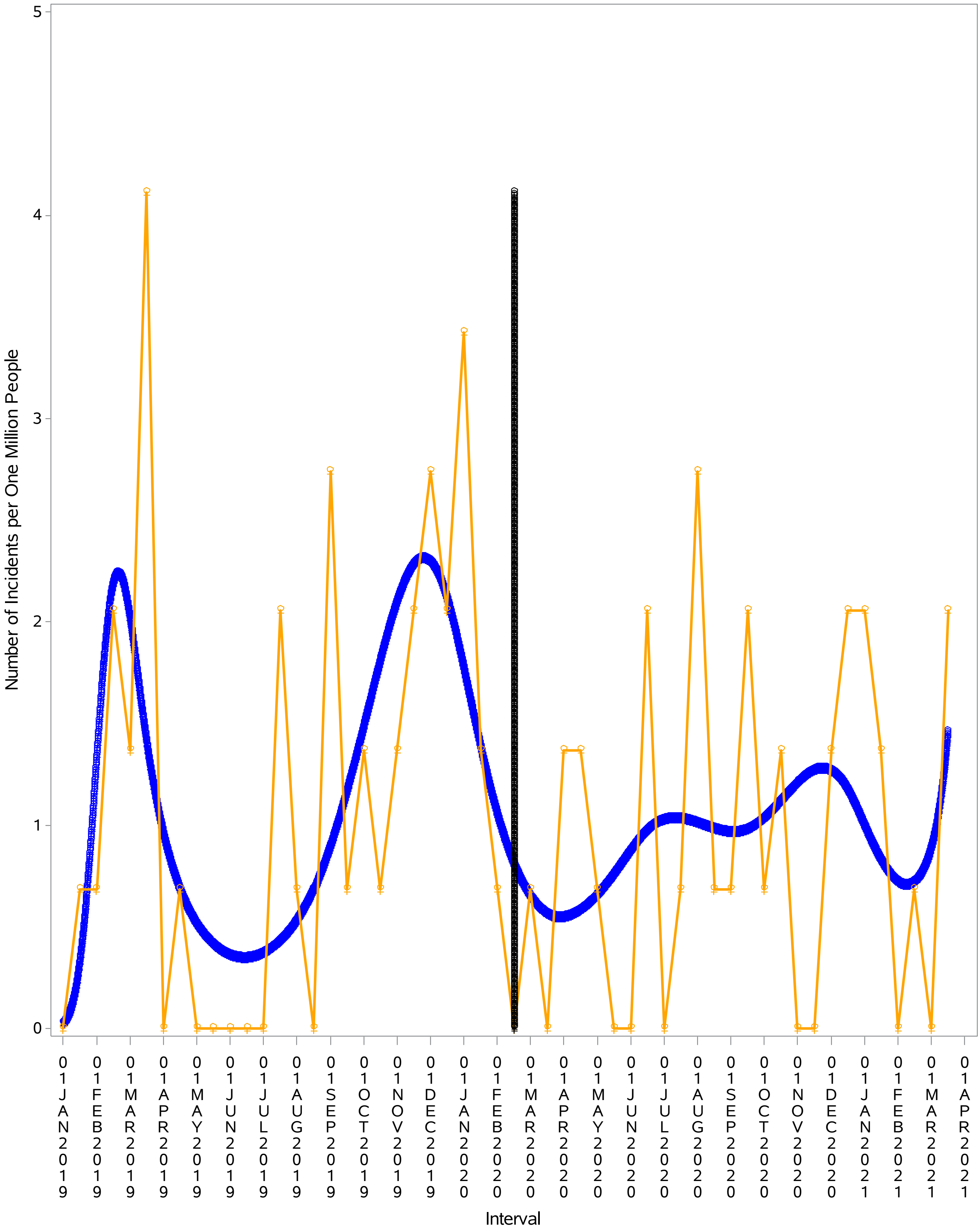

Hawaii
Bimonthly Data

Comparison	IntensityRatio	IntensityRatio_LowerCL	IntensityRatio_UpperCL	P_Value
**[01MAR2020 thru 15MAR2020] vs [01MAR2019 thru 15MAR2019]**	0.323	0.090	1.166	0.083
**[16MAR2020 thru 31MAR2020] vs [16MAR2019 thru 31MAR2019]**	0.405	0.102	1.600	0.1914
**[01APR2020 thru 15APR2020] vs [01APR2019 thru 15APR2019]**	0.576	0.134	2.472	0.4486
**[16APR2020 thru 30APR2020] vs [16APR2019 thru 30APR2019]**	0.857	0.205	3.582	0.8282
**[01MAY2020 thru 15MAY2020] vs [01MAY2019 thru 15MAY2019]**	1.278	0.319	5.112	0.7229
**[16MAY2020 thru 31MAY2020] vs [16MAY2019 thru 31MAY2019]**	1.830	0.438	7.640	0.3986
**[01JUN2020 thru 15JUN2020] vs [01JUN2019 thru 15JUN2019]**	2.407	0.509	11.376	0.2602
**[16JUN2020 thru 30JUN2020] vs [16JUN2019 thru 30JUN2019]**	2.786	0.539	14.394	0.2149
**[01JUL2020 thru 15JUL2020] vs [01JUL2019 thru 15JUL2019]**	2.755	0.561	13.524	0.2057
**[16JUL2020 thru 31JUL2020] vs [16JUL2019 thru 31JUL2019]**	2.391	0.569	10.052	0.2273
**[01AUG2020 thru 15AUG2020] vs [01AUG2019 thru 15AUG2019]**	1.899	0.531	6.795	0.316
**[16AUG2020 thru 31AUG2020] vs [16AUG2019 thru 31AUG2019]**	1.437	0.434	4.756	0.5445
**[01SEP2020 thru 15SEP2020] vs [01SEP2019 thru 15SEP2019]**	1.082	0.325	3.596	0.8959
**[16SEP2020 thru 30SEP2020] vs [16SEP2019 thru 30SEP2019]**	0.840	0.250	2.818	0.7726
**[01OCT2020 thru 15OCT2020] vs [01OCT2019 thru 15OCT2019]**	0.701	0.228	2.159	0.5275
**[16OCT2020 thru 31OCT2020] vs [16OCT2019 thru 31OCT2019]**	0.618	0.225	1.702	0.3434
**[01NOV2020 thru 15NOV2020] vs [01NOV2019 thru 15NOV2019]**	0.576	0.220	1.506	0.2532
**[16NOV2020 thru 30NOV2020] vs [16NOV2019 thru 30NOV2019]**	0.557	0.203	1.530	0.2491
**[01DEC2020 thru 15DEC2020] vs [01DEC2019 thru 15DEC2019]**	0.553	0.187	1.637	0.2771
**[16DEC2020 thru 31DEC2020] vs [16DEC2019 thru 31DEC2019]**	0.556	0.185	1.674	0.2889
**[01JAN2021 thru 15JAN2021] vs [01JAN2020 thru 15JAN2020]**	0.565	0.189	1.691	0.2992
**[16JAN2021 thru 31JAN2021] vs [16JAN2020 thru 31JAN2020]**	0.598	0.177	2.025	0.3998
**[01FEB2021 thru 15FEB2021] vs [01FEB2020 thru 15FEB2020]**	0.688	0.168	2.821	0.5951
**[16FEB2021 thru 28FEB2021] vs [16FEB2020 thru 29FEB2020]**	0.894	0.201	3.972	0.8802
**[01MAR2020 thru 31MAR2021] vs [01FEB2019 thru 29FEB2020]**	0.883	0.493	1.581	0.6687

Idaho
Bimonthly Data

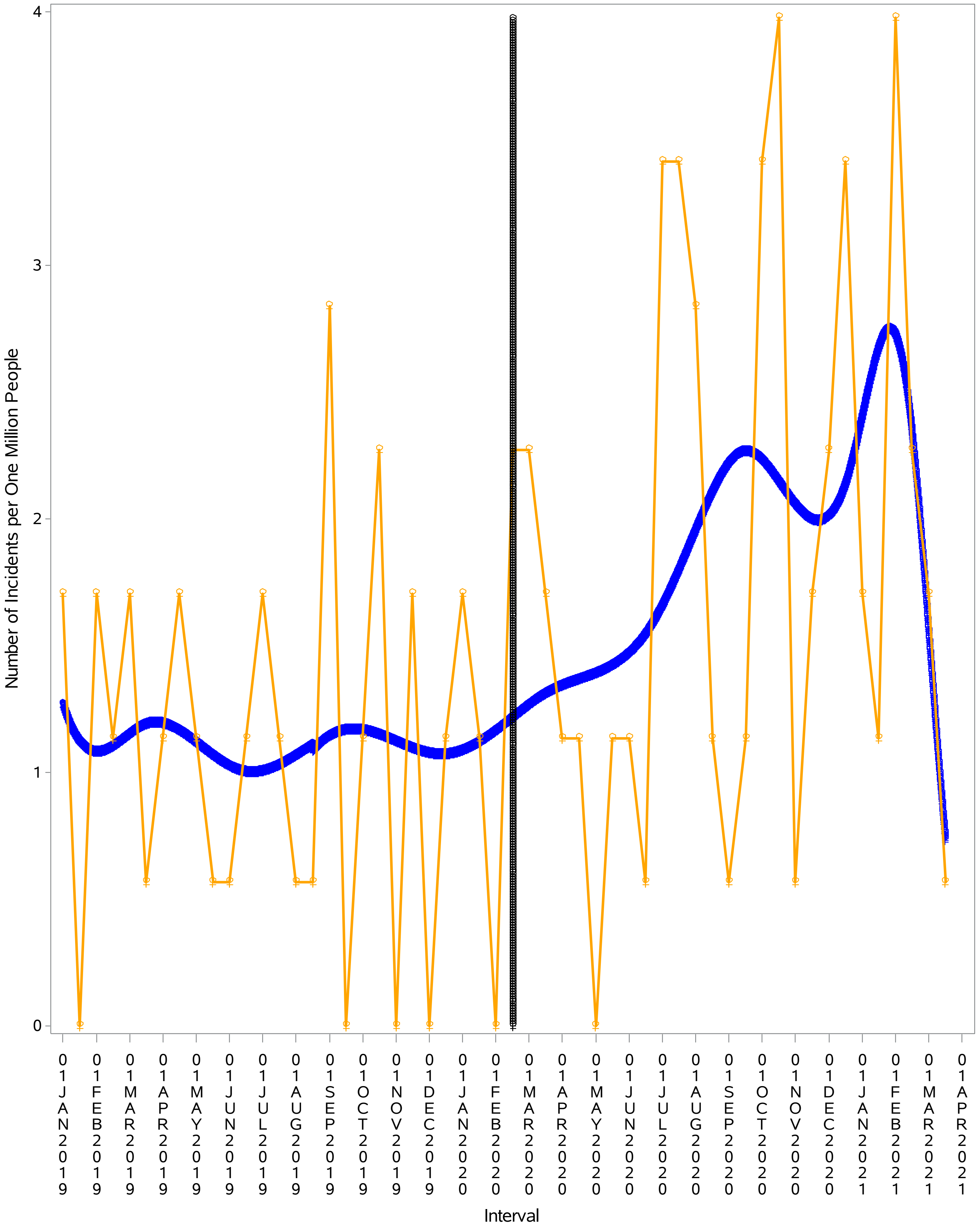

Idaho
Bimonthly Data

Comparison	IntensityRatio	IntensityRatio_LowerCL	IntensityRatio_UpperCL	P_Value
**[01MAR2020 thru 15MAR2020] vs [01MAR2019 thru 15MAR2019]**	1.101	0.428	2.829	0.8385
**[16MAR2020 thru 31MAR2020] vs [16MAR2019 thru 31MAR2019]**	1.101	0.420	2.883	0.8415
**[01APR2020 thru 15APR2020] vs [01APR2019 thru 15APR2019]**	1.125	0.424	2.984	0.8094
**[16APR2020 thru 30APR2020] vs [16APR2019 thru 30APR2019]**	1.173	0.469	2.937	0.7271
**[01MAY2020 thru 15MAY2020] vs [01MAY2019 thru 15MAY2019]**	1.243	0.534	2.896	0.6059
**[16MAY2020 thru 31MAY2020] vs [16MAY2019 thru 31MAY2019]**	1.331	0.577	3.071	0.4937
**[01JUN2020 thru 15JUN2020] vs [01JUN2019 thru 15JUN2019]**	1.432	0.585	3.507	0.423
**[16JUN2020 thru 30JUN2020] vs [16JUN2019 thru 30JUN2019]**	1.540	0.597	3.970	0.3629
**[01JUL2020 thru 15JUL2020] vs [01JUL2019 thru 15JUL2019]**	1.647	0.658	4.124	0.2792
**[16JUL2020 thru 31JUL2020] vs [16JUL2019 thru 31JUL2019]**	1.746	0.757	4.029	0.1856
**[01AUG2020 thru 15AUG2020] vs [01AUG2019 thru 15AUG2019]**	1.832	0.847	3.964	0.1208
**[16AUG2020 thru 31AUG2020] vs [16AUG2019 thru 31AUG2019]**	1.898	0.872	4.128	0.1037
**[01SEP2020 thru 15SEP2020] vs [01SEP2019 thru 15SEP2019]**	1.940	0.850	4.428	0.1125
**[16SEP2020 thru 30SEP2020] vs [16SEP2019 thru 30SEP2019]**	1.939	0.816	4.606	0.1301
**[01OCT2020 thru 15OCT2020] vs [01OCT2019 thru 15OCT2019]**	1.902	0.831	4.353	0.1245
**[16OCT2020 thru 31OCT2020] vs [16OCT2019 thru 31OCT2019]**	1.864	0.863	4.023	0.1101
**[01NOV2020 thru 15NOV2020] vs [01NOV2019 thru 15NOV2019]**	1.827	0.861	3.880	0.1137
**[16NOV2020 thru 30NOV2020] vs [16NOV2019 thru 30NOV2019]**	1.821	0.818	4.052	0.1381
**[01DEC2020 thru 15DEC2020] vs [01DEC2019 thru 15DEC2019]**	1.866	0.789	4.417	0.1512
**[16DEC2020 thru 31DEC2020] vs [16DEC2019 thru 31DEC2019]**	1.992	0.832	4.767	0.1186
**[01JAN2021 thru 15JAN2021] vs [01JAN2020 thru 15JAN2020]**	2.207	0.966	5.044	0.06
**[16JAN2021 thru 31JAN2021] vs [16JAN2020 thru 31JAN2020]**	2.388	1.071	5.323	0.034
**[01FEB2021 thru 15FEB2021] vs [01FEB2020 thru 15FEB2020]**	2.337	1.038	5.265	0.0409
**[16FEB2021 thru 28FEB2021] vs [16FEB2020 thru 29FEB2020]**	1.917	0.841	4.373	0.1185
**[01MAR2020 thru 31MAR2021] vs [01FEB2019 thru 29FEB2020]**	1.608	1.088	2.375	0.0183

Illinois
Bimonthly Data

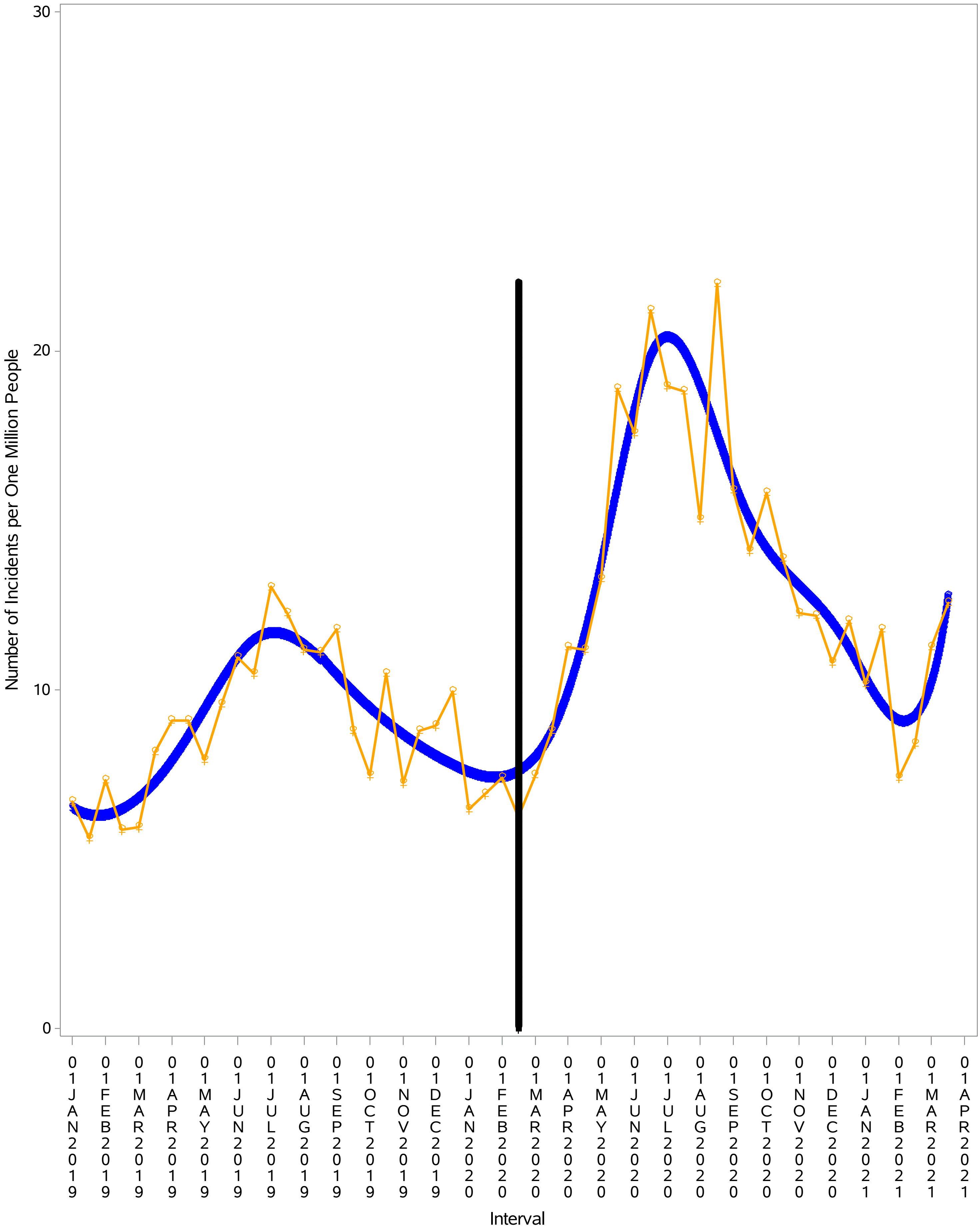

Illinois
Bimonthly Data

Comparison	IntensityRatio	IntensityRatio_LowerCL	IntensityRatio_UpperCL	P_Value
**[01MAR2020 thru 15MAR2020] vs [01MAR2019 thru 15MAR2019]**	1.178	0.967	1.434	0.1019
**[16MAR2020 thru 31MAR2020] vs [16MAR2019 thru 31MAR2019]**	1.204	0.992	1.461	0.0599
**[01APR2020 thru 15APR2020] vs [01APR2019 thru 15APR2019]**	1.263	1.041	1.531	0.019
**[16APR2020 thru 30APR2020] vs [16APR2019 thru 30APR2019]**	1.350	1.129	1.613	0.0015
**[01MAY2020 thru 15MAY2020] vs [01MAY2019 thru 15MAY2019]**	1.455	1.244	1.702	< 0.0001
**[16MAY2020 thru 31MAY2020] vs [16MAY2019 thru 31MAY2019]**	1.566	1.356	1.809	< 0.0001
**[01JUN2020 thru 15JUN2020] vs [01JUN2019 thru 15JUN2019]**	1.665	1.437	1.929	< 0.0001
**[16JUN2020 thru 30JUN2020] vs [16JUN2019 thru 30JUN2019]**	1.731	1.484	2.019	< 0.0001
**[01JUL2020 thru 15JUL2020] vs [01JUL2019 thru 15JUL2019]**	1.747	1.505	2.029	< 0.0001
**[16JUL2020 thru 31JUL2020] vs [16JUL2019 thru 31JUL2019]**	1.721	1.499	1.976	< 0.0001
**[01AUG2020 thru 15AUG2020] vs [01AUG2019 thru 15AUG2019]**	1.669	1.462	1.906	< 0.0001
**[16AUG2020 thru 31AUG2020] vs [16AUG2019 thru 31AUG2019]**	1.608	1.397	1.850	< 0.0001
**[01SEP2020 thru 15SEP2020] vs [01SEP2019 thru 15SEP2019]**	1.550	1.329	1.807	< 0.0001
**[16SEP2020 thru 30SEP2020] vs [16SEP2019 thru 30SEP2019]**	1.511	1.284	1.778	< 0.0001
**[01OCT2020 thru 15OCT2020] vs [01OCT2019 thru 15OCT2019]**	1.498	1.281	1.753	< 0.0001
**[16OCT2020 thru 31OCT2020] vs [16OCT2019 thru 31OCT2019]**	1.498	1.290	1.739	< 0.0001
**[01NOV2020 thru 15NOV2020] vs [01NOV2019 thru 15NOV2019]**	1.504	1.294	1.749	< 0.0001
**[16NOV2020 thru 30NOV2020] vs [16NOV2019 thru 30NOV2019]**	1.504	1.276	1.773	< 0.0001
**[01DEC2020 thru 15DEC2020] vs [01DEC2019 thru 15DEC2019]**	1.486	1.243	1.776	0.0001
**[16DEC2020 thru 31DEC2020] vs [16DEC2019 thru 31DEC2019]**	1.439	1.199	1.726	0.0002
**[01JAN2021 thru 15JAN2021] vs [01JAN2020 thru 15JAN2020]**	1.362	1.141	1.626	0.0011
**[16JAN2021 thru 31JAN2021] vs [16JAN2020 thru 31JAN2020]**	1.282	1.069	1.536	0.0084
**[01FEB2021 thru 15FEB2021] vs [01FEB2020 thru 15FEB2020]**	1.225	1.011	1.485	0.0387
**[16FEB2021 thru 28FEB2021] vs [16FEB2020 thru 29FEB2020]**	1.215	1.003	1.473	0.0467
**[01MAR2020 thru 31MAR2021] vs [01FEB2019 thru 29FEB2020]**	1.481	1.375	1.596	< 0.0001

Indiana
Bimonthly Data

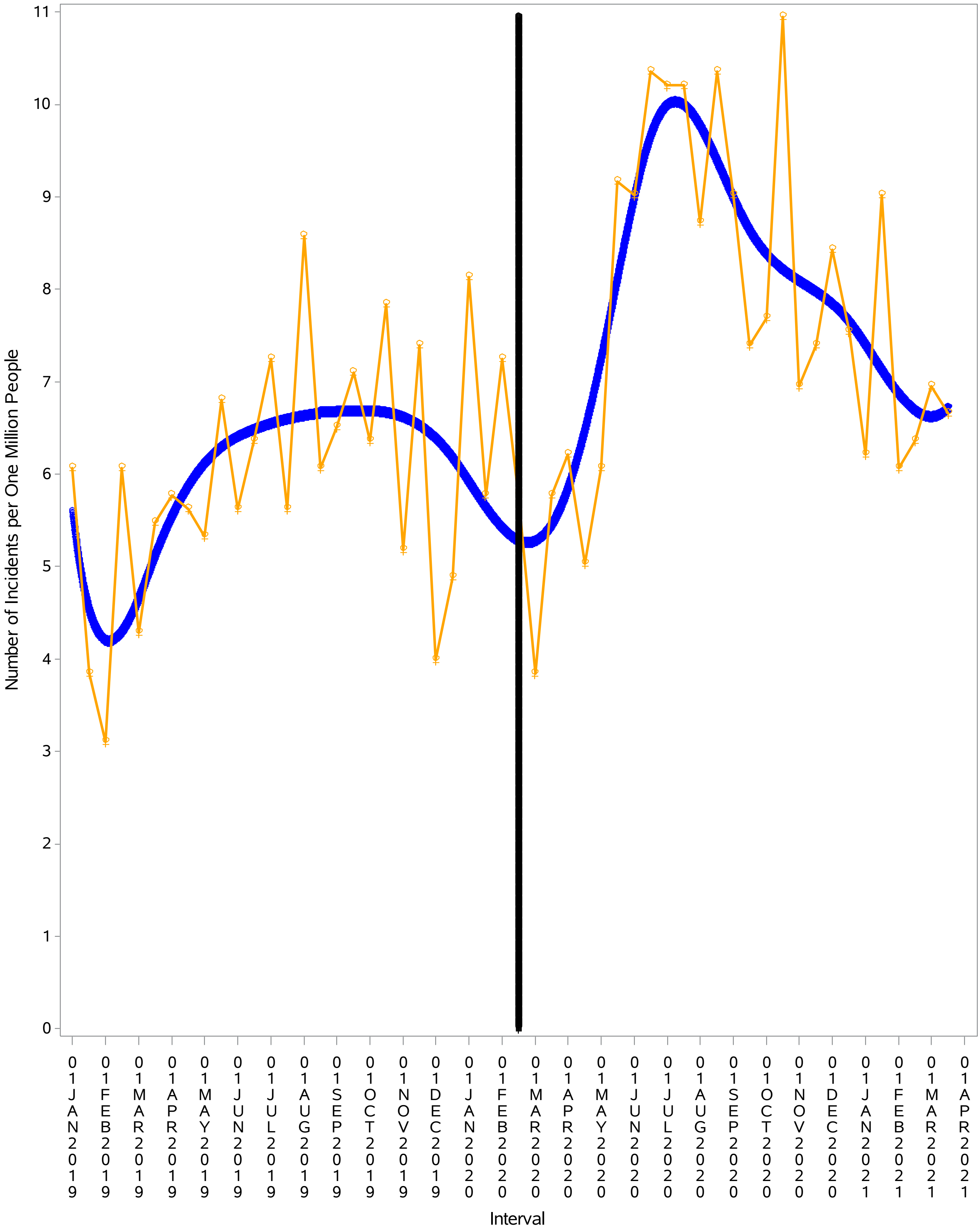

Indiana
Bimonthly Data

Comparison	IntensityRatio	IntensityRatio_LowerCL	IntensityRatio_UpperCL	P_Value
**[01MAR2020 thru 15MAR2020] vs [01MAR2019 thru 15MAR2019]**	1.133	0.875	1.468	0.3344
**[16MAR2020 thru 31MAR2020] vs [16MAR2019 thru 31MAR2019]**	1.064	0.824	1.375	0.6246
**[01APR2020 thru 15APR2020] vs [01APR2019 thru 15APR2019]**	1.057	0.819	1.364	0.6634
**[16APR2020 thru 30APR2020] vs [16APR2019 thru 30APR2019]**	1.102	0.870	1.397	0.411
**[01MAY2020 thru 15MAY2020] vs [01MAY2019 thru 15MAY2019]**	1.186	0.959	1.467	0.1122
**[16MAY2020 thru 31MAY2020] vs [16MAY2019 thru 31MAY2019]**	1.294	1.058	1.582	0.0135
**[01JUN2020 thru 15JUN2020] vs [01JUN2019 thru 15JUN2019]**	1.404	1.138	1.732	0.0022
**[16JUN2020 thru 30JUN2020] vs [16JUN2019 thru 30JUN2019]**	1.491	1.196	1.858	0.0007
**[01JUL2020 thru 15JUL2020] vs [01JUL2019 thru 15JUL2019]**	1.526	1.233	1.890	0.0003
**[16JUL2020 thru 31JUL2020] vs [16JUL2019 thru 31JUL2019]**	1.515	1.245	1.844	0.0001
**[01AUG2020 thru 15AUG2020] vs [01AUG2019 thru 15AUG2019]**	1.471	1.221	1.772	0.0001
**[16AUG2020 thru 31AUG2020] vs [16AUG2019 thru 31AUG2019]**	1.409	1.162	1.710	0.0009
**[01SEP2020 thru 15SEP2020] vs [01SEP2019 thru 15SEP2019]**	1.345	1.092	1.657	0.0065
**[16SEP2020 thru 30SEP2020] vs [16SEP2019 thru 30SEP2019]**	1.290	1.036	1.606	0.0237
**[01OCT2020 thru 15OCT2020] vs [01OCT2019 thru 15OCT2019]**	1.254	1.017	1.546	0.035
**[16OCT2020 thru 31OCT2020] vs [16OCT2019 thru 31OCT2019]**	1.231	1.012	1.498	0.0382
**[01NOV2020 thru 15NOV2020] vs [01NOV2019 thru 15NOV2019]**	1.222	1.005	1.485	0.0445
**[16NOV2020 thru 30NOV2020] vs [16NOV2019 thru 30NOV2019]**	1.222	0.990	1.508	0.0614
**[01DEC2020 thru 15DEC2020] vs [01DEC2019 thru 15DEC2019]**	1.228	0.979	1.542	0.0749
**[16DEC2020 thru 31DEC2020] vs [16DEC2019 thru 31DEC2019]**	1.239	0.984	1.560	0.0676
**[01JAN2021 thru 15JAN2021] vs [01JAN2020 thru 15JAN2020]**	1.251	1.002	1.562	0.0485
**[16JAN2021 thru 31JAN2021] vs [16JAN2020 thru 31JAN2020]**	1.261	1.005	1.583	0.0451
**[01FEB2021 thru 15FEB2021] vs [01FEB2020 thru 15FEB2020]**	1.268	0.994	1.616	0.0553
**[16FEB2021 thru 28FEB2021] vs [16FEB2020 thru 29FEB2020]**	1.267	0.989	1.621	0.0603
**[01MAR2020 thru 31MAR2021] vs [01FEB2019 thru 29FEB2020]**	1.298	1.176	1.433	< 0.0001

Iowa

Bimonthly Data

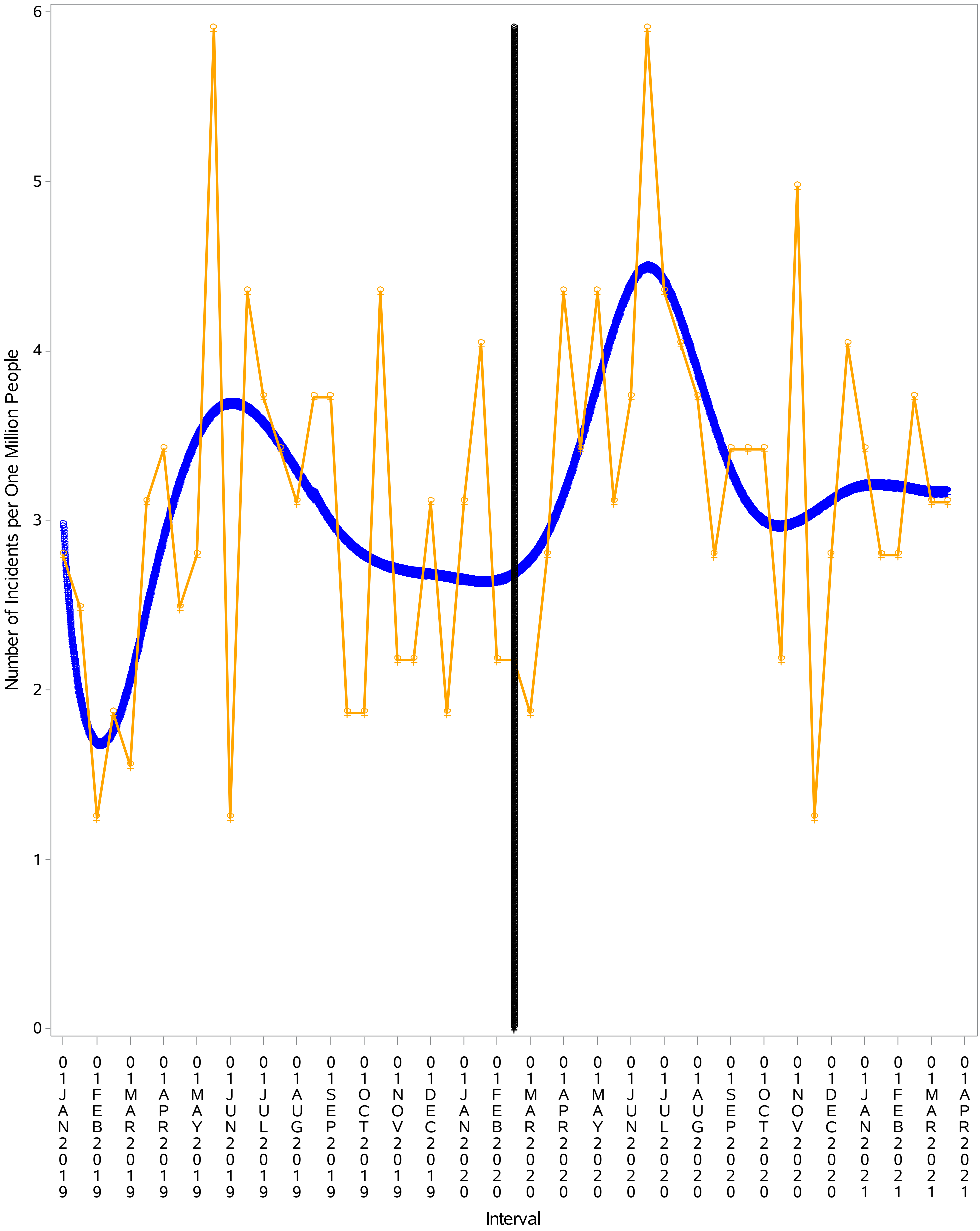

Iowa
Bimonthly Data

Comparison	IntensityRatio	IntensityRatio_LowerCL	IntensityRatio_UpperCL	P_Value
**[01MAR2020 thru 15MAR2020] vs [01MAR2019 thru 15MAR2019]**	1.347	0.868	2.091	0.1781
**[16MAR2020 thru 31MAR2020] vs [16MAR2019 thru 31MAR2019]**	1.179	0.775	1.793	0.4324
**[01APR2020 thru 15APR2020] vs [01APR2019 thru 15APR2019]**	1.092	0.723	1.650	0.6687
**[16APR2020 thru 30APR2020] vs [16APR2019 thru 30APR2019]**	1.070	0.731	1.567	0.7206
**[01MAY2020 thru 15MAY2020] vs [01MAY2019 thru 15MAY2019]**	1.091	0.775	1.535	0.6099
**[16MAY2020 thru 31MAY2020] vs [16MAY2019 thru 31MAY2019]**	1.136	0.820	1.574	0.4335
**[01JUN2020 thru 15JUN2020] vs [01JUN2019 thru 15JUN2019]**	1.189	0.844	1.674	0.3144
**[16JUN2020 thru 30JUN2020] vs [16JUN2019 thru 30JUN2019]**	1.227	0.855	1.761	0.2589
**[01JUL2020 thru 15JUL2020] vs [01JUL2019 thru 15JUL2019]**	1.235	0.868	1.756	0.2342
**[16JUL2020 thru 31JUL2020] vs [16JUL2019 thru 31JUL2019]**	1.215	0.874	1.688	0.24
**[01AUG2020 thru 15AUG2020] vs [01AUG2019 thru 15AUG2019]**	1.179	0.854	1.627	0.3094
**[16AUG2020 thru 31AUG2020] vs [16AUG2019 thru 31AUG2019]**	1.137	0.805	1.607	0.4569
**[01SEP2020 thru 15SEP2020] vs [01SEP2019 thru 15SEP2019]**	1.099	0.752	1.605	0.6178
**[16SEP2020 thru 30SEP2020] vs [16SEP2019 thru 30SEP2019]**	1.076	0.722	1.604	0.7121
**[01OCT2020 thru 15OCT2020] vs [01OCT2019 thru 15OCT2019]**	1.072	0.732	1.571	0.7153
**[16OCT2020 thru 31OCT2020] vs [16OCT2019 thru 31OCT2019]**	1.081	0.755	1.548	0.6632
**[01NOV2020 thru 15NOV2020] vs [01NOV2019 thru 15NOV2019]**	1.103	0.772	1.575	0.583
**[16NOV2020 thru 30NOV2020] vs [16NOV2019 thru 30NOV2019]**	1.131	0.772	1.658	0.5183
**[01DEC2020 thru 15DEC2020] vs [01DEC2019 thru 15DEC2019]**	1.162	0.771	1.751	0.464
**[16DEC2020 thru 31DEC2020] vs [16DEC2019 thru 31DEC2019]**	1.190	0.788	1.797	0.3999
**[01JAN2021 thru 15JAN2021] vs [01JAN2020 thru 15JAN2020]**	1.209	0.818	1.788	0.3329
**[16JAN2021 thru 31JAN2021] vs [16JAN2020 thru 31JAN2020]**	1.217	0.823	1.800	0.3175
**[01FEB2021 thru 15FEB2021] vs [01FEB2020 thru 15FEB2020]**	1.210	0.800	1.831	0.3582
**[16FEB2021 thru 28FEB2021] vs [16FEB2020 thru 29FEB2020]**	1.186	0.781	1.802	0.414
**[01MAR2020 thru 31MAR2021] vs [01FEB2019 thru 29FEB2020]**	1.207	1.017	1.432	0.0324

Kansas
Bimonthly Data

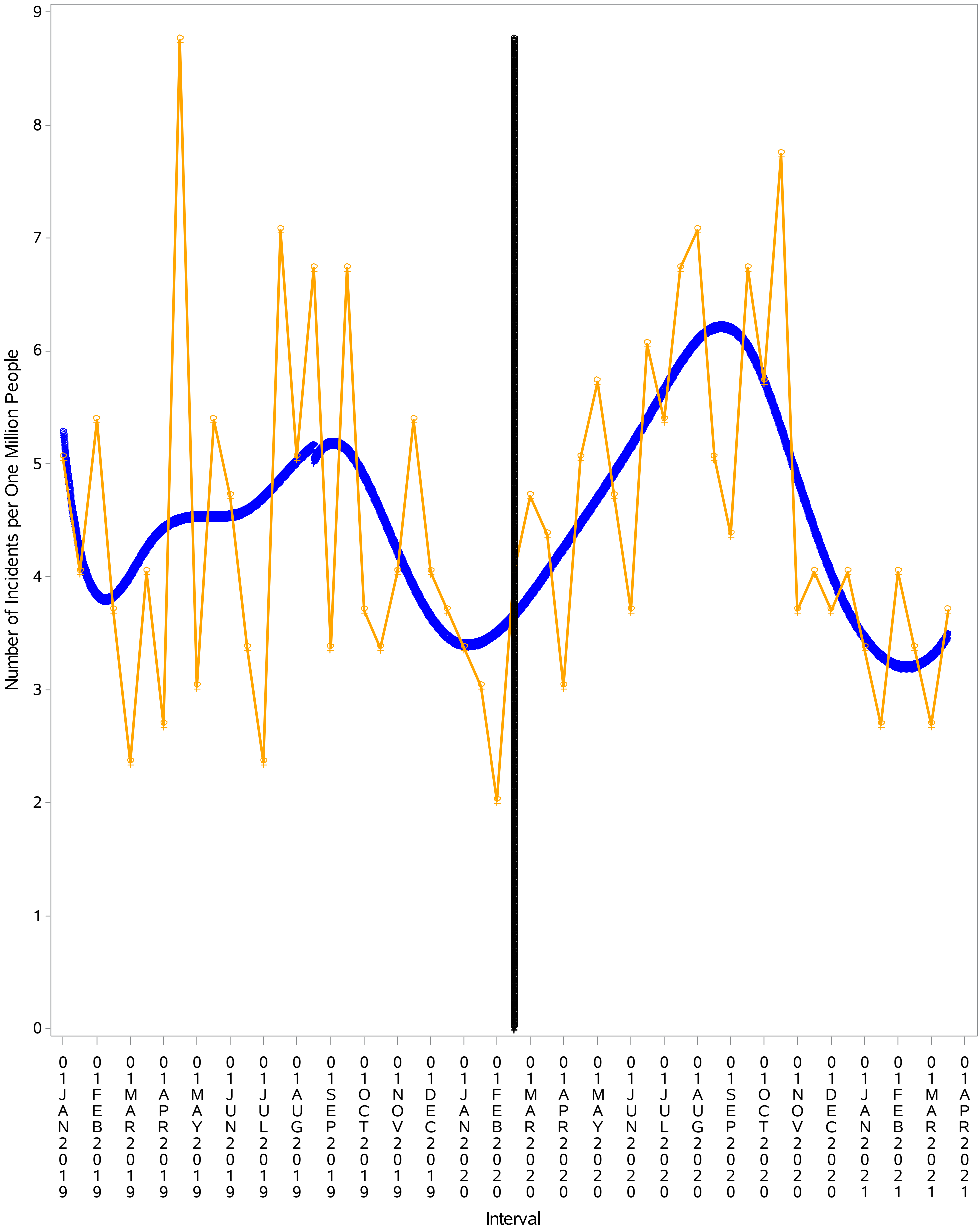

Kansas
Bimonthly Data

Comparison	IntensityRatio	IntensityRatio_LowerCL	IntensityRatio_UpperCL	P_Value
**[01MAR2020 thru 15MAR2020] vs [01MAR2019 thru 15MAR2019]**	0.957	0.642	1.427	0.8248
**[16MAR2020 thru 31MAR2020] vs [16MAR2019 thru 31MAR2019]**	0.951	0.635	1.422	0.8008
**[01APR2020 thru 15APR2020] vs [01APR2019 thru 15APR2019]**	0.962	0.643	1.440	0.8477
**[16APR2020 thru 30APR2020] vs [16APR2019 thru 30APR2019]**	0.992	0.681	1.445	0.9662
**[01MAY2020 thru 15MAY2020] vs [01MAY2019 thru 15MAY2019]**	1.035	0.735	1.458	0.8405
**[16MAY2020 thru 31MAY2020] vs [16MAY2019 thru 31MAY2019]**	1.085	0.777	1.515	0.6249
**[01JUN2020 thru 15JUN2020] vs [01JUN2019 thru 15JUN2019]**	1.135	0.797	1.615	0.4737
**[16JUN2020 thru 30JUN2020] vs [16JUN2019 thru 30JUN2019]**	1.176	0.812	1.703	0.3809
**[01JUL2020 thru 15JUL2020] vs [01JUL2019 thru 15JUL2019]**	1.202	0.841	1.718	0.304
**[16JUL2020 thru 31JUL2020] vs [16JUL2019 thru 31JUL2019]**	1.213	0.876	1.680	0.2388
**[01AUG2020 thru 15AUG2020] vs [01AUG2019 thru 15AUG2019]**	1.212	0.892	1.647	0.2125
**[16AUG2020 thru 31AUG2020] vs [16AUG2019 thru 31AUG2019]**	1.204	0.877	1.652	0.2447
**[01SEP2020 thru 15SEP2020] vs [01SEP2019 thru 15SEP2019]**	1.195	0.850	1.681	0.298
**[16SEP2020 thru 30SEP2020] vs [16SEP2019 thru 30SEP2019]**	1.179	0.825	1.686	0.3575
**[01OCT2020 thru 15OCT2020] vs [01OCT2019 thru 15OCT2019]**	1.168	0.829	1.647	0.3656
**[16OCT2020 thru 31OCT2020] vs [16OCT2019 thru 31OCT2019]**	1.161	0.835	1.614	0.3653
**[01NOV2020 thru 15NOV2020] vs [01NOV2019 thru 15NOV2019]**	1.149	0.817	1.617	0.4153
**[16NOV2020 thru 30NOV2020] vs [16NOV2019 thru 30NOV2019]**	1.132	0.775	1.652	0.5138
**[01DEC2020 thru 15DEC2020] vs [01DEC2019 thru 15DEC2019]**	1.105	0.731	1.670	0.629
**[16DEC2020 thru 31DEC2020] vs [16DEC2019 thru 31DEC2019]**	1.067	0.701	1.623	0.7584
**[01JAN2021 thru 15JAN2021] vs [01JAN2020 thru 15JAN2020]**	1.017	0.676	1.531	0.9337
**[16JAN2021 thru 31JAN2021] vs [16JAN2020 thru 31JAN2020]**	0.964	0.630	1.475	0.8631
**[01FEB2021 thru 15FEB2021] vs [01FEB2020 thru 15FEB2020]**	0.916	0.579	1.448	0.6999
**[16FEB2021 thru 28FEB2021] vs [16FEB2020 thru 29FEB2020]**	0.878	0.555	1.392	0.5729
**[01MAR2020 thru 31MAR2021] vs [01FEB2019 thru 29FEB2020]**	1.068	0.902	1.264	0.4364

Kentucky
Bimonthly Data

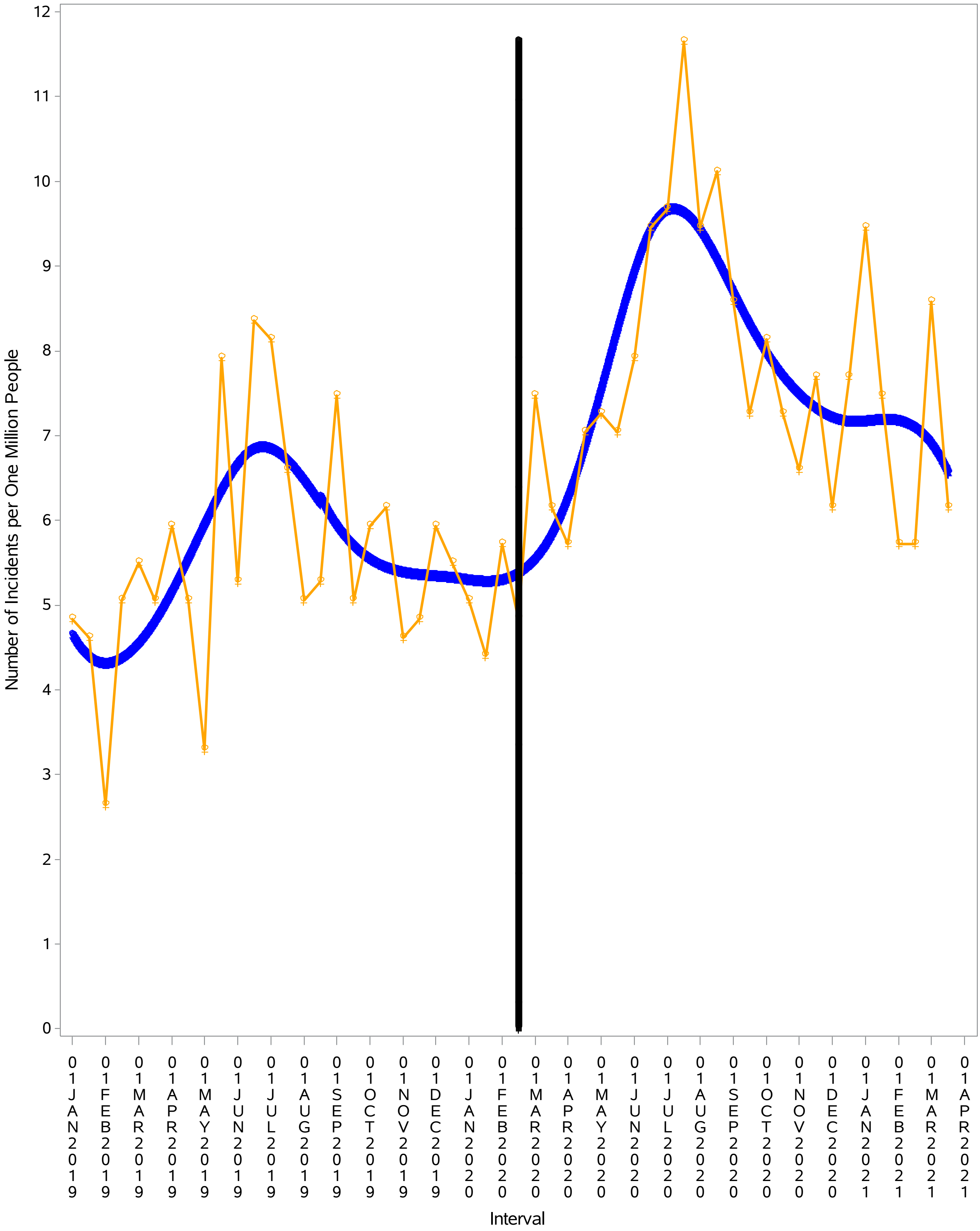

Kentucky
Bimonthly Data

Comparison	IntensityRatio	IntensityRatio_LowerCL	IntensityRatio_UpperCL	P_Value
**[01MAR2020 thru 15MAR2020] vs [01MAR2019 thru 15MAR2019]**	1.219	0.945	1.573	0.1242
**[16MAR2020 thru 31MAR2020] vs [16MAR2019 thru 31MAR2019]**	1.212	0.941	1.561	0.1329
**[01APR2020 thru 15APR2020] vs [01APR2019 thru 15APR2019]**	1.218	0.946	1.569	0.1235
**[16APR2020 thru 30APR2020] vs [16APR2019 thru 30APR2019]**	1.237	0.978	1.565	0.0746
**[01MAY2020 thru 15MAY2020] vs [01MAY2019 thru 15MAY2019]**	1.266	1.026	1.562	0.0285
**[16MAY2020 thru 31MAY2020] vs [16MAY2019 thru 31MAY2019]**	1.302	1.068	1.588	0.0104
**[01JUN2020 thru 15JUN2020] vs [01JUN2019 thru 15JUN2019]**	1.340	1.091	1.647	0.0065
**[16JUN2020 thru 30JUN2020] vs [16JUN2019 thru 30JUN2019]**	1.378	1.110	1.711	0.0047
**[01JUL2020 thru 15JUL2020] vs [01JUL2019 thru 15JUL2019]**	1.410	1.143	1.740	0.002
**[16JUL2020 thru 31JUL2020] vs [16JUL2019 thru 31JUL2019]**	1.437	1.183	1.745	0.0005
**[01AUG2020 thru 15AUG2020] vs [01AUG2019 thru 15AUG2019]**	1.455	1.208	1.753	0.0002
**[16AUG2020 thru 31AUG2020] vs [16AUG2019 thru 31AUG2019]**	1.465	1.204	1.784	0.0003
**[01SEP2020 thru 15SEP2020] vs [01SEP2019 thru 15SEP2019]**	1.464	1.182	1.814	0.0009
**[16SEP2020 thru 30SEP2020] vs [16SEP2019 thru 30SEP2019]**	1.457	1.162	1.827	0.0017
**[01OCT2020 thru 15OCT2020] vs [01OCT2019 thru 15OCT2019]**	1.438	1.157	1.787	0.0016
**[16OCT2020 thru 31OCT2020] vs [16OCT2019 thru 31OCT2019]**	1.414	1.153	1.734	0.0014
**[01NOV2020 thru 15NOV2020] vs [01NOV2019 thru 15NOV2019]**	1.388	1.133	1.701	0.0022
**[16NOV2020 thru 30NOV2020] vs [16NOV2019 thru 30NOV2019]**	1.366	1.099	1.699	0.0061
**[01DEC2020 thru 15DEC2020] vs [01DEC2019 thru 15DEC2019]**	1.351	1.068	1.708	0.0133
**[16DEC2020 thru 31DEC2020] vs [16DEC2019 thru 31DEC2019]**	1.346	1.062	1.707	0.0154
**[01JAN2021 thru 15JAN2021] vs [01JAN2020 thru 15JAN2020]**	1.354	1.079	1.699	0.0101
**[16JAN2021 thru 31JAN2021] vs [16JAN2020 thru 31JAN2020]**	1.362	1.085	1.709	0.0089
**[01FEB2021 thru 15FEB2021] vs [01FEB2020 thru 15FEB2020]**	1.355	1.068	1.720	0.0138
**[16FEB2021 thru 28FEB2021] vs [16FEB2020 thru 29FEB2020]**	1.320	1.038	1.678	0.0245
**[01MAR2020 thru 31MAR2021] vs [01FEB2019 thru 29FEB2020]**	1.368	1.238	1.511	< 0.0001

Louisana

Bimonthly Data

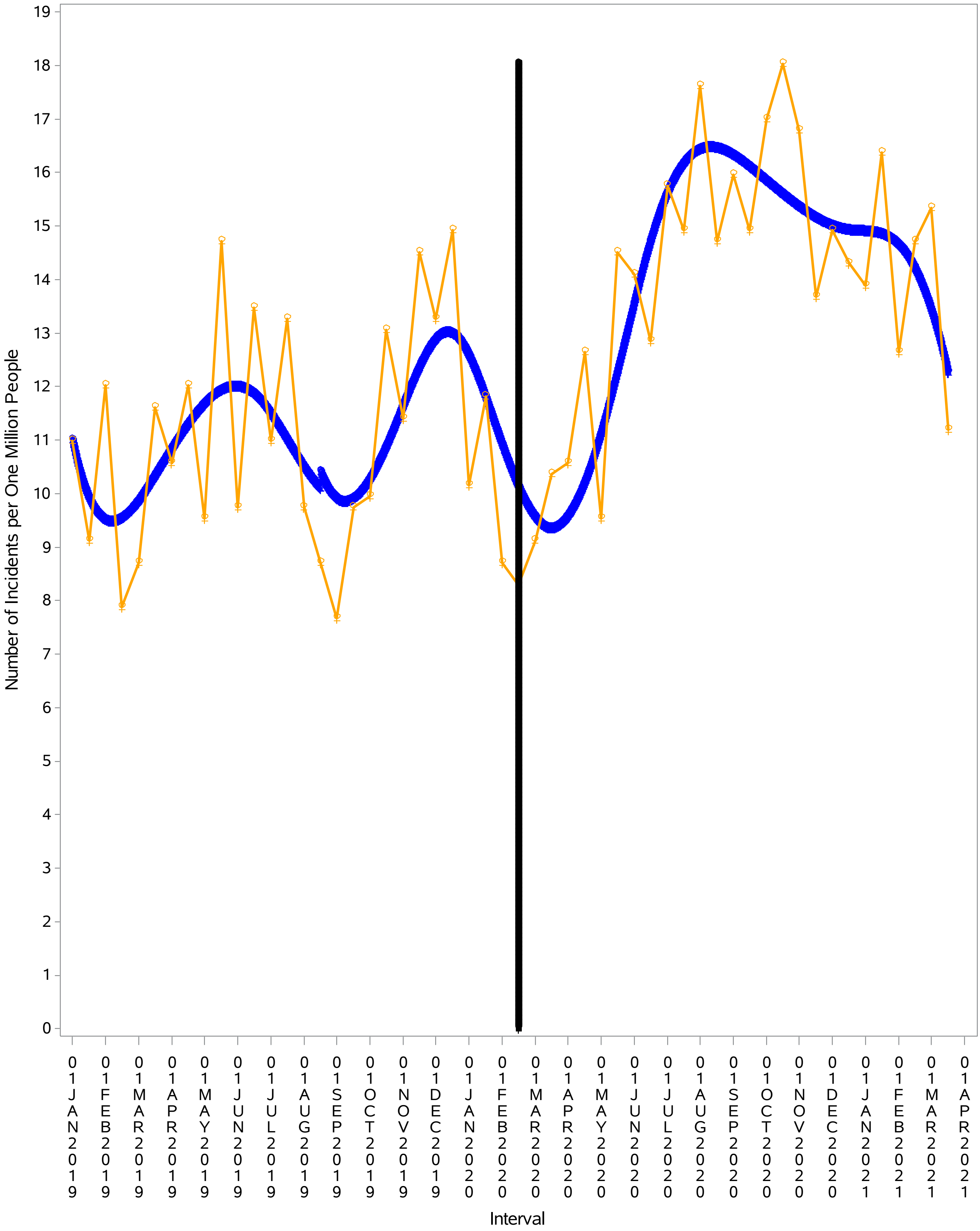

Louisana
Bimonthly Data

Comparison	IntensityRatio	IntensityRatio_LowerCL	IntensityRatio_UpperCL	P_Value
**[01MAR2020 thru 15MAR2020] vs [01MAR2019 thru 15MAR2019]**	0.969	0.796	1.181	0.753
**[16MAR2020 thru 31MAR2020] vs [16MAR2019 thru 31MAR2019]**	0.905	0.741	1.105	0.3188
**[01APR2020 thru 15APR2020] vs [01APR2019 thru 15APR2019]**	0.885	0.724	1.081	0.2235
**[16APR2020 thru 30APR2020] vs [16APR2019 thru 30APR2019]**	0.903	0.749	1.088	0.2752
**[01MAY2020 thru 15MAY2020] vs [01MAY2019 thru 15MAY2019]**	0.953	0.805	1.128	0.5673
**[16MAY2020 thru 31MAY2020] vs [16MAY2019 thru 31MAY2019]**	1.030	0.875	1.212	0.7197
**[01JUN2020 thru 15JUN2020] vs [01JUN2019 thru 15JUN2019]**	1.128	0.950	1.340	0.1644
**[16JUN2020 thru 30JUN2020] vs [16JUN2019 thru 30JUN2019]**	1.241	1.036	1.487	0.0204
**[01JUL2020 thru 15JUL2020] vs [01JUL2019 thru 15JUL2019]**	1.358	1.140	1.618	0.001
**[16JUL2020 thru 31JUL2020] vs [16JUL2019 thru 31JUL2019]**	1.470	1.250	1.728	< 0.0001
**[01AUG2020 thru 15AUG2020] vs [01AUG2019 thru 15AUG2019]**	1.565	1.342	1.825	< 0.0001
**[16AUG2020 thru 31AUG2020] vs [16AUG2019 thru 31AUG2019]**	1.631	1.390	1.913	< 0.0001
**[01SEP2020 thru 15SEP2020] vs [01SEP2019 thru 15SEP2019]**	1.648	1.388	1.958	< 0.0001
**[16SEP2020 thru 30SEP2020] vs [16SEP2019 thru 30SEP2019]**	1.626	1.357	1.947	< 0.0001
**[01OCT2020 thru 15OCT2020] vs [01OCT2019 thru 15OCT2019]**	1.543	1.300	1.833	< 0.0001
**[16OCT2020 thru 31OCT2020] vs [16OCT2019 thru 31OCT2019]**	1.433	1.223	1.680	< 0.0001
**[01NOV2020 thru 15NOV2020] vs [01NOV2019 thru 15NOV2019]**	1.321	1.133	1.541	0.0007
**[16NOV2020 thru 30NOV2020] vs [16NOV2019 thru 30NOV2019]**	1.227	1.043	1.443	0.0148
**[01DEC2020 thru 15DEC2020] vs [01DEC2019 thru 15DEC2019]**	1.165	0.979	1.386	0.0832
**[16DEC2020 thru 31DEC2020] vs [16DEC2019 thru 31DEC2019]**	1.149	0.964	1.369	0.1181
**[01JAN2021 thru 15JAN2021] vs [01JAN2020 thru 15JAN2020]**	1.186	1.003	1.402	0.0468
**[16JAN2021 thru 31JAN2021] vs [16JAN2020 thru 31JAN2020]**	1.259	1.062	1.492	0.0091
**[01FEB2021 thru 15FEB2021] vs [01FEB2020 thru 15FEB2020]**	1.342	1.119	1.611	0.0022
**[16FEB2021 thru 28FEB2021] vs [16FEB2020 thru 29FEB2020]**	1.403	1.163	1.692	0.0007
**[01MAR2020 thru 31MAR2021] vs [01FEB2019 thru 29FEB2020]**	1.248	1.155	1.349	< 0.0001

Maine
Bimonthly Data

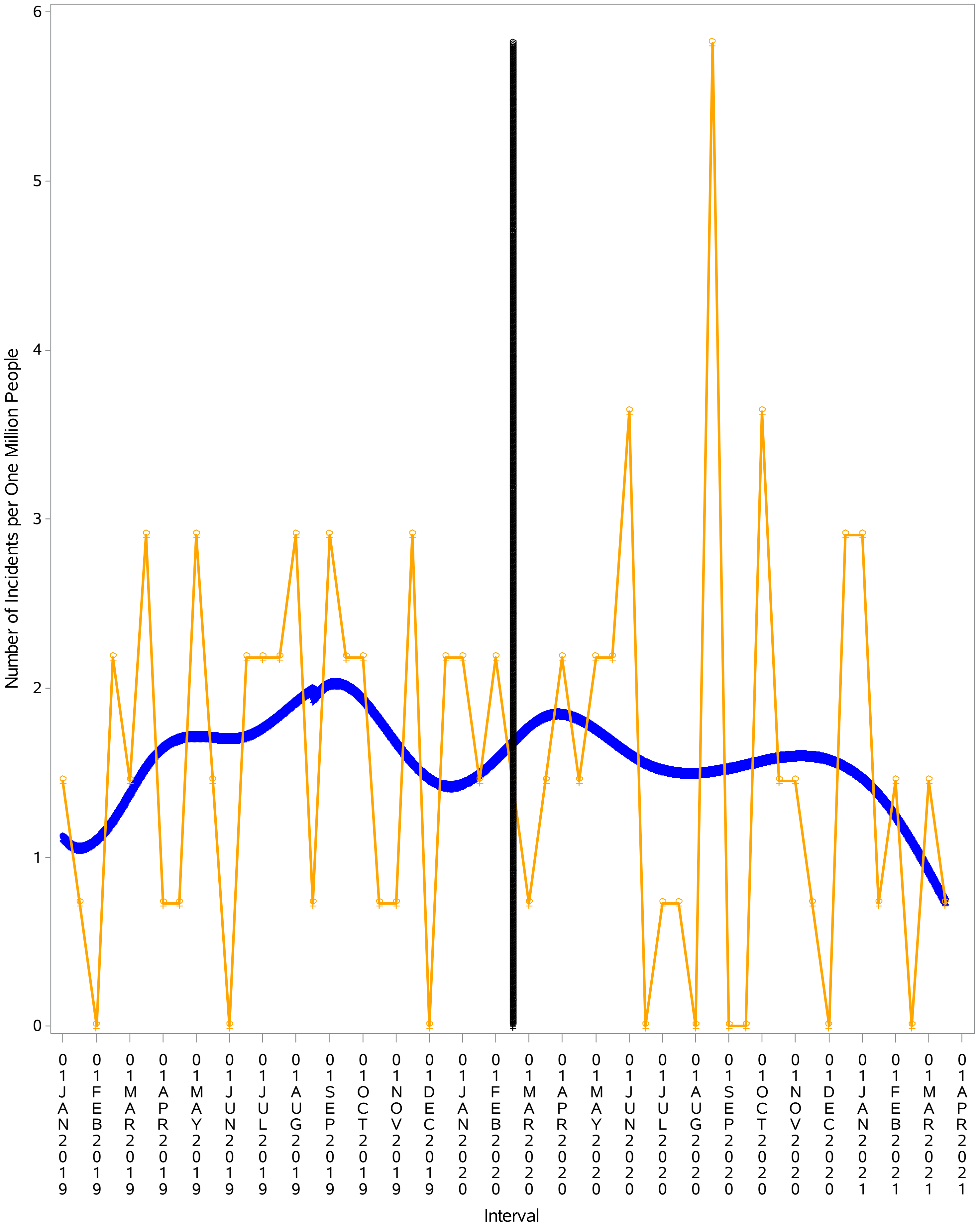

Maine
Bimonthly Data

Comparison	IntensityRatio	IntensityRatio_LowerCL	IntensityRatio_UpperCL	P_Value
**[01MAR2020 thru 15MAR2020] vs [01MAR2019 thru 15MAR2019]**	1.289	0.437	3.804	0.6383
**[16MAR2020 thru 31MAR2020] vs [16MAR2019 thru 31MAR2019]**	1.196	0.417	3.426	0.7338
**[01APR2020 thru 15APR2020] vs [01APR2019 thru 15APR2019]**	1.122	0.391	3.221	0.8261
**[16APR2020 thru 30APR2020] vs [16APR2019 thru 30APR2019]**	1.067	0.395	2.885	0.8959
**[01MAY2020 thru 15MAY2020] vs [01MAY2019 thru 15MAY2019]**	1.023	0.405	2.580	0.9611
**[16MAY2020 thru 31MAY2020] vs [16MAY2019 thru 31MAY2019]**	0.984	0.392	2.473	0.9722
**[01JUN2020 thru 15JUN2020] vs [01JUN2019 thru 15JUN2019]**	0.947	0.353	2.538	0.911
**[16JUN2020 thru 30JUN2020] vs [16JUN2019 thru 30JUN2019]**	0.906	0.321	2.560	0.8488
**[01JUL2020 thru 15JUL2020] vs [01JUL2019 thru 15JUL2019]**	0.861	0.314	2.357	0.7659
**[16JUL2020 thru 31JUL2020] vs [16JUL2019 thru 31JUL2019]**	0.817	0.323	2.070	0.6637
**[01AUG2020 thru 15AUG2020] vs [01AUG2019 thru 15AUG2019]**	0.781	0.322	1.895	0.5768
**[16AUG2020 thru 31AUG2020] vs [16AUG2019 thru 31AUG2019]**	0.758	0.301	1.904	0.5465
**[01SEP2020 thru 15SEP2020] vs [01SEP2019 thru 15SEP2019]**	0.754	0.280	2.032	0.569
**[16SEP2020 thru 30SEP2020] vs [16SEP2019 thru 30SEP2019]**	0.768	0.274	2.152	0.6083
**[01OCT2020 thru 15OCT2020] vs [01OCT2019 thru 15OCT2019]**	0.814	0.306	2.161	0.6721
**[16OCT2020 thru 31OCT2020] vs [16OCT2019 thru 31OCT2019]**	0.880	0.351	2.203	0.7794
**[01NOV2020 thru 15NOV2020] vs [01NOV2019 thru 15NOV2019]**	0.957	0.379	2.418	0.9238
**[16NOV2020 thru 30NOV2020] vs [16NOV2019 thru 30NOV2019]**	1.029	0.375	2.825	0.9541
**[01DEC2020 thru 15DEC2020] vs [01DEC2019 thru 15DEC2019]**	1.078	0.363	3.202	0.8904
**[16DEC2020 thru 31DEC2020] vs [16DEC2019 thru 31DEC2019]**	1.080	0.361	3.228	0.888
**[01JAN2021 thru 15JAN2021] vs [01JAN2020 thru 15JAN2020]**	1.023	0.354	2.952	0.966
**[16JAN2021 thru 31JAN2021] vs [16JAN2020 thru 31JAN2020]**	0.918	0.301	2.800	0.8779
**[01FEB2021 thru 15FEB2021] vs [01FEB2020 thru 15FEB2020]**	0.786	0.233	2.656	0.6924
**[16FEB2021 thru 28FEB2021] vs [16FEB2020 thru 29FEB2020]**	0.647	0.187	2.236	0.4825
**[01MAR2020 thru 31MAR2021] vs [01FEB2019 thru 29FEB2020]**	0.909	0.570	1.452	0.6838

Maryland
Bimonthly Data

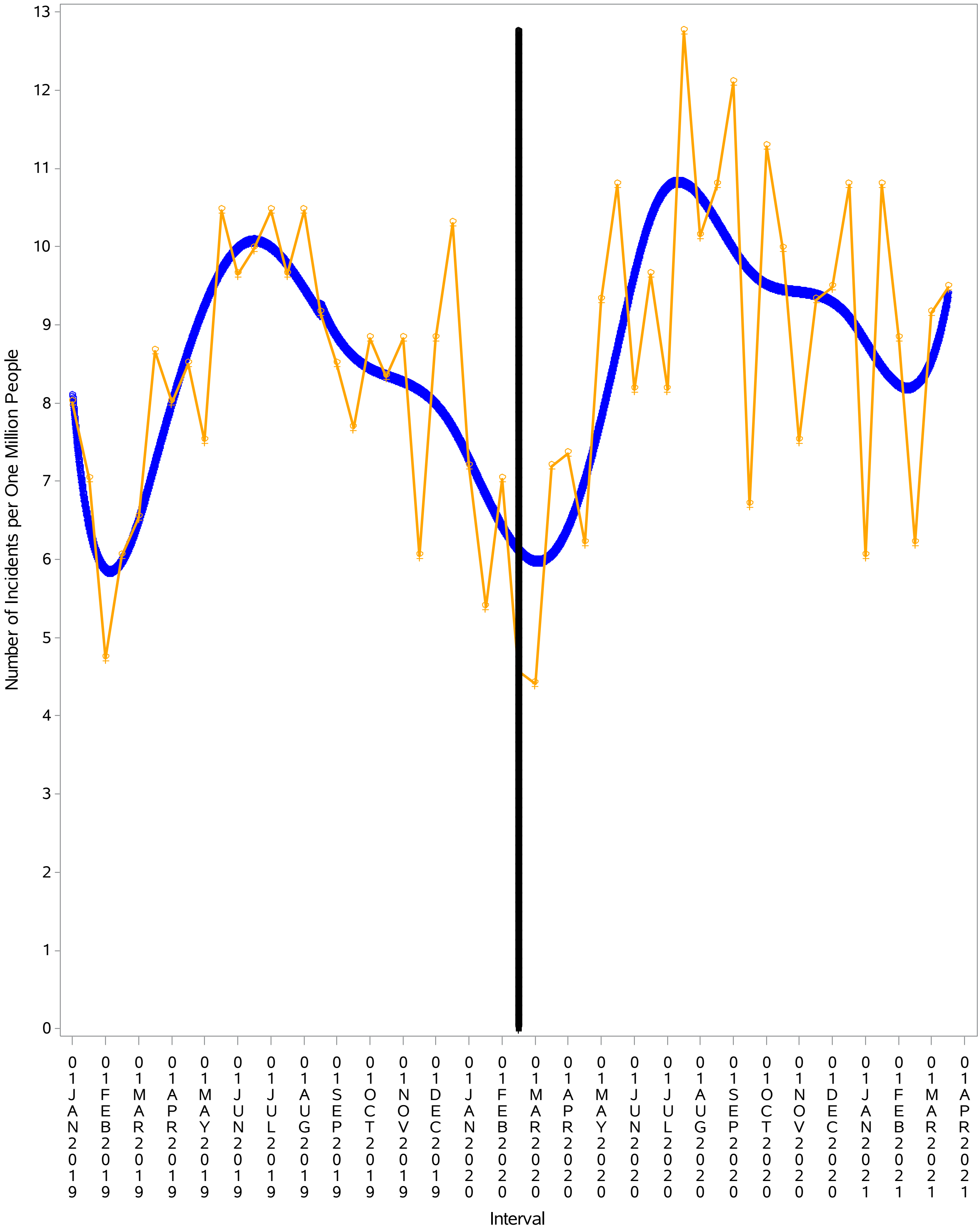

Maryland
Bimonthly Data

Comparison	IntensityRatio	IntensityRatio_LowerCL	IntensityRatio_UpperCL	P_Value
**[01MAR2020 thru 15MAR2020] vs [01MAR2019 thru 15MAR2019]**	0.920	0.717	1.181	0.5031
**[16MAR2020 thru 31MAR2020] vs [16MAR2019 thru 31MAR2019]**	0.837	0.654	1.071	0.1524
**[01APR2020 thru 15APR2020] vs [01APR2019 thru 15APR2019]**	0.803	0.629	1.024	0.076
**[16APR2020 thru 30APR2020] vs [16APR2019 thru 30APR2019]**	0.809	0.646	1.013	0.0642
**[01MAY2020 thru 15MAY2020] vs [01MAY2019 thru 15MAY2019]**	0.844	0.689	1.034	0.0986
**[16MAY2020 thru 31MAY2020] vs [16MAY2019 thru 31MAY2019]**	0.900	0.742	1.092	0.2767
**[01JUN2020 thru 15JUN2020] vs [01JUN2019 thru 15JUN2019]**	0.966	0.790	1.182	0.7311
**[16JUN2020 thru 30JUN2020] vs [16JUN2019 thru 30JUN2019]**	1.030	0.834	1.271	0.782
**[01JUL2020 thru 15JUL2020] vs [01JUL2019 thru 15JUL2019]**	1.077	0.878	1.321	0.4665
**[16JUL2020 thru 31JUL2020] vs [16JUL2019 thru 31JUL2019]**	1.107	0.918	1.337	0.28
**[01AUG2020 thru 15AUG2020] vs [01AUG2019 thru 15AUG2019]**	1.123	0.938	1.345	0.1999
**[16AUG2020 thru 31AUG2020] vs [16AUG2019 thru 31AUG2019]**	1.129	0.934	1.365	0.2042
**[01SEP2020 thru 15SEP2020] vs [01SEP2019 thru 15SEP2019]**	1.127	0.917	1.385	0.2498
**[16SEP2020 thru 30SEP2020] vs [16SEP2019 thru 30SEP2019]**	1.127	0.907	1.400	0.2719
**[01OCT2020 thru 15OCT2020] vs [01OCT2019 thru 15OCT2019]**	1.129	0.918	1.388	0.2442
**[16OCT2020 thru 31OCT2020] vs [16OCT2019 thru 31OCT2019]**	1.132	0.932	1.373	0.2047
**[01NOV2020 thru 15NOV2020] vs [01NOV2019 thru 15NOV2019]**	1.139	0.939	1.382	0.1815
**[16NOV2020 thru 30NOV2020] vs [16NOV2019 thru 30NOV2019]**	1.150	0.933	1.417	0.1845
**[01DEC2020 thru 15DEC2020] vs [01DEC2019 thru 15DEC2019]**	1.164	0.929	1.460	0.1809
**[16DEC2020 thru 31DEC2020] vs [16DEC2019 thru 31DEC2019]**	1.183	0.941	1.487	0.1453
**[01JAN2021 thru 15JAN2021] vs [01JAN2020 thru 15JAN2020]**	1.207	0.969	1.504	0.0918
**[16JAN2021 thru 31JAN2021] vs [16JAN2020 thru 31JAN2020]**	1.239	0.990	1.550	0.0608
**[01FEB2021 thru 15FEB2021] vs [01FEB2020 thru 15FEB2020]**	1.283	1.008	1.633	0.0432
**[16FEB2021 thru 28FEB2021] vs [16FEB2020 thru 29FEB2020]**	1.345	1.051	1.721	0.0199
**[01MAR2020 thru 31MAR2021] vs [01FEB2019 thru 29FEB2020]**	1.095	0.995	1.204	0.0636

Massachusetts
Bimonthly Data

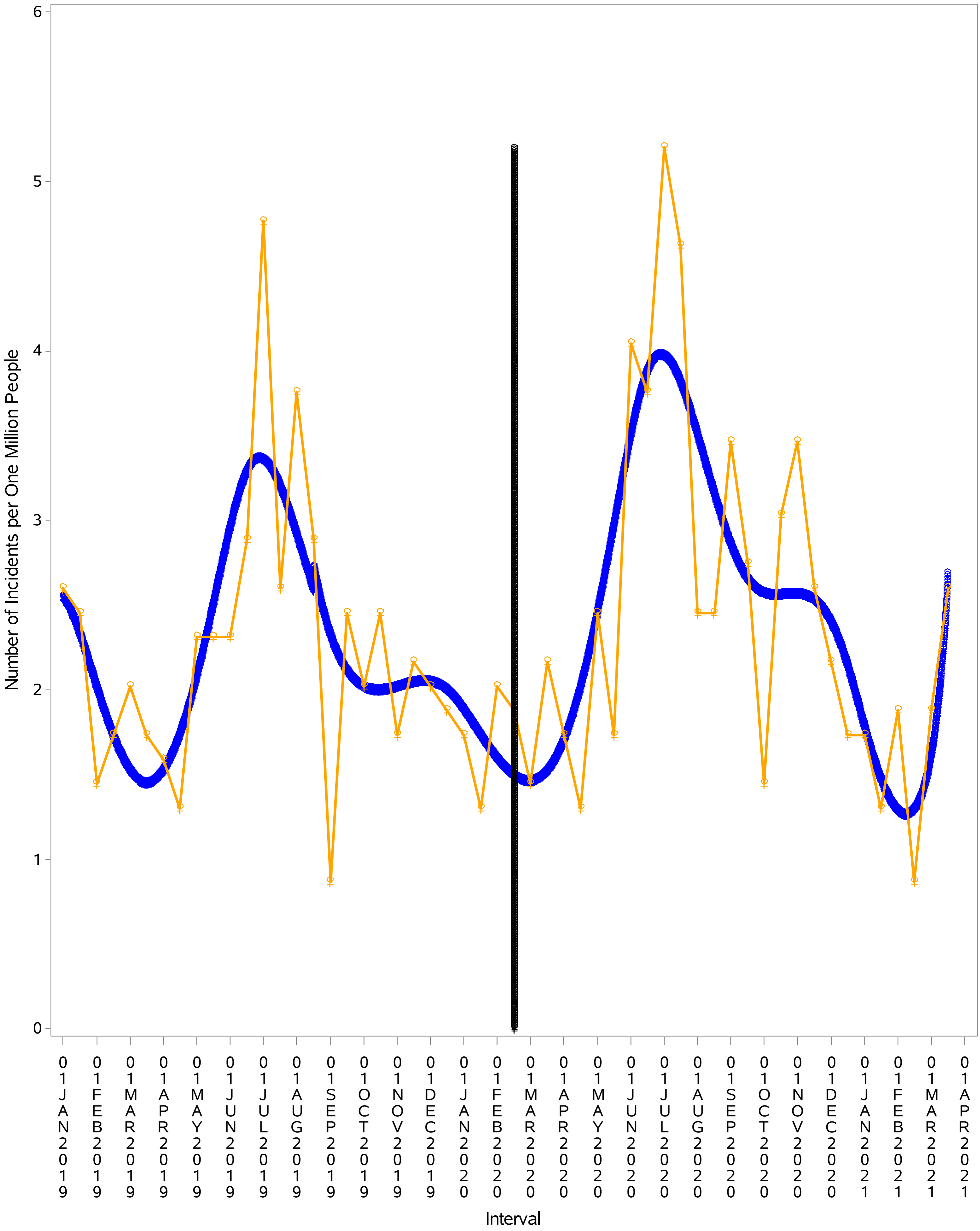

Massachusetts
Bimonthly Data

Comparison	IntensityRatio	IntensityRatio_LowerCL	IntensityRatio_UpperCL	P_Value
**[01MAR2020 thru 15MAR2020] vs [01MAR2019 thru 15MAR2019]**	0.960	0.651	1.415	0.8316
**[16MAR2020 thru 31MAR2020] vs [16MAR2019 thru 31MAR2019]**	1.051	0.705	1.566	0.8044
**[01APR2020 thru 15APR2020] vs [01APR2019 thru 15APR2019]**	1.119	0.751	1.666	0.5729
**[16APR2020 thru 30APR2020] vs [16APR2019 thru 30APR2019]**	1.161	0.806	1.671	0.4137
**[01MAY2020 thru 15MAY2020] vs [01MAY2019 thru 15MAY2019]**	1.181	0.860	1.622	0.2947
**[16MAY2020 thru 31MAY2020] vs [16MAY2019 thru 31MAY2019]**	1.187	0.892	1.579	0.2339
**[01JUN2020 thru 15JUN2020] vs [01JUN2019 thru 15JUN2019]**	1.184	0.890	1.575	0.2397
**[16JUN2020 thru 30JUN2020] vs [16JUN2019 thru 30JUN2019]**	1.180	0.879	1.584	0.2618
**[01JUL2020 thru 15JUL2020] vs [01JUL2019 thru 15JUL2019]**	1.183	0.890	1.572	0.2406
**[16JUL2020 thru 31JUL2020] vs [16JUL2019 thru 31JUL2019]**	1.191	0.914	1.552	0.1908
**[01AUG2020 thru 15AUG2020] vs [01AUG2019 thru 15AUG2019]**	1.203	0.927	1.562	0.1599
**[16AUG2020 thru 31AUG2020] vs [16AUG2019 thru 31AUG2019]**	1.219	0.918	1.619	0.1661
**[01SEP2020 thru 15SEP2020] vs [01SEP2019 thru 15SEP2019]**	1.229	0.898	1.682	0.1917
**[16SEP2020 thru 30SEP2020] vs [16SEP2019 thru 30SEP2019]**	1.256	0.901	1.751	0.1729
**[01OCT2020 thru 15OCT2020] vs [01OCT2019 thru 15OCT2019]**	1.279	0.929	1.759	0.1275
**[16OCT2020 thru 31OCT2020] vs [16OCT2019 thru 31OCT2019]**	1.282	0.948	1.733	0.1037
**[01NOV2020 thru 15NOV2020] vs [01NOV2019 thru 15NOV2019]**	1.272	0.939	1.724	0.1173
**[16NOV2020 thru 30NOV2020] vs [16NOV2019 thru 30NOV2019]**	1.236	0.889	1.719	0.2008
**[01DEC2020 thru 15DEC2020] vs [01DEC2019 thru 15DEC2019]**	1.169	0.818	1.669	0.3821
**[16DEC2020 thru 31DEC2020] vs [16DEC2019 thru 31DEC2019]**	1.067	0.742	1.536	0.7191
**[01JAN2021 thru 15JAN2021] vs [01JAN2020 thru 15JAN2020]**	0.945	0.657	1.358	0.754
**[16JAN2021 thru 31JAN2021] vs [16JAN2020 thru 31JAN2020]**	0.846	0.572	1.251	0.3937
**[01FEB2021 thru 15FEB2021] vs [01FEB2020 thru 15FEB2020]**	0.808	0.524	1.247	0.3272
**[16FEB2021 thru 28FEB2021] vs [16FEB2020 thru 29FEB2020]**	0.868	0.560	1.348	0.5211
**[01MAR2020 thru 31MAR2021] vs [01FEB2019 thru 29FEB2020]**	1.112	0.958	1.292	0.1579

Michigan
Bimonthly Data

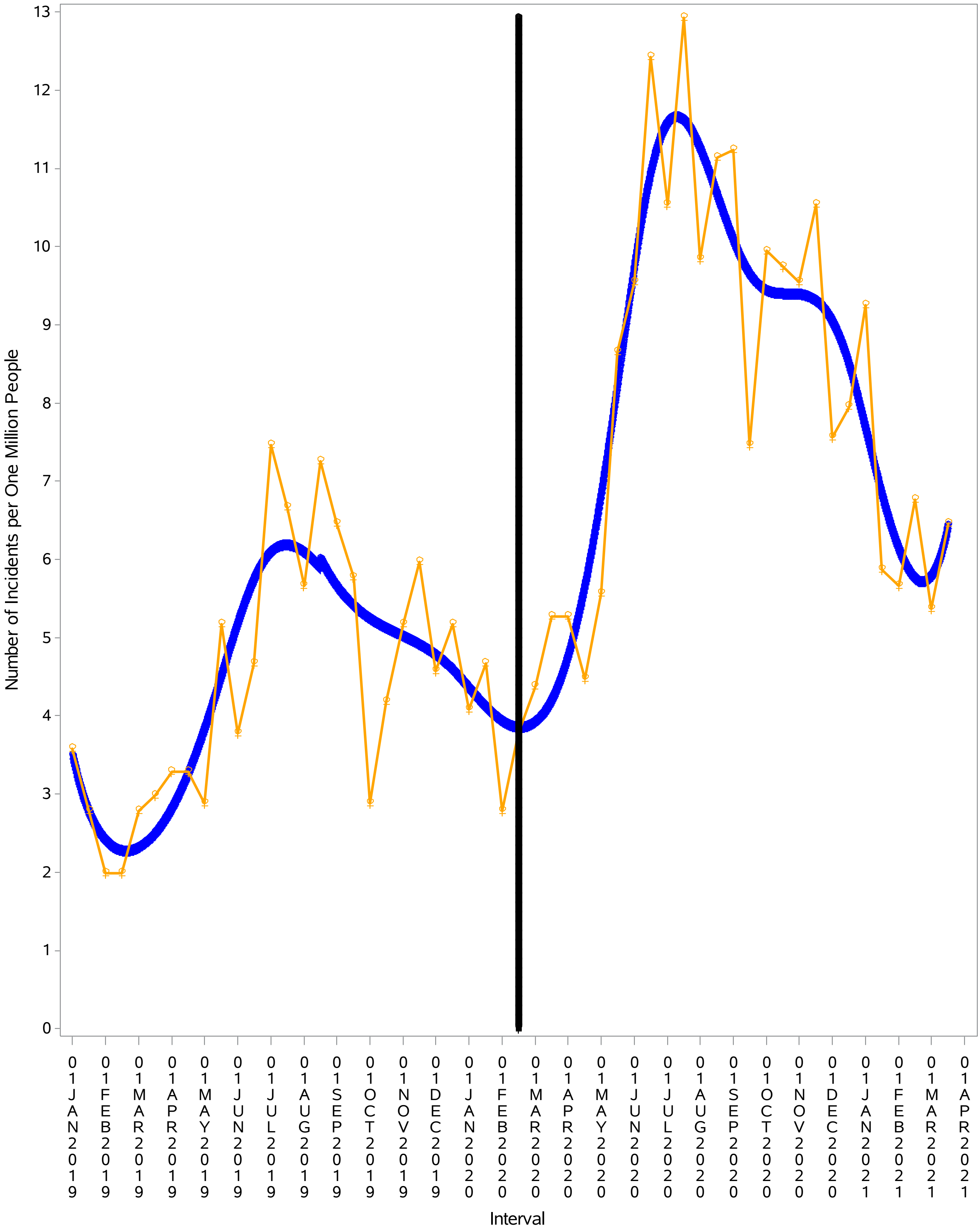

Michigan
Bimonthly Data

Comparison	IntensityRatio	IntensityRatio_LowerCL	IntensityRatio_UpperCL	P_Value
**[01MAR2020 thru 15MAR2020] vs [01MAR2019 thru 15MAR2019]**	1.693	1.262	2.273	0.0008
**[16MAR2020 thru 31MAR2020] vs [16MAR2019 thru 31MAR2019]**	1.683	1.264	2.241	0.0007
**[01APR2020 thru 15APR2020] vs [01APR2019 thru 15APR2019]**	1.695	1.274	2.257	0.0006
**[16APR2020 thru 30APR2020] vs [16APR2019 thru 30APR2019]**	1.731	1.329	2.253	0.0001
**[01MAY2020 thru 15MAY2020] vs [01MAY2019 thru 15MAY2019]**	1.779	1.415	2.236	< 0.0001
**[16MAY2020 thru 31MAY2020] vs [16MAY2019 thru 31MAY2019]**	1.831	1.496	2.242	< 0.0001
**[01JUN2020 thru 15JUN2020] vs [01JUN2019 thru 15JUN2019]**	1.876	1.539	2.288	< 0.0001
**[16JUN2020 thru 30JUN2020] vs [16JUN2019 thru 30JUN2019]**	1.902	1.550	2.335	< 0.0001
**[01JUL2020 thru 15JUL2020] vs [01JUL2019 thru 15JUL2019]**	1.901	1.558	2.318	< 0.0001
**[16JUL2020 thru 31JUL2020] vs [16JUL2019 thru 31JUL2019]**	1.878	1.565	2.253	< 0.0001
**[01AUG2020 thru 15AUG2020] vs [01AUG2019 thru 15AUG2019]**	1.845	1.554	2.190	< 0.0001
**[16AUG2020 thru 31AUG2020] vs [16AUG2019 thru 31AUG2019]**	1.812	1.516	2.165	< 0.0001
**[01SEP2020 thru 15SEP2020] vs [01SEP2019 thru 15SEP2019]**	1.783	1.469	2.164	< 0.0001
**[16SEP2020 thru 30SEP2020] vs [16SEP2019 thru 30SEP2019]**	1.782	1.451	2.189	< 0.0001
**[01OCT2020 thru 15OCT2020] vs [01OCT2019 thru 15OCT2019]**	1.805	1.481	2.199	< 0.0001
**[16OCT2020 thru 31OCT2020] vs [16OCT2019 thru 31OCT2019]**	1.835	1.525	2.208	< 0.0001
**[01NOV2020 thru 15NOV2020] vs [01NOV2019 thru 15NOV2019]**	1.871	1.557	2.247	< 0.0001
**[16NOV2020 thru 30NOV2020] vs [16NOV2019 thru 30NOV2019]**	1.894	1.555	2.308	< 0.0001
**[01DEC2020 thru 15DEC2020] vs [01DEC2019 thru 15DEC2019]**	1.892	1.527	2.344	< 0.0001
**[16DEC2020 thru 31DEC2020] vs [16DEC2019 thru 31DEC2019]**	1.849	1.483	2.304	< 0.0001
**[01JAN2021 thru 15JAN2021] vs [01JAN2020 thru 15JAN2020]**	1.762	1.419	2.187	< 0.0001
**[16JAN2021 thru 31JAN2021] vs [16JAN2020 thru 31JAN2020]**	1.657	1.327	2.068	< 0.0001
**[01FEB2021 thru 15FEB2021] vs [01FEB2020 thru 15FEB2020]**	1.560	1.233	1.974	0.0004
**[16FEB2021 thru 28FEB2021] vs [16FEB2020 thru 29FEB2020]**	1.494	1.177	1.896	0.0015
**[01MAR2020 thru 31MAR2021] vs [01FEB2019 thru 29FEB2020]**	1.833	1.660	2.024	< 0.0001

Minnesota
Bimonthly Data

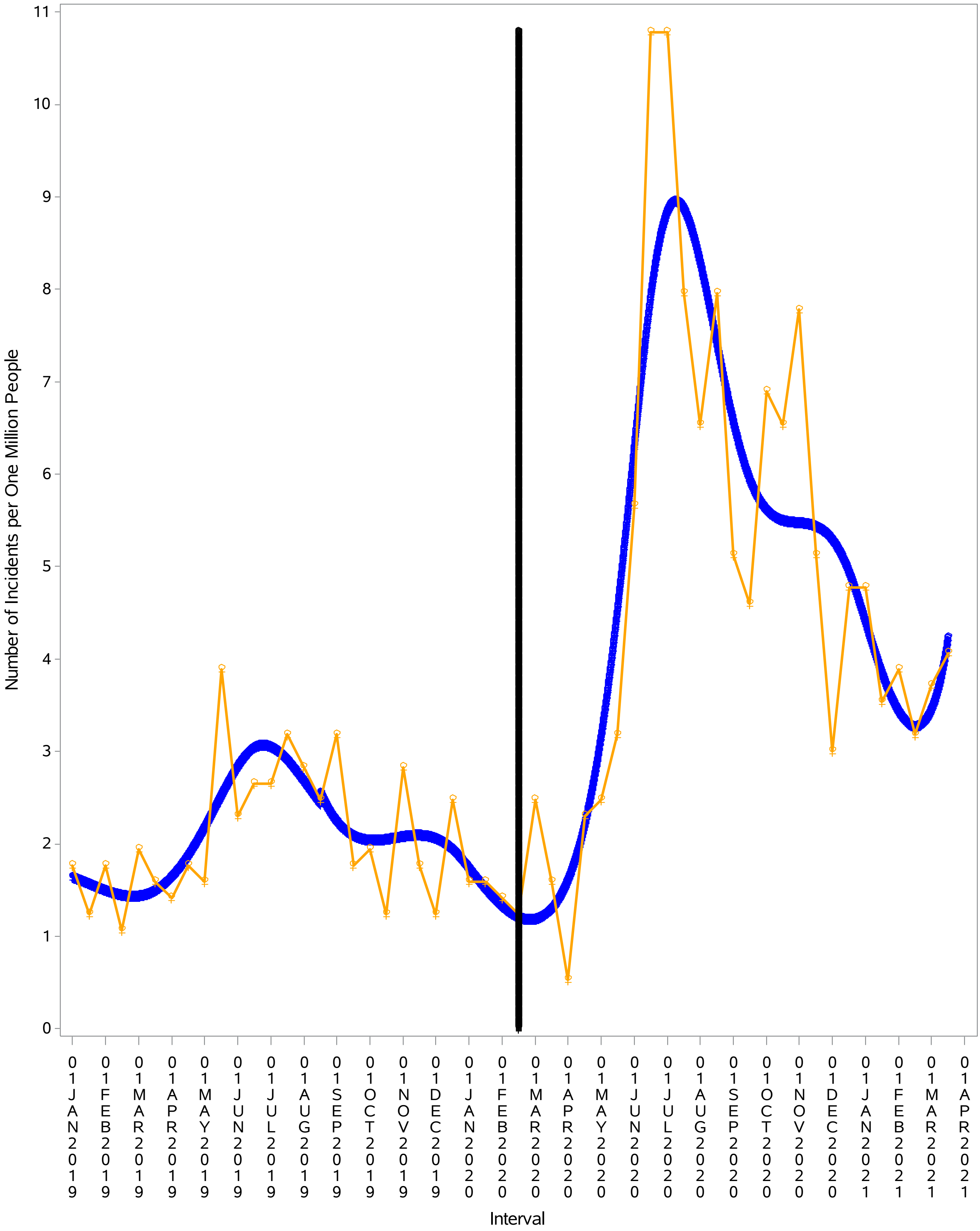

Minnesota
Bimonthly Data

Comparison	IntensityRatio	IntensityRatio_LowerCL	IntensityRatio_UpperCL	P_Value
**[01MAR2020 thru 15MAR2020] vs [01MAR2019 thru 15MAR2019]**	0.826	0.495	1.377	0.4549
**[16MAR2020 thru 31MAR2020] vs [16MAR2019 thru 31MAR2019]**	0.869	0.524	1.444	0.5807
**[01APR2020 thru 15APR2020] vs [01APR2019 thru 15APR2019]**	0.984	0.596	1.626	0.9495
**[16APR2020 thru 30APR2020] vs [16APR2019 thru 30APR2019]**	1.178	0.746	1.860	0.4734
**[01MAY2020 thru 15MAY2020] vs [01MAY2019 thru 15MAY2019]**	1.455	0.986	2.148	0.0585
**[16MAY2020 thru 31MAY2020] vs [16MAY2019 thru 31MAY2019]**	1.812	1.292	2.540	0.001
**[01JUN2020 thru 15JUN2020] vs [01JUN2019 thru 15JUN2019]**	2.219	1.598	3.083	< 0.0001
**[16JUN2020 thru 30JUN2020] vs [16JUN2019 thru 30JUN2019]**	2.611	1.860	3.667	< 0.0001
**[01JUL2020 thru 15JUL2020] vs [01JUL2019 thru 15JUL2019]**	2.899	2.084	4.032	< 0.0001
**[16JUL2020 thru 31JUL2020] vs [16JUL2019 thru 31JUL2019]**	3.055	2.252	4.144	< 0.0001
**[01AUG2020 thru 15AUG2020] vs [01AUG2019 thru 15AUG2019]**	3.092	2.305	4.147	< 0.0001
**[16AUG2020 thru 31AUG2020] vs [16AUG2019 thru 31AUG2019]**	3.042	2.223	4.163	< 0.0001
**[01SEP2020 thru 15SEP2020] vs [01SEP2019 thru 15SEP2019]**	2.927	2.071	4.136	< 0.0001
**[16SEP2020 thru 30SEP2020] vs [16SEP2019 thru 30SEP2019]**	2.839	1.961	4.110	< 0.0001
**[01OCT2020 thru 15OCT2020] vs [01OCT2019 thru 15OCT2019]**	2.760	1.931	3.944	< 0.0001
**[16OCT2020 thru 31OCT2020] vs [16OCT2019 thru 31OCT2019]**	2.684	1.923	3.747	< 0.0001
**[01NOV2020 thru 15NOV2020] vs [01NOV2019 thru 15NOV2019]**	2.633	1.896	3.657	< 0.0001
**[16NOV2020 thru 30NOV2020] vs [16NOV2019 thru 30NOV2019]**	2.596	1.826	3.690	< 0.0001
**[01DEC2020 thru 15DEC2020] vs [01DEC2019 thru 15DEC2019]**	2.568	1.749	3.771	< 0.0001
**[16DEC2020 thru 31DEC2020] vs [16DEC2019 thru 31DEC2019]**	2.548	1.709	3.798	< 0.0001
**[01JAN2021 thru 15JAN2021] vs [01JAN2020 thru 15JAN2020]**	2.535	1.708	3.763	< 0.0001
**[16JAN2021 thru 31JAN2021] vs [16JAN2020 thru 31JAN2020]**	2.545	1.704	3.799	< 0.0001
**[01FEB2021 thru 15FEB2021] vs [01FEB2020 thru 15FEB2020]**	2.596	1.707	3.947	< 0.0001
**[16FEB2021 thru 28FEB2021] vs [16FEB2020 thru 29FEB2020]**	2.710	1.772	4.145	< 0.0001
**[01MAR2020 thru 31MAR2021] vs [01FEB2019 thru 29FEB2020]**	2.202	1.858	2.610	< 0.0001

Mississippi
Bimonthly Data

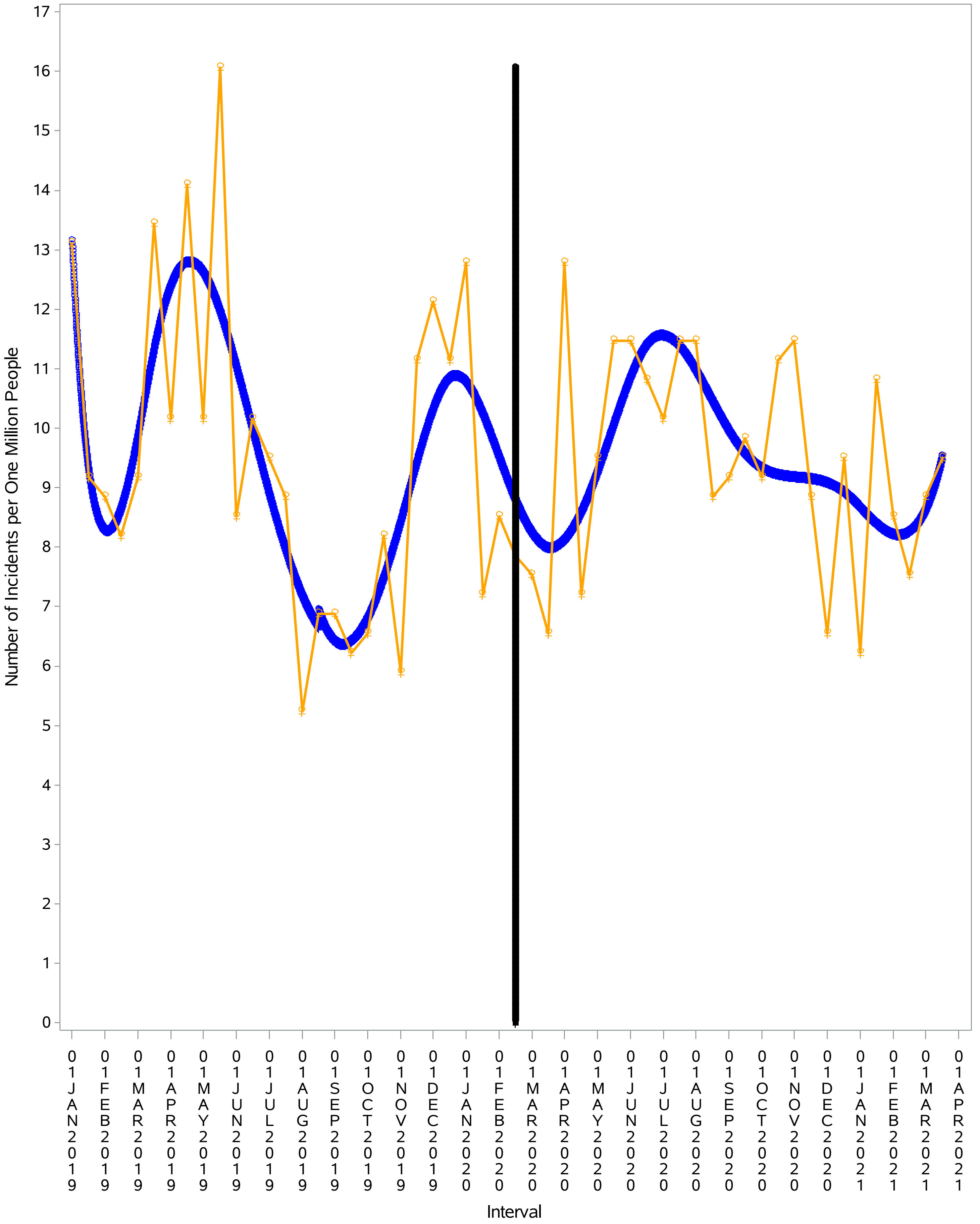

Mississippi
Bimonthly Data

Comparison	IntensityRatio	IntensityRatio_LowerCL	IntensityRatio_UpperCL	P_Value
**[01MAR2020 thru 15MAR2020] vs [01MAR2019 thru 15MAR2019]**	0.835	0.656	1.063	0.1392
**[16MAR2020 thru 31MAR2020] vs [16MAR2019 thru 31MAR2019]**	0.705	0.554	0.897	0.0055
**[01APR2020 thru 15APR2020] vs [01APR2019 thru 15APR2019]**	0.656	0.517	0.833	0.0009
**[16APR2020 thru 30APR2020] vs [16APR2019 thru 30APR2019]**	0.671	0.537	0.838	0.0008
**[01MAY2020 thru 15MAY2020] vs [01MAY2019 thru 15MAY2019]**	0.735	0.599	0.902	0.0041
**[16MAY2020 thru 31MAY2020] vs [16MAY2019 thru 31MAY2019]**	0.842	0.686	1.034	0.0985
**[01JUN2020 thru 15JUN2020] vs [01JUN2019 thru 15JUN2019]**	0.984	0.788	1.230	0.8879
**[16JUN2020 thru 30JUN2020] vs [16JUN2019 thru 30JUN2019]**	1.146	0.905	1.451	0.251
**[01JUL2020 thru 15JUL2020] vs [01JUL2019 thru 15JUL2019]**	1.301	1.033	1.638	0.0266
**[16JUL2020 thru 31JUL2020] vs [16JUL2019 thru 31JUL2019]**	1.432	1.153	1.778	0.0018
**[01AUG2020 thru 15AUG2020] vs [01AUG2019 thru 15AUG2019]**	1.526	1.232	1.888	0.0003
**[16AUG2020 thru 31AUG2020] vs [16AUG2019 thru 31AUG2019]**	1.570	1.250	1.973	0.0003
**[01SEP2020 thru 15SEP2020] vs [01SEP2019 thru 15SEP2019]**	1.551	1.210	1.987	0.0009
**[16SEP2020 thru 30SEP2020] vs [16SEP2019 thru 30SEP2019]**	1.489	1.149	1.931	0.0035
**[01OCT2020 thru 15OCT2020] vs [01OCT2019 thru 15OCT2019]**	1.372	1.072	1.755	0.0132
**[16OCT2020 thru 31OCT2020] vs [16OCT2019 thru 31OCT2019]**	1.229	0.979	1.543	0.0738
**[01NOV2020 thru 15NOV2020] vs [01NOV2019 thru 15NOV2019]**	1.091	0.875	1.359	0.4307
**[16NOV2020 thru 30NOV2020] vs [16NOV2019 thru 30NOV2019]**	0.971	0.771	1.222	0.7959
**[01DEC2020 thru 15DEC2020] vs [01DEC2019 thru 15DEC2019]**	0.879	0.689	1.123	0.2955
**[16DEC2020 thru 31DEC2020] vs [16DEC2019 thru 31DEC2019]**	0.823	0.644	1.051	0.1158
**[01JAN2021 thru 15JAN2021] vs [01JAN2020 thru 15JAN2020]**	0.804	0.637	1.016	0.0673
**[16JAN2021 thru 31JAN2021] vs [16JAN2020 thru 31JAN2020]**	0.819	0.644	1.042	0.1023
**[01FEB2021 thru 15FEB2021] vs [01FEB2020 thru 15FEB2020]**	0.865	0.665	1.124	0.2698
**[16FEB2021 thru 28FEB2021] vs [16FEB2020 thru 29FEB2020]**	0.941	0.719	1.230	0.6474
**[01MAR2020 thru 31MAR2021] vs [01FEB2019 thru 29FEB2020]**	1.019	0.918	1.132	0.7138

Missouri
Bimonthly Data

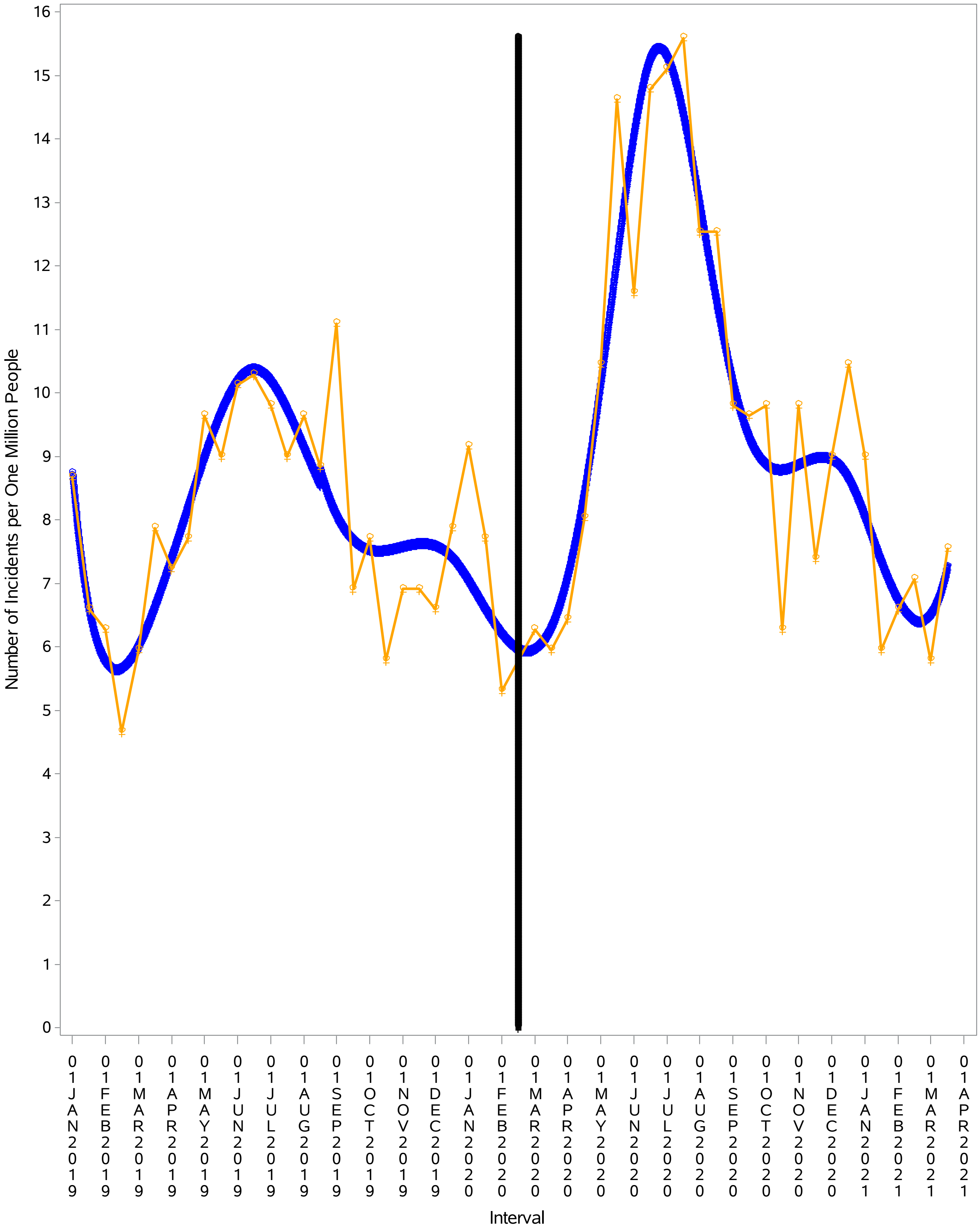

Missouri
Bimonthly Data

Comparison	IntensityRatio	IntensityRatio_LowerCL	IntensityRatio_UpperCL	P_Value
**[01MAR2020 thru 15MAR2020] vs [01MAR2019 thru 15MAR2019]**	0.992	0.813	1.211	0.9375
**[16MAR2020 thru 31MAR2020] vs [16MAR2019 thru 31MAR2019]**	0.950	0.780	1.156	0.5991
**[01APR2020 thru 15APR2020] vs [01APR2019 thru 15APR2019]**	0.963	0.793	1.170	0.6979
**[16APR2020 thru 30APR2020] vs [16APR2019 thru 30APR2019]**	1.027	0.859	1.227	0.765
**[01MAY2020 thru 15MAY2020] vs [01MAY2019 thru 15MAY2019]**	1.129	0.965	1.321	0.127
**[16MAY2020 thru 31MAY2020] vs [16MAY2019 thru 31MAY2019]**	1.253	1.083	1.450	0.0032
**[01JUN2020 thru 15JUN2020] vs [01JUN2019 thru 15JUN2019]**	1.378	1.186	1.601	0.0001
**[16JUN2020 thru 30JUN2020] vs [16JUN2019 thru 30JUN2019]**	1.471	1.257	1.721	< 0.0001
**[01JUL2020 thru 15JUL2020] vs [01JUL2019 thru 15JUL2019]**	1.502	1.289	1.750	< 0.0001
**[16JUL2020 thru 31JUL2020] vs [16JUL2019 thru 31JUL2019]**	1.476	1.280	1.703	< 0.0001
**[01AUG2020 thru 15AUG2020] vs [01AUG2019 thru 15AUG2019]**	1.415	1.230	1.628	< 0.0001
**[16AUG2020 thru 31AUG2020] vs [16AUG2019 thru 31AUG2019]**	1.338	1.150	1.558	0.0004
**[01SEP2020 thru 15SEP2020] vs [01SEP2019 thru 15SEP2019]**	1.260	1.067	1.490	0.0078
**[16SEP2020 thru 30SEP2020] vs [16SEP2019 thru 30SEP2019]**	1.209	1.013	1.442	0.0358
**[01OCT2020 thru 15OCT2020] vs [01OCT2019 thru 15OCT2019]**	1.182	0.998	1.400	0.0527
**[16OCT2020 thru 31OCT2020] vs [16OCT2019 thru 31OCT2019]**	1.169	0.997	1.371	0.0547
**[01NOV2020 thru 15NOV2020] vs [01NOV2019 thru 15NOV2019]**	1.171	0.998	1.373	0.0523
**[16NOV2020 thru 30NOV2020] vs [16NOV2019 thru 30NOV2019]**	1.177	0.991	1.397	0.0622
**[01DEC2020 thru 15DEC2020] vs [01DEC2019 thru 15DEC2019]**	1.179	0.980	1.418	0.0791
**[16DEC2020 thru 31DEC2020] vs [16DEC2019 thru 31DEC2019]**	1.169	0.970	1.409	0.0987
**[01JAN2021 thru 15JAN2021] vs [01JAN2020 thru 15JAN2020]**	1.144	0.953	1.372	0.144
**[16JAN2021 thru 31JAN2021] vs [16JAN2020 thru 31JAN2020]**	1.112	0.920	1.344	0.2664
**[01FEB2021 thru 15FEB2021] vs [01FEB2020 thru 15FEB2020]**	1.085	0.883	1.333	0.4267
**[16FEB2021 thru 28FEB2021] vs [16FEB2020 thru 29FEB2020]**	1.075	0.872	1.325	0.4873
**[01MAR2020 thru 31MAR2021] vs [01FEB2019 thru 29FEB2020]**	1.194	1.106	1.290	< 0.0001

Montana
Bimonthly Data

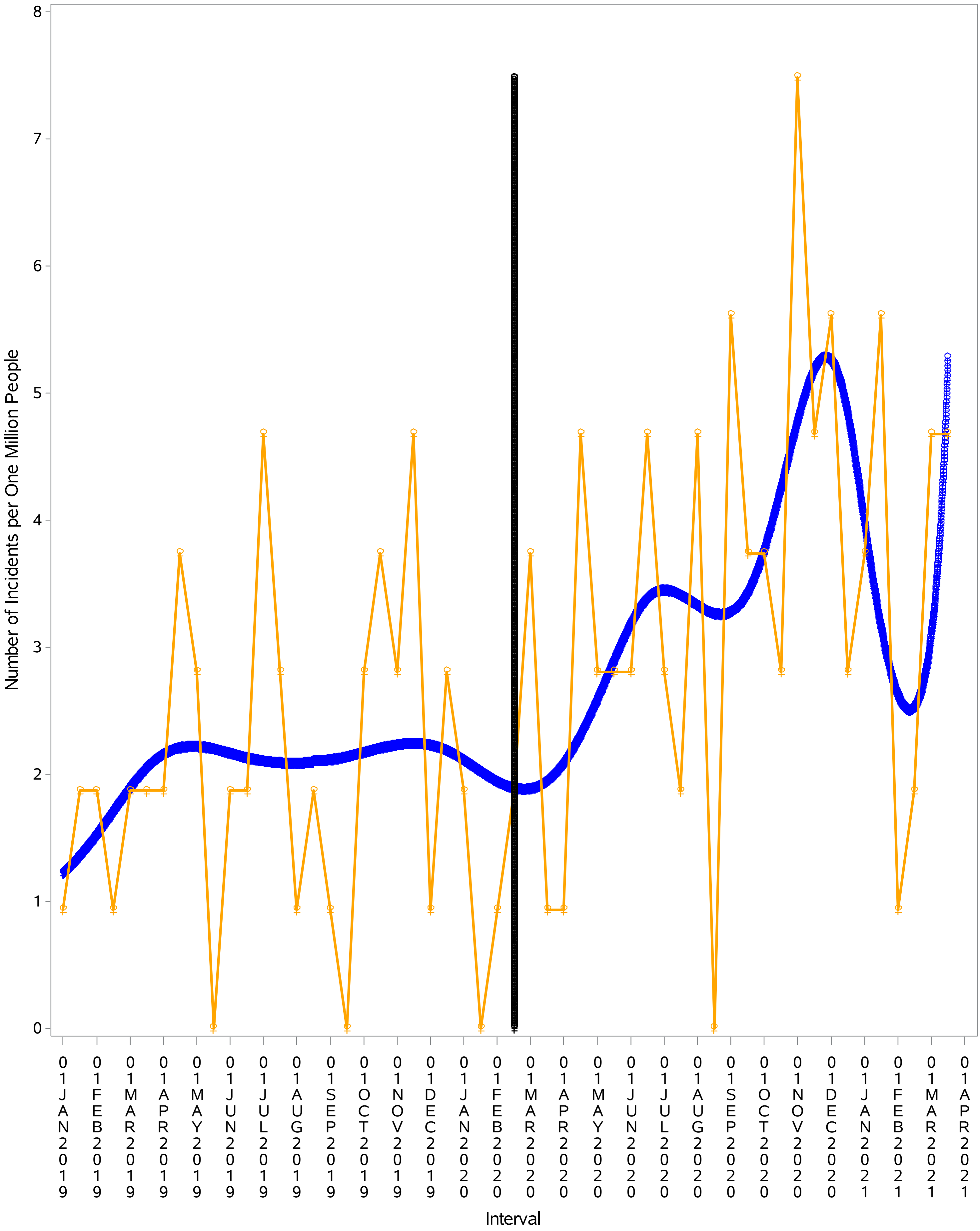

Montana
Bimonthly Data

Comparison	IntensityRatio	IntensityRatio_LowerCL	IntensityRatio_UpperCL	P_Value
**[01MAR2020 thru 15MAR2020] vs [01MAR2019 thru 15MAR2019]**	0.998	0.420	2.368	0.9956
**[16MAR2020 thru 31MAR2020] vs [16MAR2019 thru 31MAR2019]**	0.950	0.405	2.225	0.9032
**[01APR2020 thru 15APR2020] vs [01APR2019 thru 15APR2019]**	0.970	0.413	2.274	0.9419
**[16APR2020 thru 30APR2020] vs [16APR2019 thru 30APR2019]**	1.046	0.473	2.315	0.9099
**[01MAY2020 thru 15MAY2020] vs [01MAY2019 thru 15MAY2019]**	1.166	0.569	2.389	0.6671
**[16MAY2020 thru 31MAY2020] vs [16MAY2019 thru 31MAY2019]**	1.316	0.661	2.619	0.4255
**[01JUN2020 thru 15JUN2020] vs [01JUN2019 thru 15JUN2019]**	1.469	0.711	3.035	0.2907
**[16JUN2020 thru 30JUN2020] vs [16JUN2019 thru 30JUN2019]**	1.589	0.738	3.420	0.2301
**[01JUL2020 thru 15JUL2020] vs [01JUL2019 thru 15JUL2019]**	1.638	0.776	3.460	0.19
**[16JUL2020 thru 31JUL2020] vs [16JUL2019 thru 31JUL2019]**	1.630	0.819	3.245	0.1597
**[01AUG2020 thru 15AUG2020] vs [01AUG2019 thru 15AUG2019]**	1.593	0.833	3.046	0.1547
**[16AUG2020 thru 31AUG2020] vs [16AUG2019 thru 31AUG2019]**	1.557	0.801	3.030	0.1862
**[01SEP2020 thru 15SEP2020] vs [01SEP2019 thru 15SEP2019]**	1.550	0.761	3.159	0.2209
**[16SEP2020 thru 30SEP2020] vs [16SEP2019 thru 30SEP2019]**	1.602	0.760	3.375	0.2088
**[01OCT2020 thru 15OCT2020] vs [01OCT2019 thru 15OCT2019]**	1.744	0.861	3.533	0.1196
**[16OCT2020 thru 31OCT2020] vs [16OCT2019 thru 31OCT2019]**	1.929	1.013	3.671	0.0457
**[01NOV2020 thru 15NOV2020] vs [01NOV2019 thru 15NOV2019]**	2.142	1.161	3.952	0.016
**[16NOV2020 thru 30NOV2020] vs [16NOV2019 thru 30NOV2019]**	2.312	1.219	4.385	0.0115
**[01DEC2020 thru 15DEC2020] vs [01DEC2019 thru 15DEC2019]**	2.356	1.181	4.703	0.0163
**[16DEC2020 thru 31DEC2020] vs [16DEC2019 thru 31DEC2019]**	2.206	1.082	4.499	0.0303
**[01JAN2021 thru 15JAN2021] vs [01JAN2020 thru 15JAN2020]**	1.880	0.931	3.794	0.0768
**[16JAN2021 thru 31JAN2021] vs [16JAN2020 thru 31JAN2020]**	1.553	0.752	3.206	0.2276
**[01FEB2021 thru 15FEB2021] vs [01FEB2020 thru 15FEB2020]**	1.349	0.619	2.939	0.4424
**[16FEB2021 thru 28FEB2021] vs [16FEB2020 thru 29FEB2020]**	1.337	0.604	2.960	0.4645
**[01MAR2020 thru 31MAR2021] vs [01FEB2019 thru 29FEB2020]**	1.589	1.147	2.203	0.0065

Nebraska
Bimonthly Data

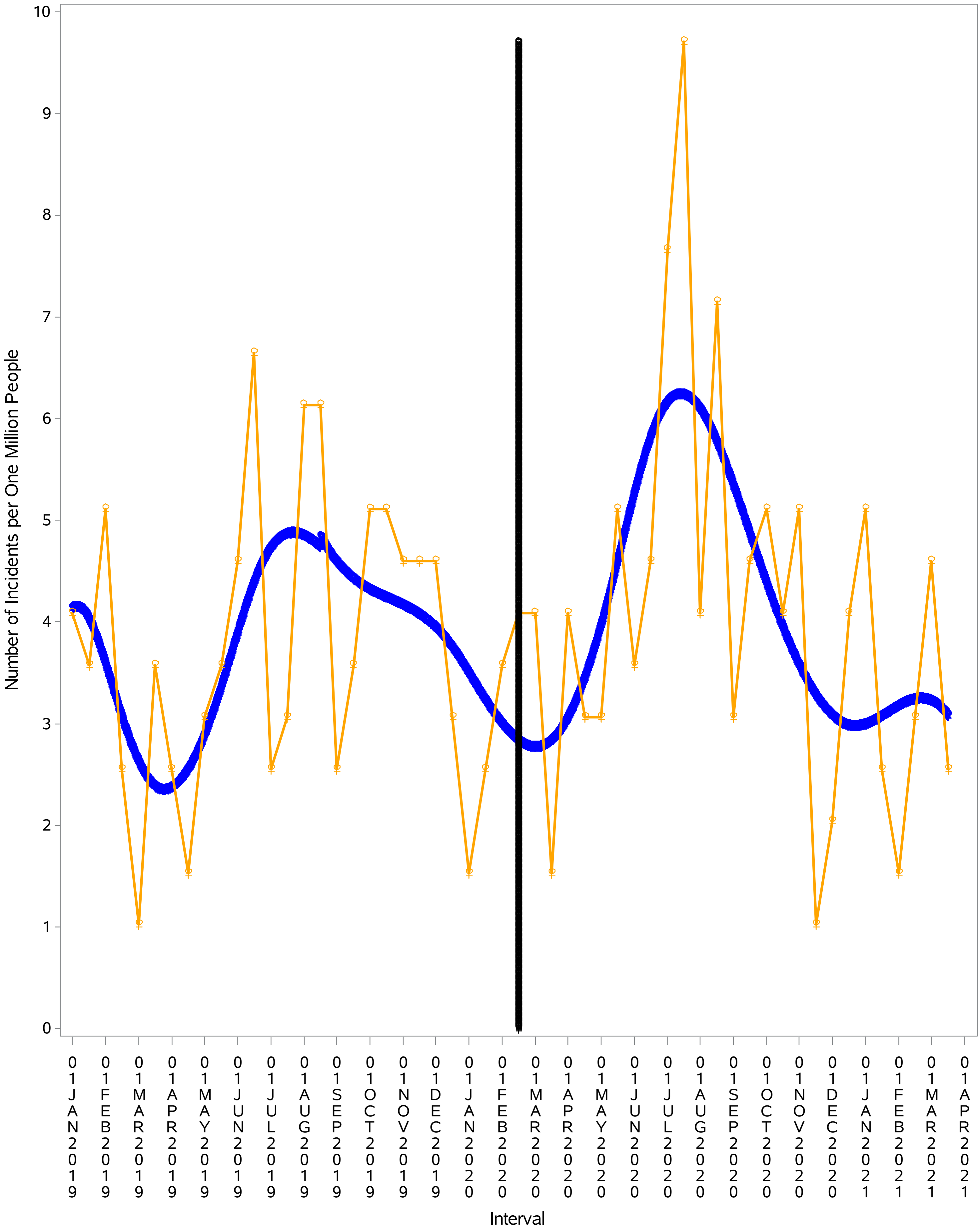

Nebraska
Bimonthly Data

Comparison	IntensityRatio	IntensityRatio_LowerCL	IntensityRatio_UpperCL	P_Value
**[01MAR2020 thru 15MAR2020] vs [01MAR2019 thru 15MAR2019]**	1.056	0.616	1.811	0.8391
**[16MAR2020 thru 31MAR2020] vs [16MAR2019 thru 31MAR2019]**	1.190	0.677	2.092	0.538
**[01APR2020 thru 15APR2020] vs [01APR2019 thru 15APR2019]**	1.291	0.729	2.285	0.3719
**[16APR2020 thru 30APR2020] vs [16APR2019 thru 30APR2019]**	1.351	0.796	2.292	0.2579
**[01MAY2020 thru 15MAY2020] vs [01MAY2019 thru 15MAY2019]**	1.374	0.862	2.191	0.1768
**[16MAY2020 thru 31MAY2020] vs [16MAY2019 thru 31MAY2019]**	1.370	0.894	2.099	0.1444
**[01JUN2020 thru 15JUN2020] vs [01JUN2019 thru 15JUN2019]**	1.349	0.879	2.070	0.1653
**[16JUN2020 thru 30JUN2020] vs [16JUN2019 thru 30JUN2019]**	1.324	0.853	2.056	0.2049
**[01JUL2020 thru 15JUL2020] vs [01JUL2019 thru 15JUL2019]**	1.303	0.854	1.988	0.2139
**[16JUL2020 thru 31JUL2020] vs [16JUL2019 thru 31JUL2019]**	1.281	0.870	1.887	0.2028
**[01AUG2020 thru 15AUG2020] vs [01AUG2019 thru 15AUG2019]**	1.255	0.869	1.813	0.2196
**[16AUG2020 thru 31AUG2020] vs [16AUG2019 thru 31AUG2019]**	1.218	0.828	1.793	0.3085
**[01SEP2020 thru 15SEP2020] vs [01SEP2019 thru 15SEP2019]**	1.164	0.764	1.774	0.4717
**[16SEP2020 thru 30SEP2020] vs [16SEP2019 thru 30SEP2019]**	1.098	0.704	1.714	0.6724
**[01OCT2020 thru 15OCT2020] vs [01OCT2019 thru 15OCT2019]**	1.013	0.659	1.556	0.9522
**[16OCT2020 thru 31OCT2020] vs [16OCT2019 thru 31OCT2019]**	0.929	0.614	1.405	0.7213
**[01NOV2020 thru 15NOV2020] vs [01NOV2019 thru 15NOV2019]**	0.856	0.557	1.314	0.4681
**[16NOV2020 thru 30NOV2020] vs [16NOV2019 thru 30NOV2019]**	0.804	0.501	1.292	0.3587
**[01DEC2020 thru 15DEC2020] vs [01DEC2019 thru 15DEC2019]**	0.782	0.468	1.306	0.3386
**[16DEC2020 thru 31DEC2020] vs [16DEC2019 thru 31DEC2019]**	0.797	0.475	1.337	0.3804
**[01JAN2021 thru 15JAN2021] vs [01JAN2020 thru 15JAN2020]**	0.857	0.522	1.408	0.5347
**[16JAN2021 thru 31JAN2021] vs [16JAN2020 thru 31JAN2020]**	0.952	0.572	1.585	0.8464
**[01FEB2021 thru 15FEB2021] vs [01FEB2020 thru 15FEB2020]**	1.058	0.611	1.835	0.8362
**[16FEB2021 thru 28FEB2021] vs [16FEB2020 thru 29FEB2020]**	1.143	0.654	1.999	0.6316
**[01MAR2020 thru 31MAR2021] vs [01FEB2019 thru 29FEB2020]**	1.080	0.876	1.332	0.462

Nevada
Bimonthly Data

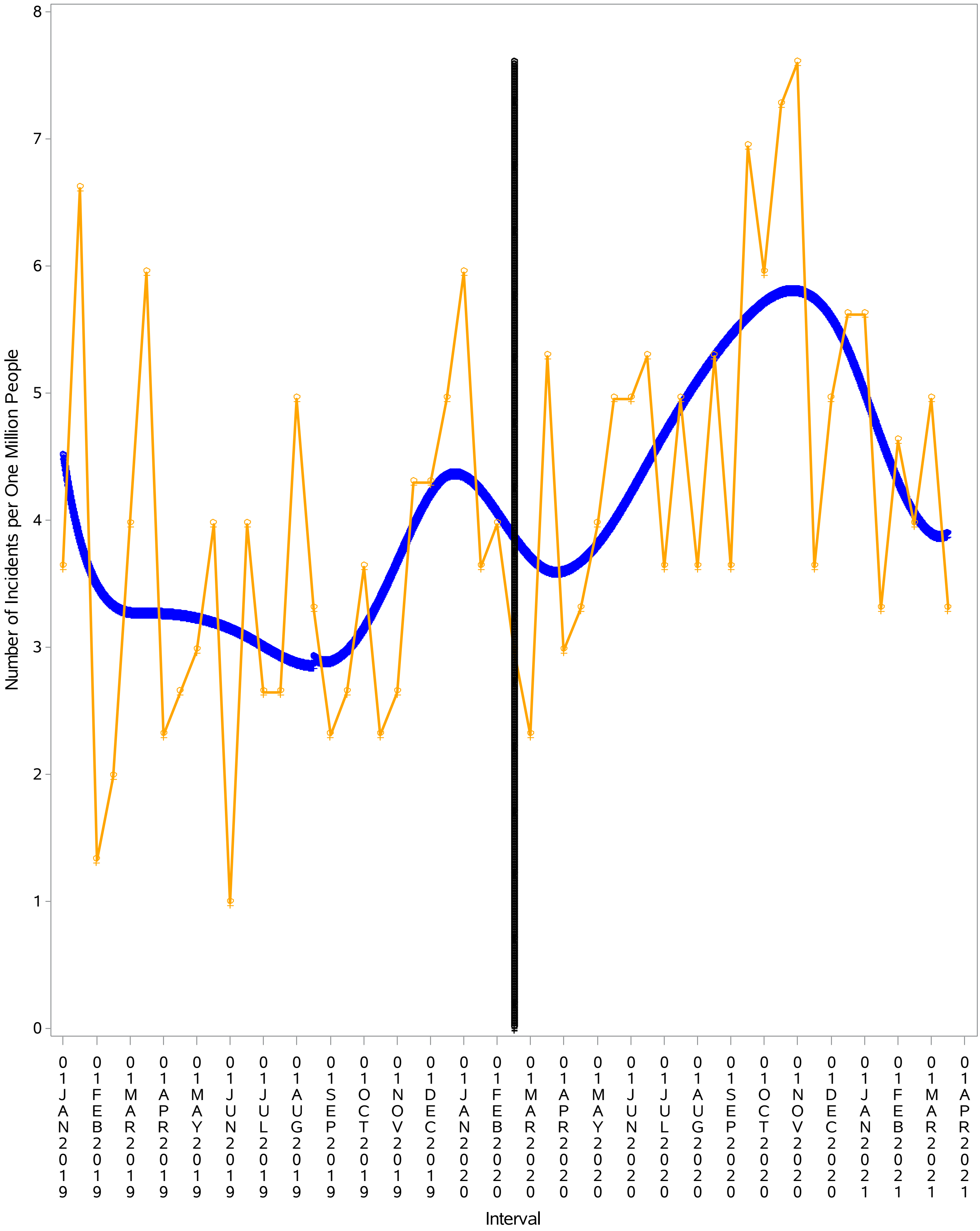

Nevada
Bimonthly Data

Comparison	IntensityRatio	IntensityRatio_LowerCL	IntensityRatio_UpperCL	P_Value
**[01MAR2020 thru 15MAR2020] vs [01MAR2019 thru 15MAR2019]**	1.131	0.726	1.761	0.5783
**[16MAR2020 thru 31MAR2020] vs [16MAR2019 thru 31MAR2019]**	1.103	0.697	1.746	0.6687
**[01APR2020 thru 15APR2020] vs [01APR2019 thru 15APR2019]**	1.102	0.691	1.757	0.6763
**[16APR2020 thru 30APR2020] vs [16APR2019 thru 30APR2019]**	1.128	0.728	1.749	0.5807
**[01MAY2020 thru 15MAY2020] vs [01MAY2019 thru 15MAY2019]**	1.179	0.788	1.763	0.4148
**[16MAY2020 thru 31MAY2020] vs [16MAY2019 thru 31MAY2019]**	1.250	0.840	1.858	0.2632
**[01JUN2020 thru 15JUN2020] vs [01JUN2019 thru 15JUN2019]**	1.339	0.877	2.045	0.1718
**[16JUN2020 thru 30JUN2020] vs [16JUN2019 thru 30JUN2019]**	1.442	0.922	2.257	0.106
**[01JUL2020 thru 15JUL2020] vs [01JUL2019 thru 15JUL2019]**	1.555	1.006	2.403	0.0468
**[16JUL2020 thru 31JUL2020] vs [16JUL2019 thru 31JUL2019]**	1.669	1.119	2.489	0.0133
**[01AUG2020 thru 15AUG2020] vs [01AUG2019 thru 15AUG2019]**	1.772	1.218	2.578	0.0037
**[16AUG2020 thru 31AUG2020] vs [16AUG2019 thru 31AUG2019]**	1.851	1.263	2.715	0.0023
**[01SEP2020 thru 15SEP2020] vs [01SEP2019 thru 15SEP2019]**	1.888	1.256	2.837	0.003
**[16SEP2020 thru 30SEP2020] vs [16SEP2019 thru 30SEP2019]**	1.883	1.230	2.881	0.0045
**[01OCT2020 thru 15OCT2020] vs [01OCT2019 thru 15OCT2019]**	1.817	1.213	2.721	0.0047
**[16OCT2020 thru 31OCT2020] vs [16OCT2019 thru 31OCT2019]**	1.709	1.181	2.472	0.0055
**[01NOV2020 thru 15NOV2020] vs [01NOV2019 thru 15NOV2019]**	1.581	1.111	2.248	0.0121
**[16NOV2020 thru 30NOV2020] vs [16NOV2019 thru 30NOV2019]**	1.450	1.005	2.091	0.0472
**[01DEC2020 thru 15DEC2020] vs [01DEC2019 thru 15DEC2019]**	1.329	0.899	1.963	0.1495
**[16DEC2020 thru 31DEC2020] vs [16DEC2019 thru 31DEC2019]**	1.227	0.826	1.822	0.3024
**[01JAN2021 thru 15JAN2021] vs [01JAN2020 thru 15JAN2020]**	1.149	0.784	1.686	0.4672
**[16JAN2021 thru 31JAN2021] vs [16JAN2020 thru 31JAN2020]**	1.095	0.737	1.626	0.6467
**[01FEB2021 thru 15FEB2021] vs [01FEB2020 thru 15FEB2020]**	1.061	0.692	1.627	0.7821
**[16FEB2021 thru 28FEB2021] vs [16FEB2020 thru 29FEB2020]**	1.047	0.675	1.622	0.835
**[01MAR2020 thru 31MAR2021] vs [01FEB2019 thru 29FEB2020]**	1.357	1.130	1.631	0.0017

New Hampshire
Bimonthly Data

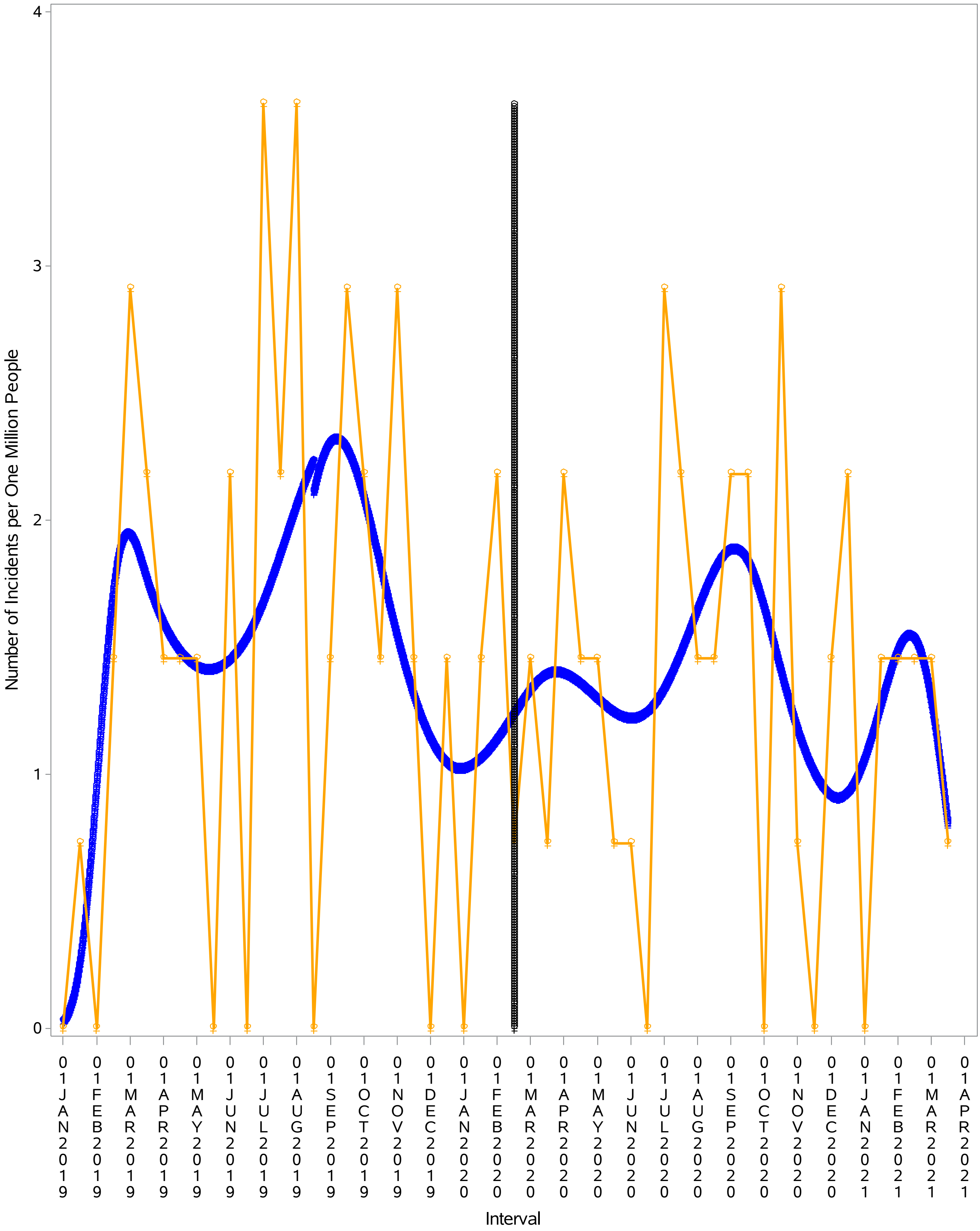

New Hampshire
Bimonthly Data

Comparison	IntensityRatio	IntensityRatio_LowerCL	IntensityRatio_UpperCL	P_Value
**[01MAR2020 thru 15MAR2020] vs [01MAR2019 thru 15MAR2019]**	0.686	0.269	1.750	0.4209
**[16MAR2020 thru 31MAR2020] vs [16MAR2019 thru 31MAR2019]**	0.788	0.324	1.920	0.5923
**[01APR2020 thru 15APR2020] vs [01APR2019 thru 15APR2019]**	0.876	0.354	2.172	0.7706
**[16APR2020 thru 30APR2020] vs [16APR2019 thru 30APR2019]**	0.914	0.381	2.195	0.8369
**[01MAY2020 thru 15MAY2020] vs [01MAY2019 thru 15MAY2019]**	0.910	0.400	2.069	0.8182
**[16MAY2020 thru 31MAY2020] vs [16MAY2019 thru 31MAY2019]**	0.881	0.392	1.978	0.7526
**[01JUN2020 thru 15JUN2020] vs [01JUN2019 thru 15JUN2019]**	0.842	0.359	1.976	0.6865
**[16JUN2020 thru 30JUN2020] vs [16JUN2019 thru 30JUN2019]**	0.810	0.333	1.972	0.6359
**[01JUL2020 thru 15JUL2020] vs [01JUL2019 thru 15JUL2019]**	0.795	0.339	1.865	0.5904
**[16JUL2020 thru 31JUL2020] vs [16JUL2019 thru 31JUL2019]**	0.793	0.368	1.710	0.5466
**[01AUG2020 thru 15AUG2020] vs [01AUG2019 thru 15AUG2019]**	0.799	0.395	1.619	0.5253
**[16AUG2020 thru 31AUG2020] vs [16AUG2019 thru 31AUG2019]**	0.807	0.396	1.648	0.5482
**[01SEP2020 thru 15SEP2020] vs [01SEP2019 thru 15SEP2019]**	0.818	0.382	1.752	0.5971
**[16SEP2020 thru 30SEP2020] vs [16SEP2019 thru 30SEP2019]**	0.807	0.364	1.791	0.5908
**[01OCT2020 thru 15OCT2020] vs [01OCT2019 thru 15OCT2019]**	0.786	0.363	1.700	0.5318
**[16OCT2020 thru 31OCT2020] vs [16OCT2019 thru 31OCT2019]**	0.772	0.361	1.652	0.4958
**[01NOV2020 thru 15NOV2020] vs [01NOV2019 thru 15NOV2019]**	0.760	0.335	1.728	0.5043
**[16NOV2020 thru 30NOV2020] vs [16NOV2019 thru 30NOV2019]**	0.767	0.302	1.950	0.5696
**[01DEC2020 thru 15DEC2020] vs [01DEC2019 thru 15DEC2019]**	0.804	0.289	2.235	0.6693
**[16DEC2020 thru 31DEC2020] vs [16DEC2019 thru 31DEC2019]**	0.888	0.318	2.477	0.8162
**[01JAN2021 thru 15JAN2021] vs [01JAN2020 thru 15JAN2020]**	1.033	0.396	2.693	0.9455
**[16JAN2021 thru 31JAN2021] vs [16JAN2020 thru 31JAN2020]**	1.201	0.471	3.062	0.6943
**[01FEB2021 thru 15FEB2021] vs [01FEB2020 thru 15FEB2020]**	1.306	0.496	3.440	0.5813
**[16FEB2021 thru 28FEB2021] vs [16FEB2020 thru 29FEB2020]**	1.241	0.471	3.269	0.6551
**[01MAR2020 thru 31MAR2021] vs [01FEB2019 thru 29FEB2020]**	0.848	0.572	1.257	0.402

New Jersey
Bimonthly Data

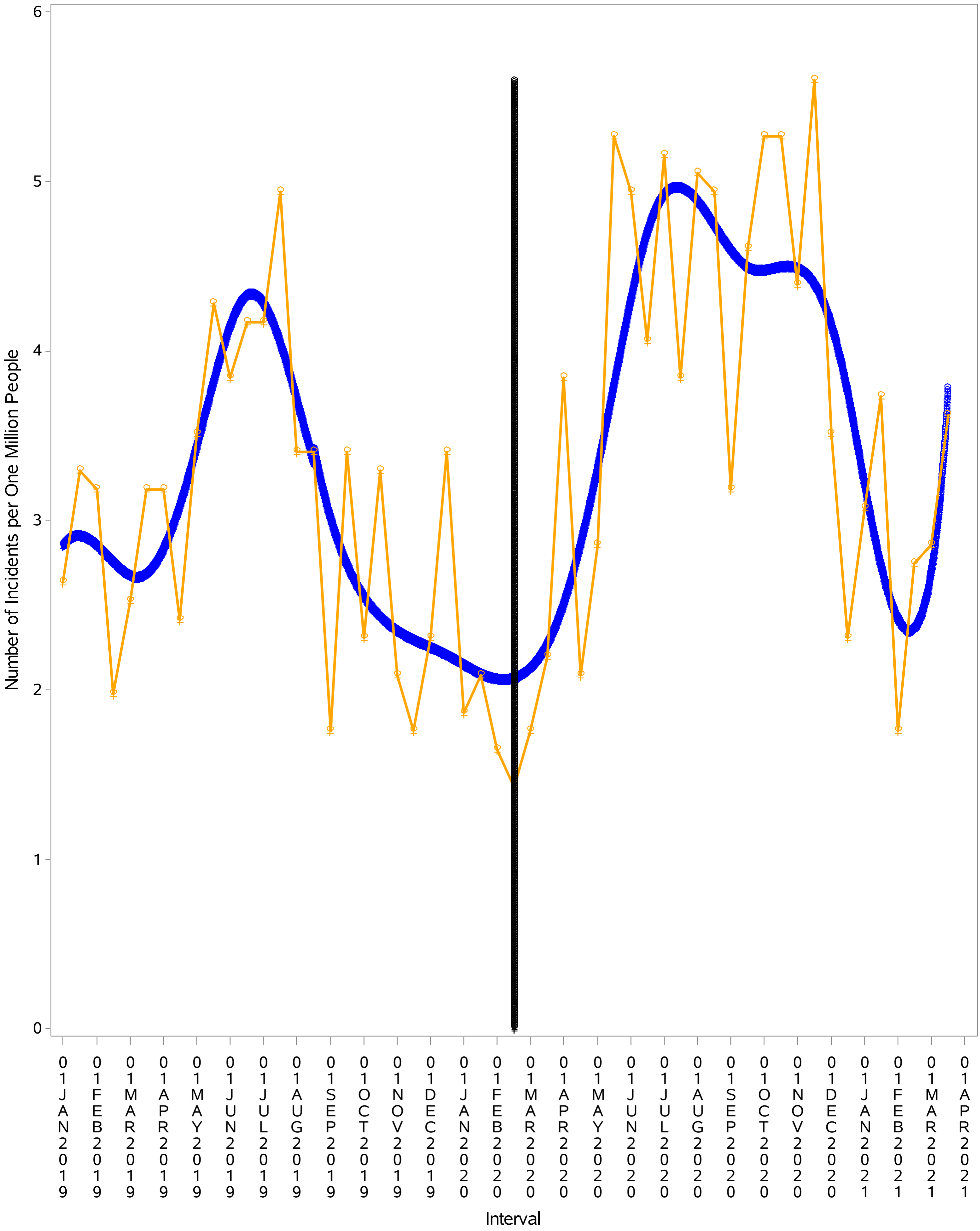

New Jersey
Bimonthly Data

Comparison	IntensityRatio	IntensityRatio_LowerCL	IntensityRatio_UpperCL	P_Value
**[01MAR2020 thru 15MAR2020] vs [01MAR2019 thru 15MAR2019]**	0.796	0.573	1.106	0.1684
**[16MAR2020 thru 31MAR2020] vs [16MAR2019 thru 31MAR2019]**	0.845	0.606	1.178	0.3129
**[01APR2020 thru 15APR2020] vs [01APR2019 thru 15APR2019]**	0.889	0.639	1.237	0.476
**[16APR2020 thru 30APR2020] vs [16APR2019 thru 30APR2019]**	0.927	0.685	1.255	0.6162
**[01MAY2020 thru 15MAY2020] vs [01MAY2019 thru 15MAY2019]**	0.962	0.736	1.257	0.7714
**[16MAY2020 thru 31MAY2020] vs [16MAY2019 thru 31MAY2019]**	0.998	0.777	1.280	0.9841
**[01JUN2020 thru 15JUN2020] vs [01JUN2019 thru 15JUN2019]**	1.038	0.803	1.341	0.7721
**[16JUN2020 thru 30JUN2020] vs [16JUN2019 thru 30JUN2019]**	1.087	0.832	1.420	0.5307
**[01JUL2020 thru 15JUL2020] vs [01JUL2019 thru 15JUL2019]**	1.151	0.889	1.490	0.2792
**[16JUL2020 thru 31JUL2020] vs [16JUL2019 thru 31JUL2019]**	1.228	0.967	1.561	0.0904
**[01AUG2020 thru 15AUG2020] vs [01AUG2019 thru 15AUG2019]**	1.319	1.045	1.665	0.0211
**[16AUG2020 thru 31AUG2020] vs [16AUG2019 thru 31AUG2019]**	1.420	1.106	1.824	0.0072
**[01SEP2020 thru 15SEP2020] vs [01SEP2019 thru 15SEP2019]**	1.525	1.158	2.008	0.0035
**[16SEP2020 thru 30SEP2020] vs [16SEP2019 thru 30SEP2019]**	1.644	1.229	2.200	0.0013
**[01OCT2020 thru 15OCT2020] vs [01OCT2019 thru 15OCT2019]**	1.761	1.331	2.329	0.0002
**[16OCT2020 thru 31OCT2020] vs [16OCT2019 thru 31OCT2019]**	1.852	1.423	2.411	< 0.0001
**[01NOV2020 thru 15NOV2020] vs [01NOV2019 thru 15NOV2019]**	1.911	1.466	2.491	< 0.0001
**[16NOV2020 thru 30NOV2020] vs [16NOV2019 thru 30NOV2019]**	1.914	1.436	2.551	< 0.0001
**[01DEC2020 thru 15DEC2020] vs [01DEC2019 thru 15DEC2019]**	1.844	1.350	2.521	0.0003
**[16DEC2020 thru 31DEC2020] vs [16DEC2019 thru 31DEC2019]**	1.697	1.232	2.338	0.0018
**[01JAN2021 thru 15JAN2021] vs [01JAN2020 thru 15JAN2020]**	1.491	1.087	2.045	0.0144
**[16JAN2021 thru 31JAN2021] vs [16JAN2020 thru 31JAN2020]**	1.298	0.935	1.802	0.1166
**[01FEB2021 thru 15FEB2021] vs [01FEB2020 thru 15FEB2020]**	1.170	0.823	1.665	0.3734
**[16FEB2021 thru 28FEB2021] vs [16FEB2020 thru 29FEB2020]**	1.144	0.801	1.632	0.4506
**[01MAR2020 thru 31MAR2021] vs [01FEB2019 thru 29FEB2020]**	1.266	1.112	1.442	0.0007

New Mexico
Bimonthly Data

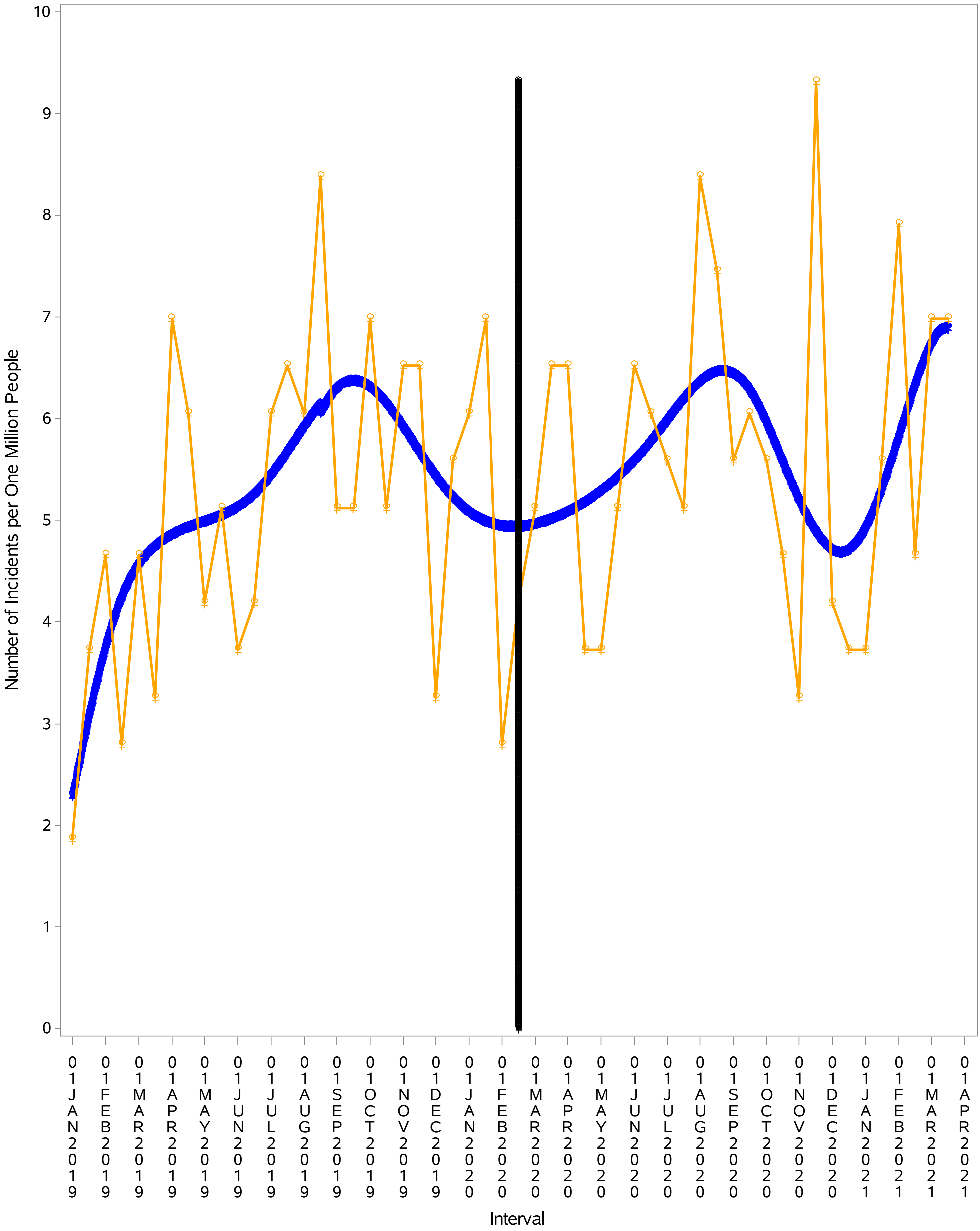

New Mexico
Bimonthly Data

Comparison	IntensityRatio	IntensityRatio_LowerCL	IntensityRatio_UpperCL	P_Value
**[01MAR2020 thru 15MAR2020] vs [01MAR2019 thru 15MAR2019]**	1.087	0.741	1.595	0.6631
**[16MAR2020 thru 31MAR2020] vs [16MAR2019 thru 31MAR2019]**	1.057	0.724	1.543	0.7702
**[01APR2020 thru 15APR2020] vs [01APR2019 thru 15APR2019]**	1.047	0.714	1.534	0.8101
**[16APR2020 thru 30APR2020] vs [16APR2019 thru 30APR2019]**	1.049	0.732	1.504	0.789
**[01MAY2020 thru 15MAY2020] vs [01MAY2019 thru 15MAY2019]**	1.060	0.761	1.475	0.7252
**[16MAY2020 thru 31MAY2020] vs [16MAY2019 thru 31MAY2019]**	1.074	0.778	1.482	0.6561
**[01JUN2020 thru 15JUN2020] vs [01JUN2019 thru 15JUN2019]**	1.088	0.775	1.528	0.6169
**[16JUN2020 thru 30JUN2020] vs [16JUN2019 thru 30JUN2019]**	1.098	0.770	1.567	0.5982
**[01JUL2020 thru 15JUL2020] vs [01JUL2019 thru 15JUL2019]**	1.099	0.780	1.549	0.5812
**[16JUL2020 thru 31JUL2020] vs [16JUL2019 thru 31JUL2019]**	1.091	0.798	1.494	0.5765
**[01AUG2020 thru 15AUG2020] vs [01AUG2019 thru 15AUG2019]**	1.075	0.801	1.443	0.6207
**[16AUG2020 thru 31AUG2020] vs [16AUG2019 thru 31AUG2019]**	1.052	0.778	1.422	0.7386
**[01SEP2020 thru 15SEP2020] vs [01SEP2019 thru 15SEP2019]**	1.023	0.740	1.414	0.889
**[16SEP2020 thru 30SEP2020] vs [16SEP2019 thru 30SEP2019]**	0.984	0.702	1.381	0.9252
**[01OCT2020 thru 15OCT2020] vs [01OCT2019 thru 15OCT2019]**	0.942	0.681	1.302	0.7109
**[16OCT2020 thru 31OCT2020] vs [16OCT2019 thru 31OCT2019]**	0.906	0.667	1.231	0.5198
**[01NOV2020 thru 15NOV2020] vs [01NOV2019 thru 15NOV2019]**	0.877	0.642	1.198	0.4004
**[16NOV2020 thru 30NOV2020] vs [16NOV2019 thru 30NOV2019]**	0.863	0.613	1.213	0.3866
**[01DEC2020 thru 15DEC2020] vs [01DEC2019 thru 15DEC2019]**	0.868	0.600	1.257	0.446
**[16DEC2020 thru 31DEC2020] vs [16DEC2019 thru 31DEC2019]**	0.902	0.622	1.309	0.579
**[01JAN2021 thru 15JAN2021] vs [01JAN2020 thru 15JAN2020]**	0.970	0.683	1.376	0.8594
**[16JAN2021 thru 31JAN2021] vs [16JAN2020 thru 31JAN2020]**	1.065	0.754	1.505	0.7153
**[01FEB2021 thru 15FEB2021] vs [01FEB2020 thru 15FEB2020]**	1.175	0.816	1.692	0.3761
**[16FEB2021 thru 28FEB2021] vs [16FEB2020 thru 29FEB2020]**	1.282	0.886	1.854	0.182
**[01MAR2020 thru 31MAR2021] vs [01FEB2019 thru 29FEB2020]**	1.066	0.914	1.243	0.4098

New York
Bimonthly Data

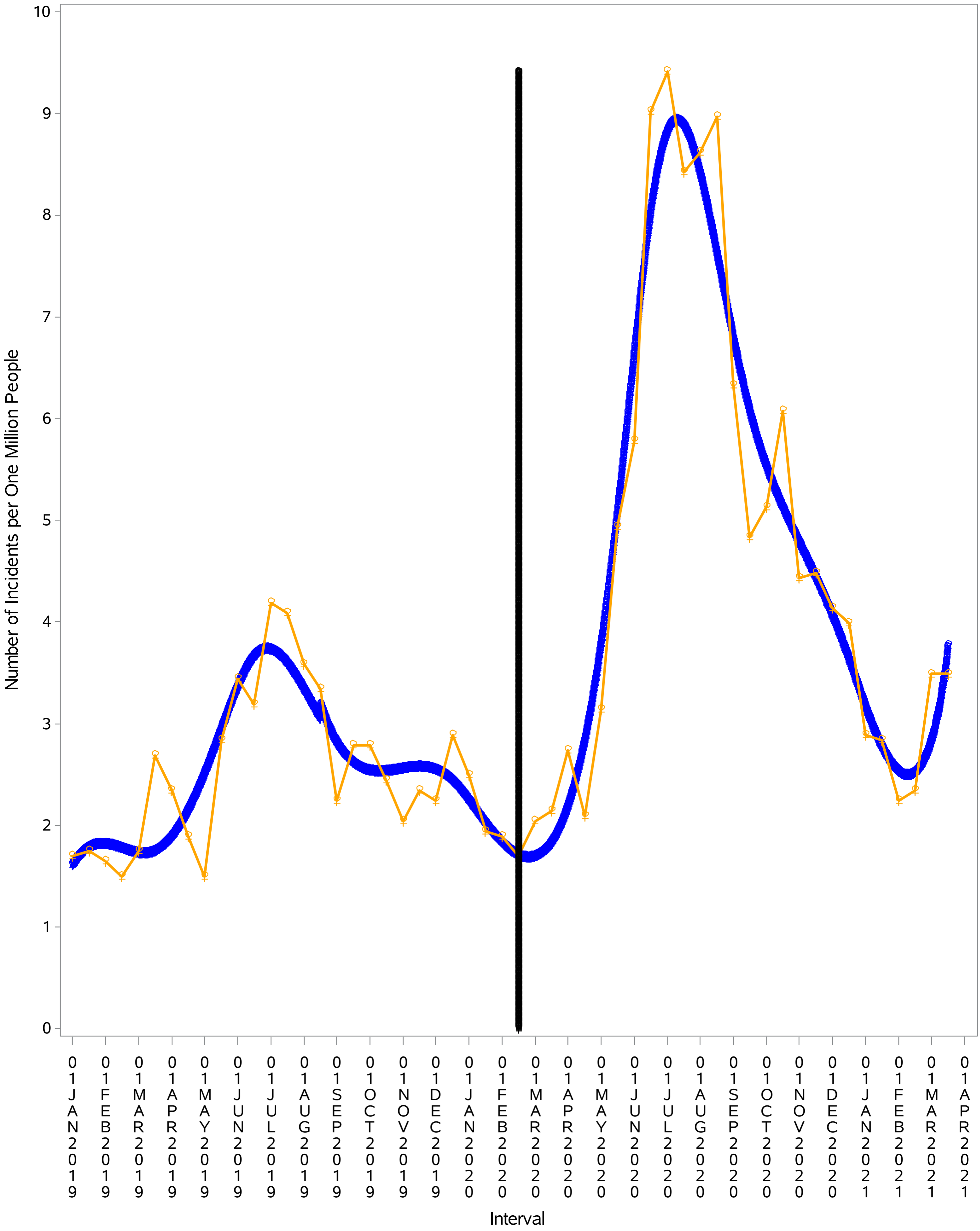

New York
Bimonthly Data

Comparison	IntensityRatio	IntensityRatio_LowerCL	IntensityRatio_UpperCL	P_Value
**[01MAR2020 thru 15MAR2020] vs [01MAR2019 thru 15MAR2019]**	0.981	0.753	1.277	0.8824
**[16MAR2020 thru 31MAR2020] vs [16MAR2019 thru 31MAR2019]**	1.049	0.807	1.363	0.7144
**[01APR2020 thru 15APR2020] vs [01APR2019 thru 15APR2019]**	1.162	0.895	1.508	0.252
**[16APR2020 thru 30APR2020] vs [16APR2019 thru 30APR2019]**	1.317	1.038	1.673	0.0246
**[01MAY2020 thru 15MAY2020] vs [01MAY2019 thru 15MAY2019]**	1.512	1.232	1.855	0.0002
**[16MAY2020 thru 31MAY2020] vs [16MAY2019 thru 31MAY2019]**	1.736	1.451	2.077	< 0.0001
**[01JUN2020 thru 15JUN2020] vs [01JUN2019 thru 15JUN2019]**	1.972	1.655	2.351	< 0.0001
**[16JUN2020 thru 30JUN2020] vs [16JUN2019 thru 30JUN2019]**	2.192	1.829	2.628	< 0.0001
**[01JUL2020 thru 15JUL2020] vs [01JUL2019 thru 15JUL2019]**	2.363	1.982	2.816	< 0.0001
**[16JUL2020 thru 31JUL2020] vs [16JUL2019 thru 31JUL2019]**	2.471	2.101	2.905	< 0.0001
**[01AUG2020 thru 15AUG2020] vs [01AUG2019 thru 15AUG2019]**	2.514	2.151	2.938	< 0.0001
**[16AUG2020 thru 31AUG2020] vs [16AUG2019 thru 31AUG2019]**	2.495	2.113	2.948	< 0.0001
**[01SEP2020 thru 15SEP2020] vs [01SEP2019 thru 15SEP2019]**	2.411	2.007	2.896	< 0.0001
**[16SEP2020 thru 30SEP2020] vs [16SEP2019 thru 30SEP2019]**	2.312	1.901	2.813	< 0.0001
**[01OCT2020 thru 15OCT2020] vs [01OCT2019 thru 15OCT2019]**	2.175	1.799	2.629	< 0.0001
**[16OCT2020 thru 31OCT2020] vs [16OCT2019 thru 31OCT2019]**	2.017	1.685	2.414	< 0.0001
**[01NOV2020 thru 15NOV2020] vs [01NOV2019 thru 15NOV2019]**	1.862	1.554	2.231	< 0.0001
**[16NOV2020 thru 30NOV2020] vs [16NOV2019 thru 30NOV2019]**	1.717	1.411	2.090	< 0.0001
**[01DEC2020 thru 15DEC2020] vs [01DEC2019 thru 15DEC2019]**	1.590	1.284	1.968	0.0001
**[16DEC2020 thru 31DEC2020] vs [16DEC2019 thru 31DEC2019]**	1.484	1.191	1.850	0.0008
**[01JAN2021 thru 15JAN2021] vs [01JAN2020 thru 15JAN2020]**	1.407	1.130	1.752	0.0031
**[16JAN2021 thru 31JAN2021] vs [16JAN2020 thru 31JAN2020]**	1.368	1.088	1.721	0.0085
**[01FEB2021 thru 15FEB2021] vs [01FEB2020 thru 15FEB2020]**	1.384	1.082	1.770	0.0108
**[16FEB2021 thru 28FEB2021] vs [16FEB2020 thru 29FEB2020]**	1.473	1.150	1.888	0.003
**[01MAR2020 thru 31MAR2021] vs [01FEB2019 thru 29FEB2020]**	1.722	1.570	1.888	< 0.0001

North Carolina
Bimonthly Data

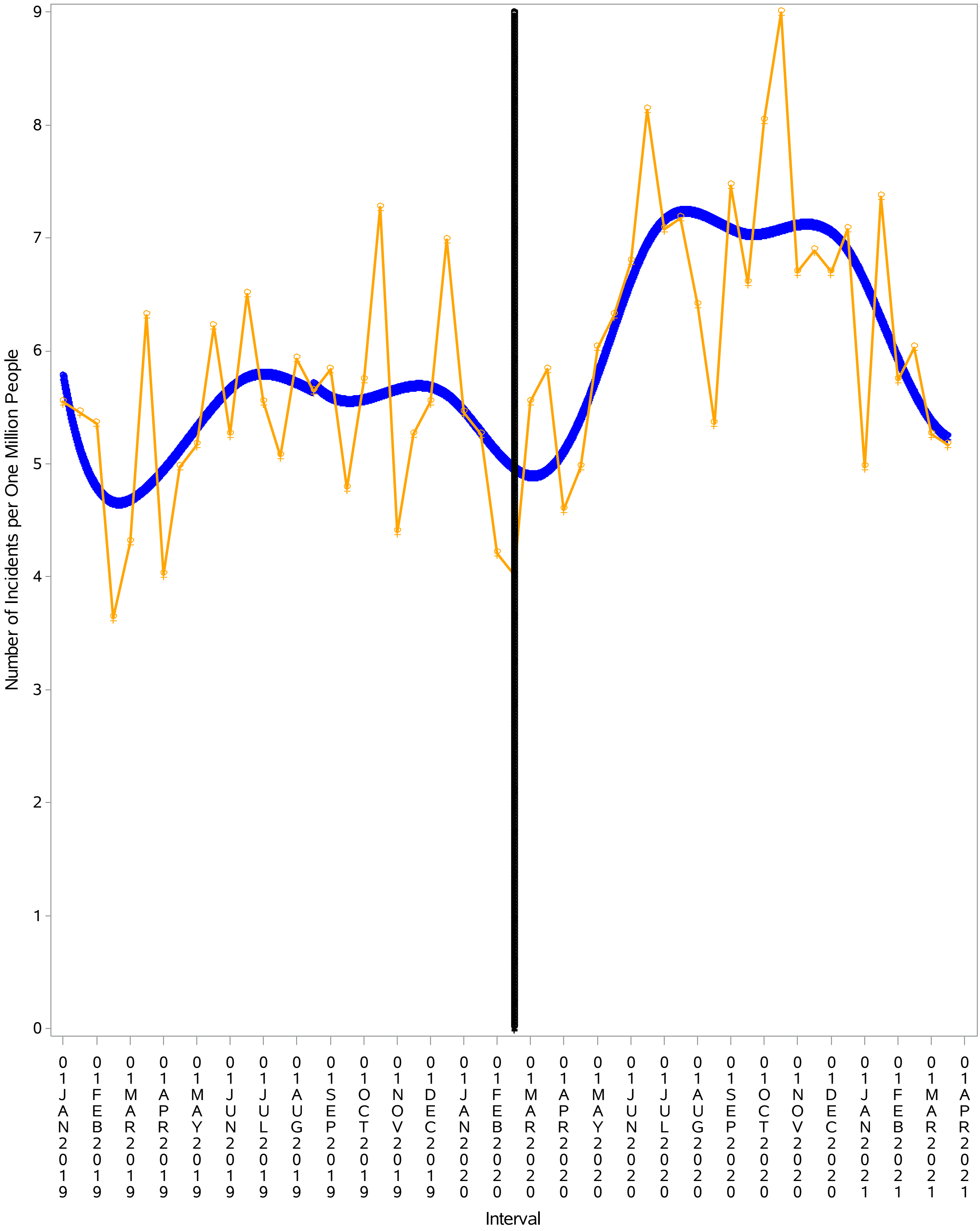

North Carolina
Bimonthly Data

Comparison	IntensityRatio	IntensityRatio_LowerCL	IntensityRatio_UpperCL	P_Value
**[01MAR2020 thru 15MAR2020] vs [01MAR2019 thru 15MAR2019]**	1.046	0.854	1.281	0.6568
**[16MAR2020 thru 31MAR2020] vs [16MAR2019 thru 31MAR2019]**	1.031	0.839	1.268	0.7643
**[01APR2020 thru 15APR2020] vs [01APR2019 thru 15APR2019]**	1.035	0.841	1.273	0.7423
**[16APR2020 thru 30APR2020] vs [16APR2019 thru 30APR2019]**	1.055	0.869	1.280	0.5812
**[01MAY2020 thru 15MAY2020] vs [01MAY2019 thru 15MAY2019]**	1.087	0.912	1.296	0.3427
**[16MAY2020 thru 31MAY2020] vs [16MAY2019 thru 31MAY2019]**	1.127	0.951	1.335	0.1623
**[01JUN2020 thru 15JUN2020] vs [01JUN2019 thru 15JUN2019]**	1.169	0.978	1.396	0.0846
**[16JUN2020 thru 30JUN2020] vs [16JUN2019 thru 30JUN2019]**	1.206	1.001	1.454	0.0491
**[01JUL2020 thru 15JUL2020] vs [01JUL2019 thru 15JUL2019]**	1.234	1.030	1.479	0.0236
**[16JUL2020 thru 31JUL2020] vs [16JUL2019 thru 31JUL2019]**	1.253	1.061	1.480	0.0091
**[01AUG2020 thru 15AUG2020] vs [01AUG2019 thru 15AUG2019]**	1.263	1.079	1.479	0.0047
**[16AUG2020 thru 31AUG2020] vs [16AUG2019 thru 31AUG2019]**	1.267	1.076	1.493	0.0056
**[01SEP2020 thru 15SEP2020] vs [01SEP2019 thru 15SEP2019]**	1.266	1.061	1.511	0.0101
**[16SEP2020 thru 30SEP2020] vs [16SEP2019 thru 30SEP2019]**	1.266	1.052	1.523	0.0137
**[01OCT2020 thru 15OCT2020] vs [01OCT2019 thru 15OCT2019]**	1.265	1.061	1.509	0.0101
**[16OCT2020 thru 31OCT2020] vs [16OCT2019 thru 31OCT2019]**	1.261	1.071	1.486	0.0065
**[01NOV2020 thru 15NOV2020] vs [01NOV2019 thru 15NOV2019]**	1.257	1.070	1.477	0.0064
**[16NOV2020 thru 30NOV2020] vs [16NOV2019 thru 30NOV2019]**	1.251	1.053	1.486	0.0121
**[01DEC2020 thru 15DEC2020] vs [01DEC2019 thru 15DEC2019]**	1.241	1.031	1.494	0.0233
**[16DEC2020 thru 31DEC2020] vs [16DEC2019 thru 31DEC2019]**	1.228	1.018	1.481	0.0325
**[01JAN2021 thru 15JAN2021] vs [01JAN2020 thru 15JAN2020]**	1.210	1.010	1.450	0.039
**[16JAN2021 thru 31JAN2021] vs [16JAN2020 thru 31JAN2020]**	1.188	0.987	1.430	0.0675
**[01FEB2021 thru 15FEB2021] vs [01FEB2020 thru 15FEB2020]**	1.162	0.952	1.419	0.1369
**[16FEB2021 thru 28FEB2021] vs [16FEB2020 thru 29FEB2020]**	1.132	0.923	1.388	0.2282
**[01MAR2020 thru 31MAR2021] vs [01FEB2019 thru 29FEB2020]**	1.180	1.088	1.280	0.0002

North Dakota
Bimonthly Data

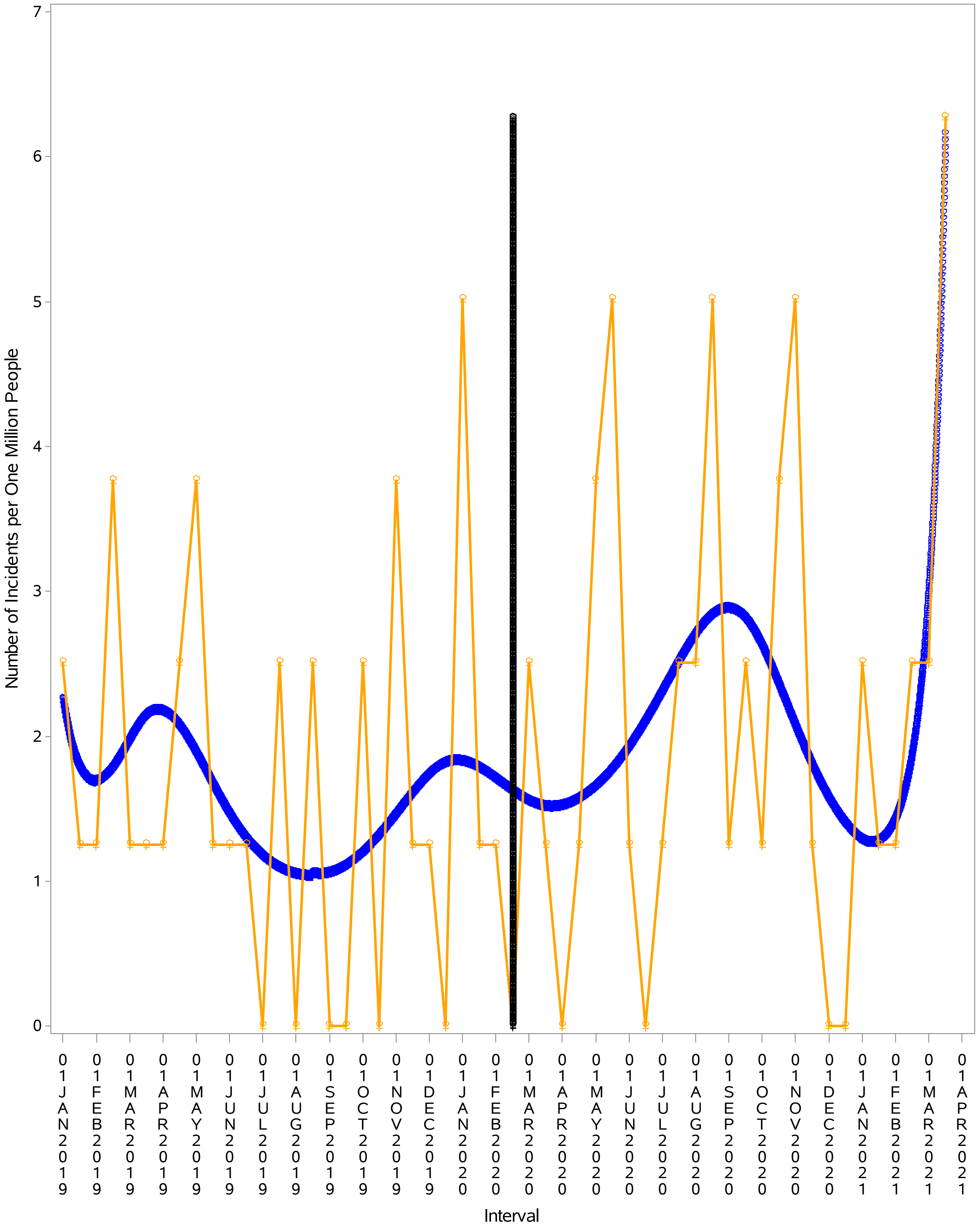

North Dakota
Bimonthly Data

Comparison	IntensityRatio	IntensityRatio_LowerCL	IntensityRatio_UpperCL	P_Value
**[01MAR2020 thru 15MAR2020] vs [01MAR2019 thru 15MAR2019]**	0.789	0.263	2.365	0.6648
**[16MAR2020 thru 31MAR2020] vs [16MAR2019 thru 31MAR2019]**	0.708	0.231	2.175	0.5385
**[01APR2020 thru 15APR2020] vs [01APR2019 thru 15APR2019]**	0.701	0.227	2.162	0.5275
**[16APR2020 thru 30APR2020] vs [16APR2019 thru 30APR2019]**	0.758	0.264	2.179	0.5999
**[01MAY2020 thru 15MAY2020] vs [01MAY2019 thru 15MAY2019]**	0.878	0.327	2.354	0.7914
**[16MAY2020 thru 31MAY2020] vs [16MAY2019 thru 31MAY2019]**	1.063	0.389	2.902	0.9035
**[01JUN2020 thru 15JUN2020] vs [01JUN2019 thru 15JUN2019]**	1.314	0.438	3.946	0.6188
**[16JUN2020 thru 30JUN2020] vs [16JUN2019 thru 30JUN2019]**	1.623	0.503	5.240	0.4089
**[01JUL2020 thru 15JUL2020] vs [01JUL2019 thru 15JUL2019]**	1.962	0.626	6.149	0.2407
**[16JUL2020 thru 31JUL2020] vs [16JUL2019 thru 31JUL2019]**	2.293	0.798	6.588	0.1202
**[01AUG2020 thru 15AUG2020] vs [01AUG2019 thru 15AUG2019]**	2.567	0.944	6.983	0.0642
**[16AUG2020 thru 31AUG2020] vs [16AUG2019 thru 31AUG2019]**	2.727	0.972	7.650	0.0563
**[01SEP2020 thru 15SEP2020] vs [01SEP2019 thru 15SEP2019]**	2.717	0.903	8.177	0.0742
**[16SEP2020 thru 30SEP2020] vs [16SEP2019 thru 30SEP2019]**	2.533	0.801	8.011	0.1109
**[01OCT2020 thru 15OCT2020] vs [01OCT2019 thru 15OCT2019]**	2.180	0.727	6.542	0.1597
**[16OCT2020 thru 31OCT2020] vs [16OCT2019 thru 31OCT2019]**	1.785	0.644	4.947	0.2581
**[01NOV2020 thru 15NOV2020] vs [01NOV2019 thru 15NOV2019]**	1.416	0.522	3.837	0.4854
**[16NOV2020 thru 30NOV2020] vs [16NOV2019 thru 30NOV2019]**	1.120	0.389	3.225	0.8301
**[01DEC2020 thru 15DEC2020] vs [01DEC2019 thru 15DEC2019]**	0.906	0.290	2.828	0.8622
**[16DEC2020 thru 31DEC2020] vs [16DEC2019 thru 31DEC2019]**	0.770	0.242	2.446	0.6504
**[01JAN2021 thru 15JAN2021] vs [01JAN2020 thru 15JAN2020]**	0.705	0.233	2.135	0.5283
**[16JAN2021 thru 31JAN2021] vs [16JAN2020 thru 31JAN2020]**	0.718	0.238	2.169	0.5484
**[01FEB2021 thru 15FEB2021] vs [01FEB2020 thru 15FEB2020]**	0.836	0.261	2.685	0.7589
**[16FEB2021 thru 28FEB2021] vs [16FEB2020 thru 29FEB2020]**	1.150	0.356	3.720	0.8107
**[01MAR2020 thru 31MAR2021] vs [01FEB2019 thru 29FEB2020]**	1.335	0.820	2.172	0.2382

Ohio
Bimonthly Data

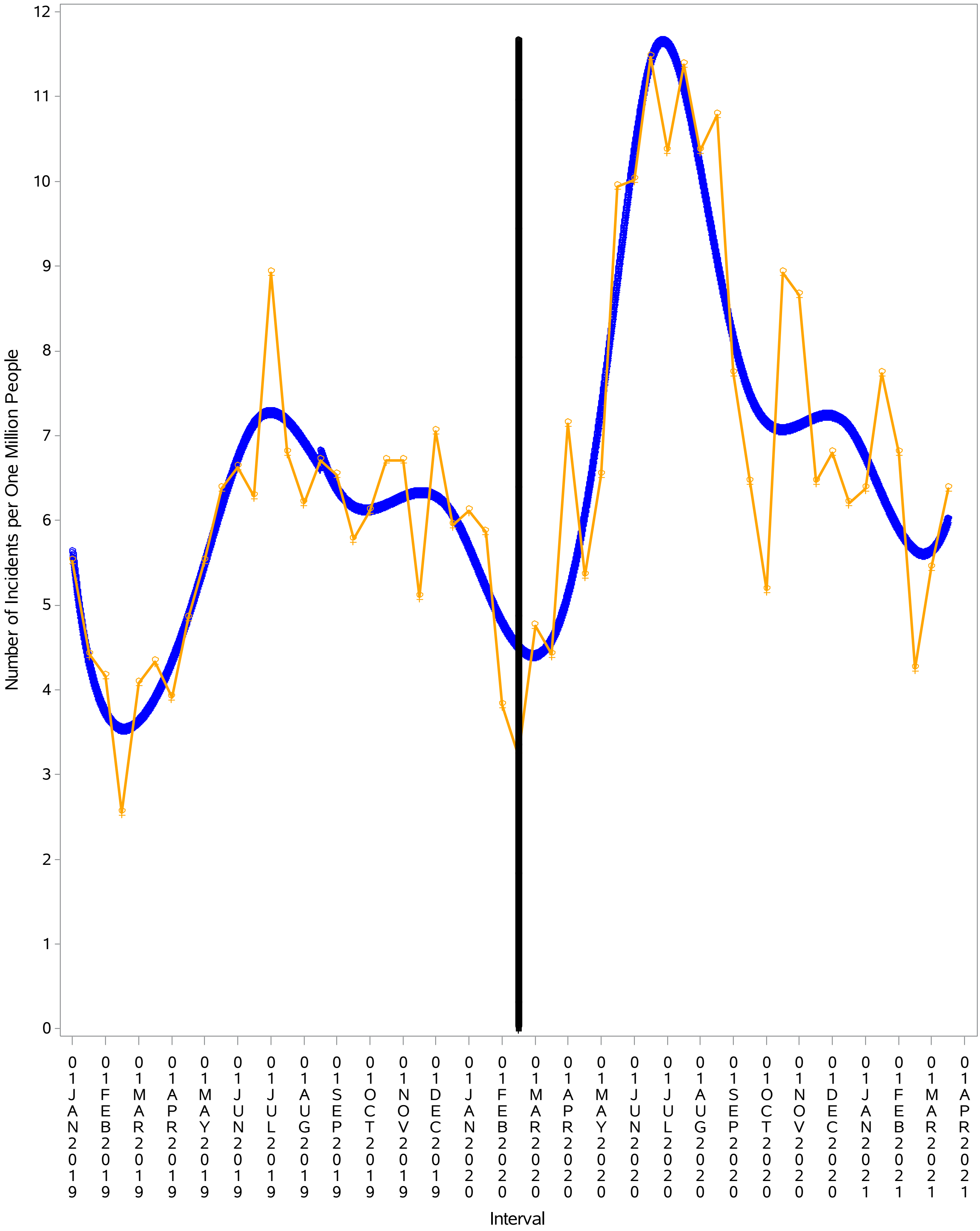

Ohio
Bimonthly Data

Comparison	IntensityRatio	IntensityRatio_LowerCL	IntensityRatio_UpperCL	P_Value
**[01MAR2020 thru 15MAR2020] vs [01MAR2019 thru 15MAR2019]**	1.215	0.963	1.533	0.0986
**[16MAR2020 thru 31MAR2020] vs [16MAR2019 thru 31MAR2019]**	1.175	0.933	1.479	0.1652
**[01APR2020 thru 15APR2020] vs [01APR2019 thru 15APR2019]**	1.187	0.944	1.492	0.1377
**[16APR2020 thru 30APR2020] vs [16APR2019 thru 30APR2019]**	1.246	1.010	1.538	0.0409
**[01MAY2020 thru 15MAY2020] vs [01MAY2019 thru 15MAY2019]**	1.337	1.112	1.607	0.0028
**[16MAY2020 thru 31MAY2020] vs [16MAY2019 thru 31MAY2019]**	1.442	1.219	1.706	0.0001
**[01JUN2020 thru 15JUN2020] vs [01JUN2019 thru 15JUN2019]**	1.539	1.298	1.824	< 0.0001
**[16JUN2020 thru 30JUN2020] vs [16JUN2019 thru 30JUN2019]**	1.599	1.340	1.908	< 0.0001
**[01JUL2020 thru 15JUL2020] vs [01JUL2019 thru 15JUL2019]**	1.598	1.346	1.897	< 0.0001
**[16JUL2020 thru 31JUL2020] vs [16JUL2019 thru 31JUL2019]**	1.546	1.319	1.813	< 0.0001
**[01AUG2020 thru 15AUG2020] vs [01AUG2019 thru 15AUG2019]**	1.464	1.256	1.707	< 0.0001
**[16AUG2020 thru 31AUG2020] vs [16AUG2019 thru 31AUG2019]**	1.371	1.164	1.614	0.0003
**[01SEP2020 thru 15SEP2020] vs [01SEP2019 thru 15SEP2019]**	1.278	1.069	1.528	0.0083
**[16SEP2020 thru 30SEP2020] vs [16SEP2019 thru 30SEP2019]**	1.212	1.004	1.464	0.0457
**[01OCT2020 thru 15OCT2020] vs [01OCT2019 thru 15OCT2019]**	1.170	0.976	1.402	0.0875
**[16OCT2020 thru 31OCT2020] vs [16OCT2019 thru 31OCT2019]**	1.143	0.965	1.355	0.1194
**[01NOV2020 thru 15NOV2020] vs [01NOV2019 thru 15NOV2019]**	1.135	0.959	1.344	0.1369
**[16NOV2020 thru 30NOV2020] vs [16NOV2019 thru 30NOV2019]**	1.140	0.951	1.367	0.1533
**[01DEC2020 thru 15DEC2020] vs [01DEC2019 thru 15DEC2019]**	1.152	0.948	1.401	0.1504
**[16DEC2020 thru 31DEC2020] vs [16DEC2019 thru 31DEC2019]**	1.170	0.960	1.426	0.1173
**[01JAN2021 thru 15JAN2021] vs [01JAN2020 thru 15JAN2020]**	1.189	0.981	1.440	0.0762
**[16JAN2021 thru 31JAN2021] vs [16JAN2020 thru 31JAN2020]**	1.209	0.991	1.475	0.0607
**[01FEB2021 thru 15FEB2021] vs [01FEB2020 thru 15FEB2020]**	1.231	0.993	1.526	0.0579
**[16FEB2021 thru 28FEB2021] vs [16FEB2020 thru 29FEB2020]**	1.254	1.007	1.562	0.0432
**[01MAR2020 thru 31MAR2021] vs [01FEB2019 thru 29FEB2020]**	1.306	1.200	1.422	< 0.0001

Oklahoma
Bimonthly Data

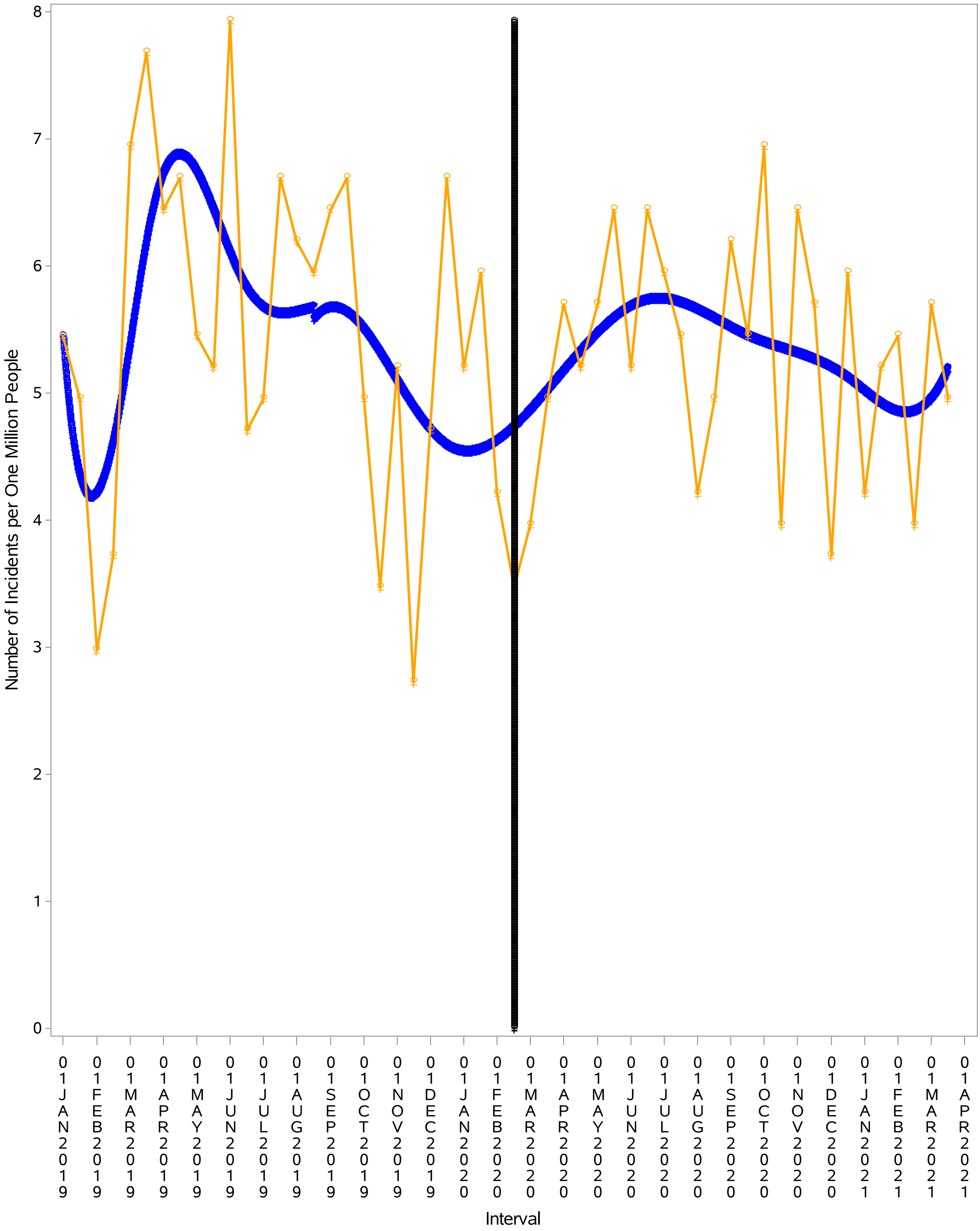

Oklahoma
Bimonthly Data

Comparison	IntensityRatio	IntensityRatio_LowerCL	IntensityRatio_UpperCL	P_Value
**[01MAR2020 thru 15MAR2020] vs [01MAR2019 thru 15MAR2019]**	0.905	0.690	1.189	0.4659
**[16MAR2020 thru 31MAR2020] vs [16MAR2019 thru 31MAR2019]**	0.810	0.620	1.058	0.1187
**[01APR2020 thru 15APR2020] vs [01APR2019 thru 15APR2019]**	0.770	0.590	1.004	0.0532
**[16APR2020 thru 30APR2020] vs [16APR2019 thru 30APR2019]**	0.775	0.605	0.993	0.0441
**[01MAY2020 thru 15MAY2020] vs [01MAY2019 thru 15MAY2019]**	0.812	0.645	1.021	0.0735
**[16MAY2020 thru 31MAY2020] vs [16MAY2019 thru 31MAY2019]**	0.868	0.690	1.092	0.2208
**[01JUN2020 thru 15JUN2020] vs [01JUN2019 thru 15JUN2019]**	0.931	0.727	1.193	0.5657
**[16JUN2020 thru 30JUN2020] vs [16JUN2019 thru 30JUN2019]**	0.985	0.758	1.279	0.9056
**[01JUL2020 thru 15JUL2020] vs [01JUL2019 thru 15JUL2019]**	1.012	0.785	1.305	0.926
**[16JUL2020 thru 31JUL2020] vs [16JUL2019 thru 31JUL2019]**	1.015	0.802	1.284	0.8975
**[01AUG2020 thru 15AUG2020] vs [01AUG2019 thru 15AUG2019]**	1.003	0.801	1.258	0.976
**[16AUG2020 thru 31AUG2020] vs [16AUG2019 thru 31AUG2019]**	0.986	0.778	1.249	0.9023
**[01SEP2020 thru 15SEP2020] vs [01SEP2019 thru 15SEP2019]**	0.973	0.753	1.257	0.8292
**[16SEP2020 thru 30SEP2020] vs [16SEP2019 thru 30SEP2019]**	0.967	0.740	1.264	0.8031
**[01OCT2020 thru 15OCT2020] vs [01OCT2019 thru 15OCT2019]**	0.981	0.761	1.266	0.8822
**[16OCT2020 thru 31OCT2020] vs [16OCT2019 thru 31OCT2019]**	1.009	0.794	1.281	0.9432
**[01NOV2020 thru 15NOV2020] vs [01NOV2019 thru 15NOV2019]**	1.043	0.821	1.326	0.724
**[16NOV2020 thru 30NOV2020] vs [16NOV2019 thru 30NOV2019]**	1.078	0.831	1.398	0.5632
**[01DEC2020 thru 15DEC2020] vs [01DEC2019 thru 15DEC2019]**	1.105	0.834	1.463	0.4773
**[16DEC2020 thru 31DEC2020] vs [16DEC2019 thru 31DEC2019]**	1.116	0.840	1.481	0.4403
**[01JAN2021 thru 15JAN2021] vs [01JAN2020 thru 15JAN2020]**	1.104	0.843	1.447	0.4628
**[16JAN2021 thru 31JAN2021] vs [16JAN2020 thru 31JAN2020]**	1.079	0.820	1.419	0.5786
**[01FEB2021 thru 15FEB2021] vs [01FEB2020 thru 15FEB2020]**	1.050	0.783	1.407	0.7392
**[16FEB2021 thru 28FEB2021] vs [16FEB2020 thru 29FEB2020]**	1.027	0.763	1.381	0.8585
**[01MAR2020 thru 31MAR2021] vs [01FEB2019 thru 29FEB2020]**	0.988	0.880	1.110	0.8411

Oregon
Bimonthly Data

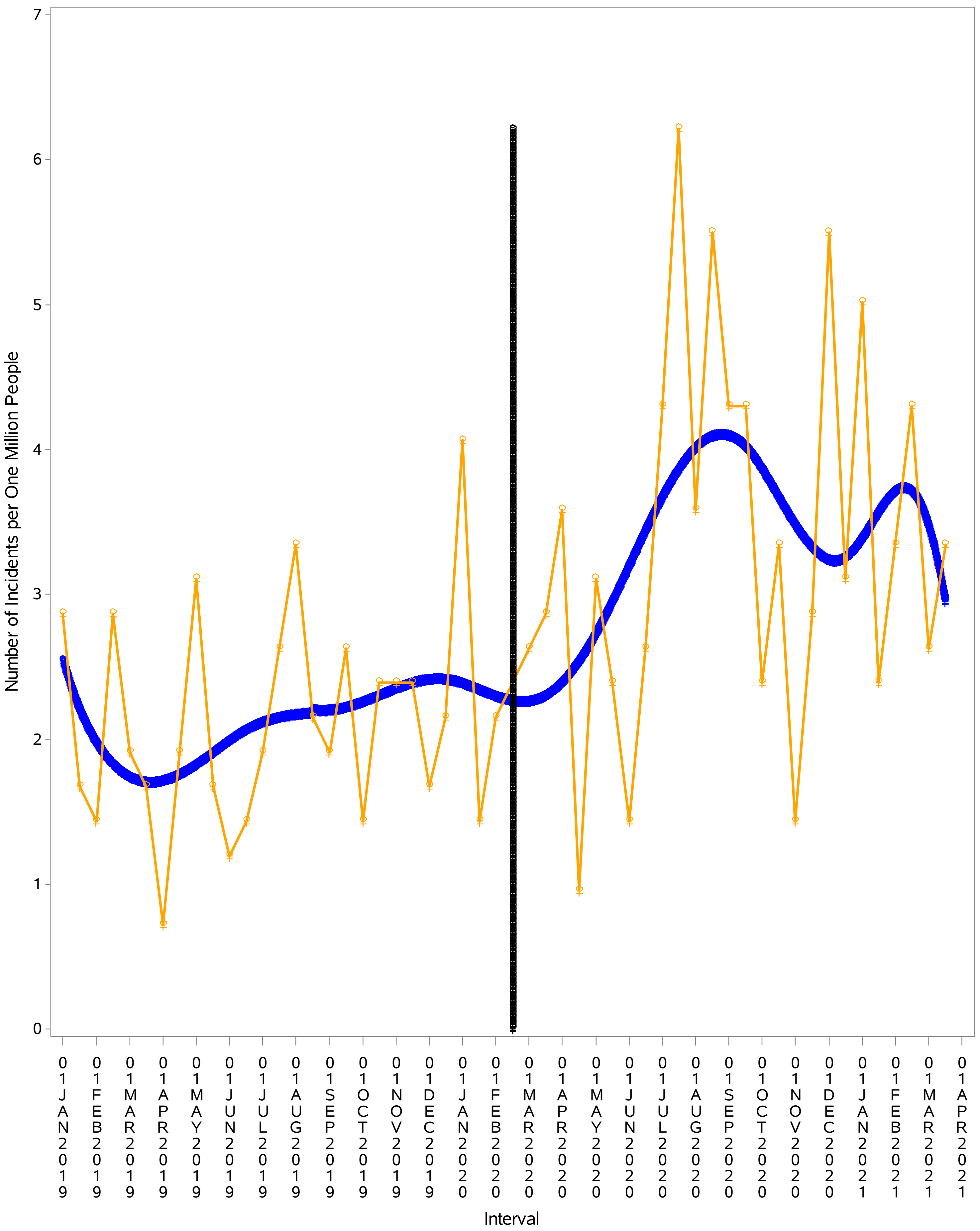

Oregon
Bimonthly Data

Comparison	IntensityRatio	IntensityRatio_LowerCL	IntensityRatio_UpperCL	P_Value
**[01MAR2020 thru 15MAR2020] vs [01MAR2019 thru 15MAR2019]**	1.300	0.755	2.236	0.3356
**[16MAR2020 thru 31MAR2020] vs [16MAR2019 thru 31MAR2019]**	1.349	0.769	2.366	0.2886
**[01APR2020 thru 15APR2020] vs [01APR2019 thru 15APR2019]**	1.397	0.789	2.474	0.2448
**[16APR2020 thru 30APR2020] vs [16APR2019 thru 30APR2019]**	1.445	0.847	2.467	0.1716
**[01MAY2020 thru 15MAY2020] vs [01MAY2019 thru 15MAY2019]**	1.496	0.924	2.421	0.0988
**[16MAY2020 thru 31MAY2020] vs [16MAY2019 thru 31MAY2019]**	1.549	0.980	2.448	0.0605
**[01JUN2020 thru 15JUN2020] vs [01JUN2019 thru 15JUN2019]**	1.606	0.997	2.586	0.0514
**[16JUN2020 thru 30JUN2020] vs [16JUN2019 thru 30JUN2019]**	1.667	1.012	2.747	0.045
**[01JUL2020 thru 15JUL2020] vs [01JUL2019 thru 15JUL2019]**	1.734	1.069	2.812	0.0267
**[16JUL2020 thru 31JUL2020] vs [16JUL2019 thru 31JUL2019]**	1.797	1.156	2.795	0.0105
**[01AUG2020 thru 15AUG2020] vs [01AUG2019 thru 15AUG2019]**	1.847	1.225	2.786	0.0044
**[16AUG2020 thru 31AUG2020] vs [16AUG2019 thru 31AUG2019]**	1.873	1.233	2.847	0.0042
**[01SEP2020 thru 15SEP2020] vs [01SEP2019 thru 15SEP2019]**	1.861	1.189	2.913	0.0077
**[16SEP2020 thru 30SEP2020] vs [16SEP2019 thru 30SEP2019]**	1.809	1.130	2.897	0.0149
**[01OCT2020 thru 15OCT2020] vs [01OCT2019 thru 15OCT2019]**	1.707	1.087	2.681	0.0213
**[16OCT2020 thru 31OCT2020] vs [16OCT2019 thru 31OCT2019]**	1.592	1.046	2.423	0.0309
**[01NOV2020 thru 15NOV2020] vs [01NOV2019 thru 15NOV2019]**	1.479	0.980	2.232	0.062
**[16NOV2020 thru 30NOV2020] vs [16NOV2019 thru 30NOV2019]**	1.390	0.898	2.153	0.1359
**[01DEC2020 thru 15DEC2020] vs [01DEC2019 thru 15DEC2019]**	1.342	0.838	2.149	0.2143
**[16DEC2020 thru 31DEC2020] vs [16DEC2019 thru 31DEC2019]**	1.348	0.837	2.171	0.2131
**[01JAN2021 thru 15JAN2021] vs [01JAN2020 thru 15JAN2020]**	1.418	0.901	2.232	0.1276
**[16JAN2021 thru 31JAN2021] vs [16JAN2020 thru 31JAN2020]**	1.523	0.973	2.383	0.0648
**[01FEB2021 thru 15FEB2021] vs [01FEB2020 thru 15FEB2020]**	1.615	1.013	2.575	0.0441
**[16FEB2021 thru 28FEB2021] vs [16FEB2020 thru 29FEB2020]**	1.637	1.020	2.625	0.0414
**[01MAR2020 thru 31MAR2021] vs [01FEB2019 thru 29FEB2020]**	1.571	1.271	1.942	0.0001

Pennsylvania
Bimonthly Data

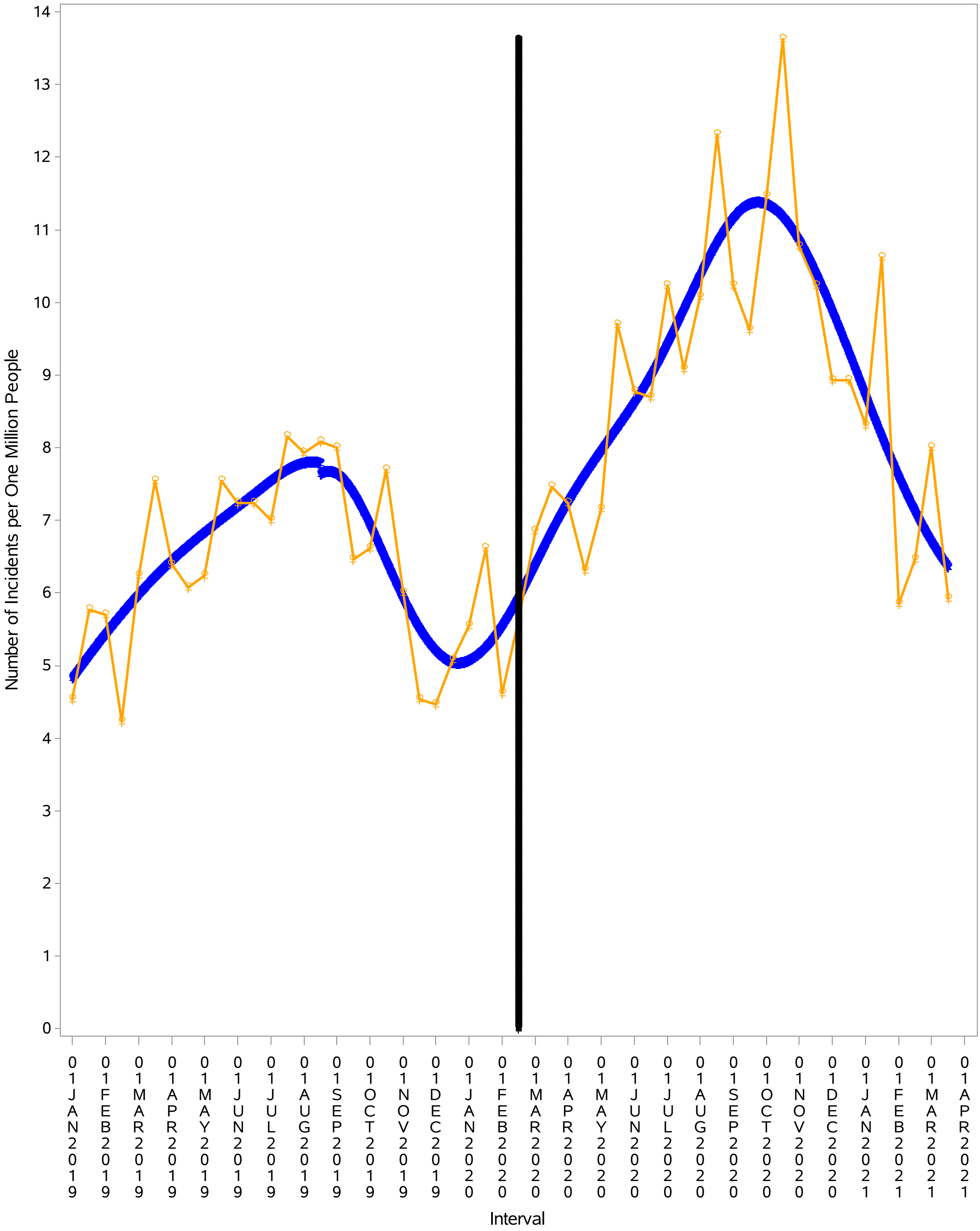

Pennsylvania
Bimonthly Data

Comparison	IntensityRatio	IntensityRatio_LowerCL	IntensityRatio_UpperCL	P_Value
**[01MAR2020 thru 15MAR2020] vs [01MAR2019 thru 15MAR2019]**	1.069	0.887	1.289	0.4758
**[16MAR2020 thru 31MAR2020] vs [16MAR2019 thru 31MAR2019]**	1.101	0.913	1.326	0.3057
**[01APR2020 thru 15APR2020] vs [01APR2019 thru 15APR2019]**	1.127	0.934	1.359	0.2052
**[16APR2020 thru 30APR2020] vs [16APR2019 thru 30APR2019]**	1.148	0.964	1.367	0.1193
**[01MAY2020 thru 15MAY2020] vs [01MAY2019 thru 15MAY2019]**	1.165	0.995	1.366	0.058
**[16MAY2020 thru 31MAY2020] vs [16MAY2019 thru 31MAY2019]**	1.182	1.015	1.377	0.0324
**[01JUN2020 thru 15JUN2020] vs [01JUN2019 thru 15JUN2019]**	1.200	1.022	1.409	0.0267
**[16JUN2020 thru 30JUN2020] vs [16JUN2019 thru 30JUN2019]**	1.222	1.033	1.446	0.0205
**[01JUL2020 thru 15JUL2020] vs [01JUL2019 thru 15JUL2019]**	1.251	1.063	1.472	0.0081
**[16JUL2020 thru 31JUL2020] vs [16JUL2019 thru 31JUL2019]**	1.288	1.110	1.494	0.0013
**[01AUG2020 thru 15AUG2020] vs [01AUG2019 thru 15AUG2019]**	1.333	1.160	1.533	0.0001
**[16AUG2020 thru 31AUG2020] vs [16AUG2019 thru 31AUG2019]**	1.390	1.204	1.604	< 0.0001
**[01SEP2020 thru 15SEP2020] vs [01SEP2019 thru 15SEP2019]**	1.461	1.252	1.706	< 0.0001
**[16SEP2020 thru 30SEP2020] vs [16SEP2019 thru 30SEP2019]**	1.540	1.309	1.812	< 0.0001
**[01OCT2020 thru 15OCT2020] vs [01OCT2019 thru 15OCT2019]**	1.637	1.400	1.913	< 0.0001
**[16OCT2020 thru 31OCT2020] vs [16OCT2019 thru 31OCT2019]**	1.738	1.501	2.014	< 0.0001
**[01NOV2020 thru 15NOV2020] vs [01NOV2019 thru 15NOV2019]**	1.828	1.576	2.121	< 0.0001
**[16NOV2020 thru 30NOV2020] vs [16NOV2019 thru 30NOV2019]**	1.888	1.606	2.220	< 0.0001
**[01DEC2020 thru 15DEC2020] vs [01DEC2019 thru 15DEC2019]**	1.899	1.592	2.265	< 0.0001
**[16DEC2020 thru 31DEC2020] vs [16DEC2019 thru 31DEC2019]**	1.844	1.540	2.208	< 0.0001
**[01JAN2021 thru 15JAN2021] vs [01JAN2020 thru 15JAN2020]**	1.719	1.443	2.048	< 0.0001
**[16JAN2021 thru 31JAN2021] vs [16JAN2020 thru 31JAN2020]**	1.551	1.300	1.852	< 0.0001
**[01FEB2021 thru 15FEB2021] vs [01FEB2020 thru 15FEB2020]**	1.370	1.138	1.650	0.0014
**[16FEB2021 thru 28FEB2021] vs [16FEB2020 thru 29FEB2020]**	1.198	0.994	1.444	0.0569
**[01MAR2020 thru 31MAR2021] vs [01FEB2019 thru 29FEB2020]**	1.380	1.280	1.488	< 0.0001

Rhode Island
Bimonthly Data

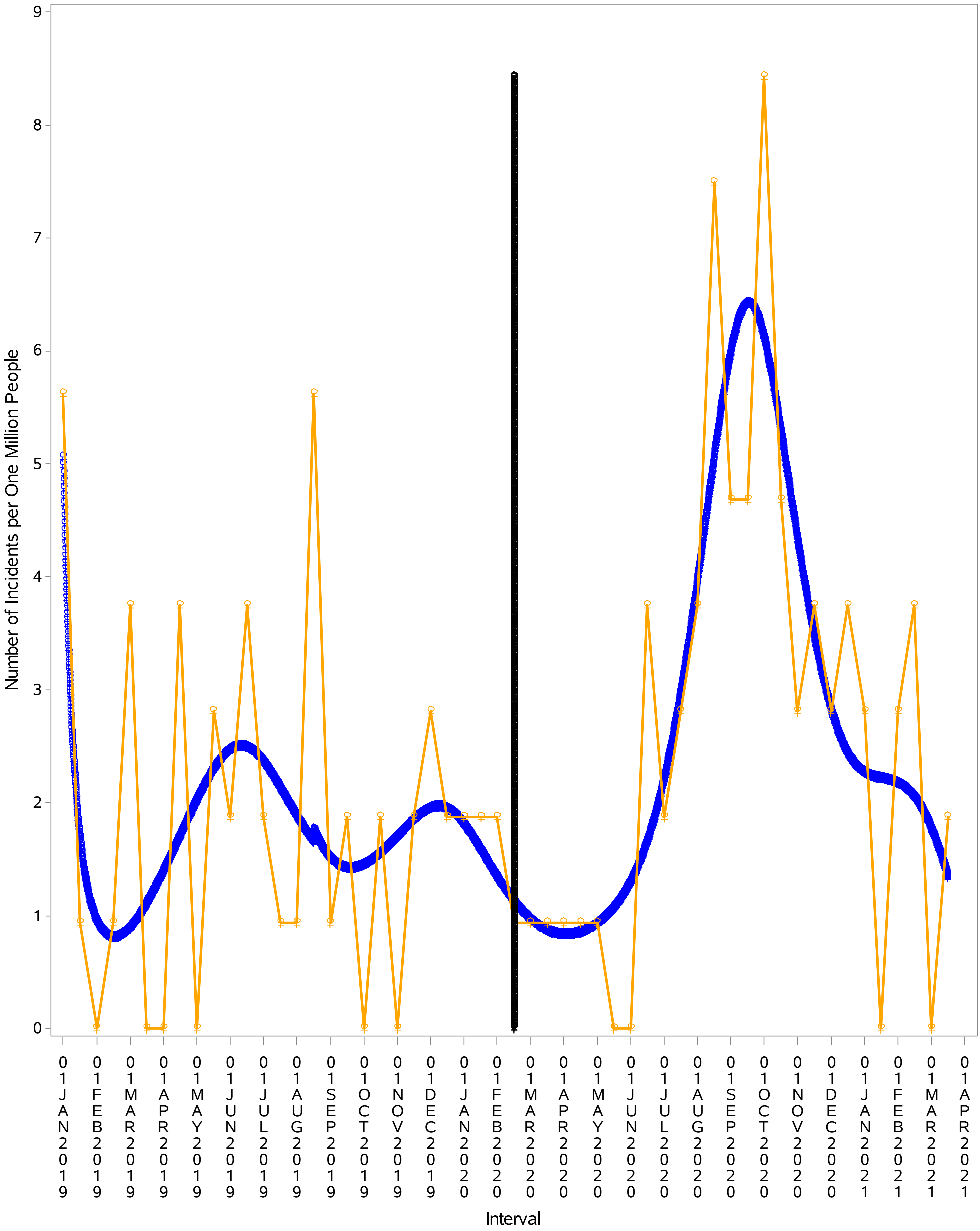

Rhode Island
Bimonthly Data

Comparison	IntensityRatio	IntensityRatio_LowerCL	IntensityRatio_UpperCL	P_Value
**[01MAR2020 thru 15MAR2020] vs [01MAR2019 thru 15MAR2019]**	1.087	0.301	3.921	0.8967
**[16MAR2020 thru 31MAR2020] vs [16MAR2019 thru 31MAR2019]**	0.784	0.220	2.791	0.7006
**[01APR2020 thru 15APR2020] vs [01APR2019 thru 15APR2019]**	0.599	0.172	2.085	0.4117
**[16APR2020 thru 30APR2020] vs [16APR2019 thru 30APR2019]**	0.501	0.159	1.574	0.2297
**[01MAY2020 thru 15MAY2020] vs [01MAY2019 thru 15MAY2019]**	0.460	0.167	1.268	0.1298
**[16MAY2020 thru 31MAY2020] vs [16MAY2019 thru 31MAY2019]**	0.468	0.184	1.191	0.1084
**[01JUN2020 thru 15JUN2020] vs [01JUN2019 thru 15JUN2019]**	0.528	0.206	1.354	0.1786
**[16JUN2020 thru 30JUN2020] vs [16JUN2019 thru 30JUN2019]**	0.667	0.254	1.752	0.4021
**[01JUL2020 thru 15JUL2020] vs [01JUL2019 thru 15JUL2019]**	0.936	0.372	2.359	0.8866
**[16JUL2020 thru 31JUL2020] vs [16JUL2019 thru 31JUL2019]**	1.400	0.608	3.226	0.4199
**[01AUG2020 thru 15AUG2020] vs [01AUG2019 thru 15AUG2019]**	2.114	0.978	4.567	0.0566
**[16AUG2020 thru 31AUG2020] vs [16AUG2019 thru 31AUG2019]**	3.051	1.391	6.693	0.0065
**[01SEP2020 thru 15SEP2020] vs [01SEP2019 thru 15SEP2019]**	3.959	1.706	9.191	0.002
**[16SEP2020 thru 30SEP2020] vs [16SEP2019 thru 30SEP2019]**	4.489	1.843	10.932	0.0015
**[01OCT2020 thru 15OCT2020] vs [01OCT2019 thru 15OCT2019]**	4.164	1.778	9.753	0.0016
**[16OCT2020 thru 31OCT2020] vs [16OCT2019 thru 31OCT2019]**	3.392	1.541	7.468	0.0032
**[01NOV2020 thru 15NOV2020] vs [01NOV2019 thru 15NOV2019]**	2.538	1.168	5.515	0.0198
**[16NOV2020 thru 30NOV2020] vs [16NOV2019 thru 30NOV2019]**	1.870	0.813	4.300	0.1368
**[01DEC2020 thru 15DEC2020] vs [01DEC2019 thru 15DEC2019]**	1.445	0.584	3.574	0.4165
**[16DEC2020 thru 31DEC2020] vs [16DEC2019 thru 31DEC2019]**	1.248	0.492	3.169	0.6338
**[01JAN2021 thru 15JAN2021] vs [01JAN2020 thru 15JAN2020]**	1.255	0.499	3.159	0.6218
**[16JAN2021 thru 31JAN2021] vs [16JAN2020 thru 31JAN2020]**	1.402	0.532	3.694	0.4857
**[01FEB2021 thru 15FEB2021] vs [01FEB2020 thru 15FEB2020]**	1.621	0.564	4.659	0.3608
**[16FEB2021 thru 28FEB2021] vs [16FEB2020 thru 29FEB2020]**	1.811	0.596	5.504	0.2871
**[01MAR2020 thru 31MAR2021] vs [01FEB2019 thru 29FEB2020]**	1.399	0.905	2.162	0.1278

South Carolina
Bimonthly Data

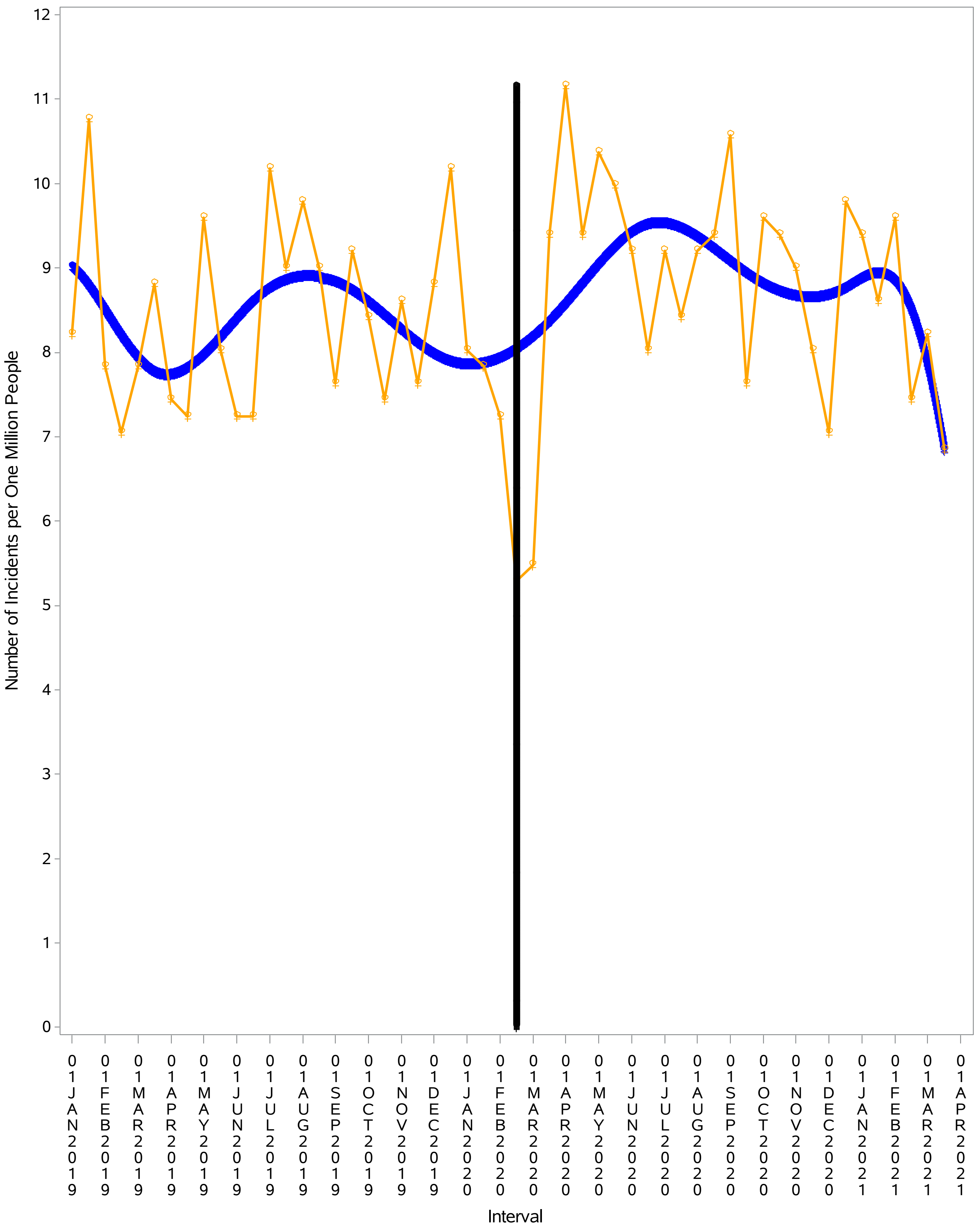

Comparison	IntensityRatio	IntensityRatio_LowerCL	IntensityRatio_UpperCL	P_Value
**[01MAR2020 thru 15MAR2020] vs [01MAR2019 thru 15MAR2019]**	1.032	0.853	1.247	0.7426
**[16MAR2020 thru 31MAR2020] vs [16MAR2019 thru 31MAR2019]**	1.076	0.886	1.309	0.4507
**[01APR2020 thru 15APR2020] vs [01APR2019 thru 15APR2019]**	1.109	0.910	1.351	0.2974
**[16APR2020 thru 30APR2020] vs [16APR2019 thru 30APR2019]**	1.128	0.937	1.357	0.1973
**[01MAY2020 thru 15MAY2020] vs [01MAY2019 thru 15MAY2019]**	1.135	0.958	1.345	0.1405
**[16MAY2020 thru 31MAY2020] vs [16MAY2019 thru 31MAY2019]**	1.132	0.958	1.337	0.1403
**[01JUN2020 thru 15JUN2020] vs [01JUN2019 thru 15JUN2019]**	1.122	0.940	1.338	0.196
**[16JUN2020 thru 30JUN2020] vs [16JUN2019 thru 30JUN2019]**	1.106	0.919	1.332	0.2766
**[01JUL2020 thru 15JUL2020] vs [01JUL2019 thru 15JUL2019]**	1.088	0.910	1.302	0.3455
**[16JUL2020 thru 31JUL2020] vs [16JUL2019 thru 31JUL2019]**	1.070	0.907	1.262	0.415
**[01AUG2020 thru 15AUG2020] vs [01AUG2019 thru 15AUG2019]**	1.052	0.899	1.232	0.519
**[16AUG2020 thru 31AUG2020] vs [16AUG2019 thru 31AUG2019]**	1.037	0.880	1.223	0.6556
**[01SEP2020 thru 15SEP2020] vs [01SEP2019 thru 15SEP2019]**	1.027	0.860	1.228	0.7619
**[16SEP2020 thru 30SEP2020] vs [16SEP2019 thru 30SEP2019]**	1.023	0.849	1.232	0.8094
**[01OCT2020 thru 15OCT2020] vs [01OCT2019 thru 15OCT2019]**	1.025	0.858	1.225	0.7773
**[16OCT2020 thru 31OCT2020] vs [16OCT2019 thru 31OCT2019]**	1.035	0.876	1.222	0.6814
**[01NOV2020 thru 15NOV2020] vs [01NOV2019 thru 15NOV2019]**	1.049	0.889	1.238	0.5633
**[16NOV2020 thru 30NOV2020] vs [16NOV2019 thru 30NOV2019]**	1.067	0.893	1.275	0.4658
**[01DEC2020 thru 15DEC2020] vs [01DEC2019 thru 15DEC2019]**	1.088	0.898	1.317	0.38
**[16DEC2020 thru 31DEC2020] vs [16DEC2019 thru 31DEC2019]**	1.110	0.916	1.345	0.2808
**[01JAN2021 thru 15JAN2021] vs [01JAN2020 thru 15JAN2020]**	1.129	0.941	1.356	0.1859
**[16JAN2021 thru 31JAN2021] vs [16JAN2020 thru 31JAN2020]**	1.136	0.945	1.366	0.1709
**[01FEB2021 thru 15FEB2021] vs [01FEB2020 thru 15FEB2020]**	1.115	0.916	1.358	0.2686
**[16FEB2021 thru 28FEB2021] vs [16FEB2020 thru 29FEB2020]**	1.058	0.866	1.292	0.5736
**[01MAR2020 thru 31MAR2021] vs [01FEB2019 thru 29FEB2020]**	1.063	0.980	1.152	0.1375

South Dakota
Bimonthly Data

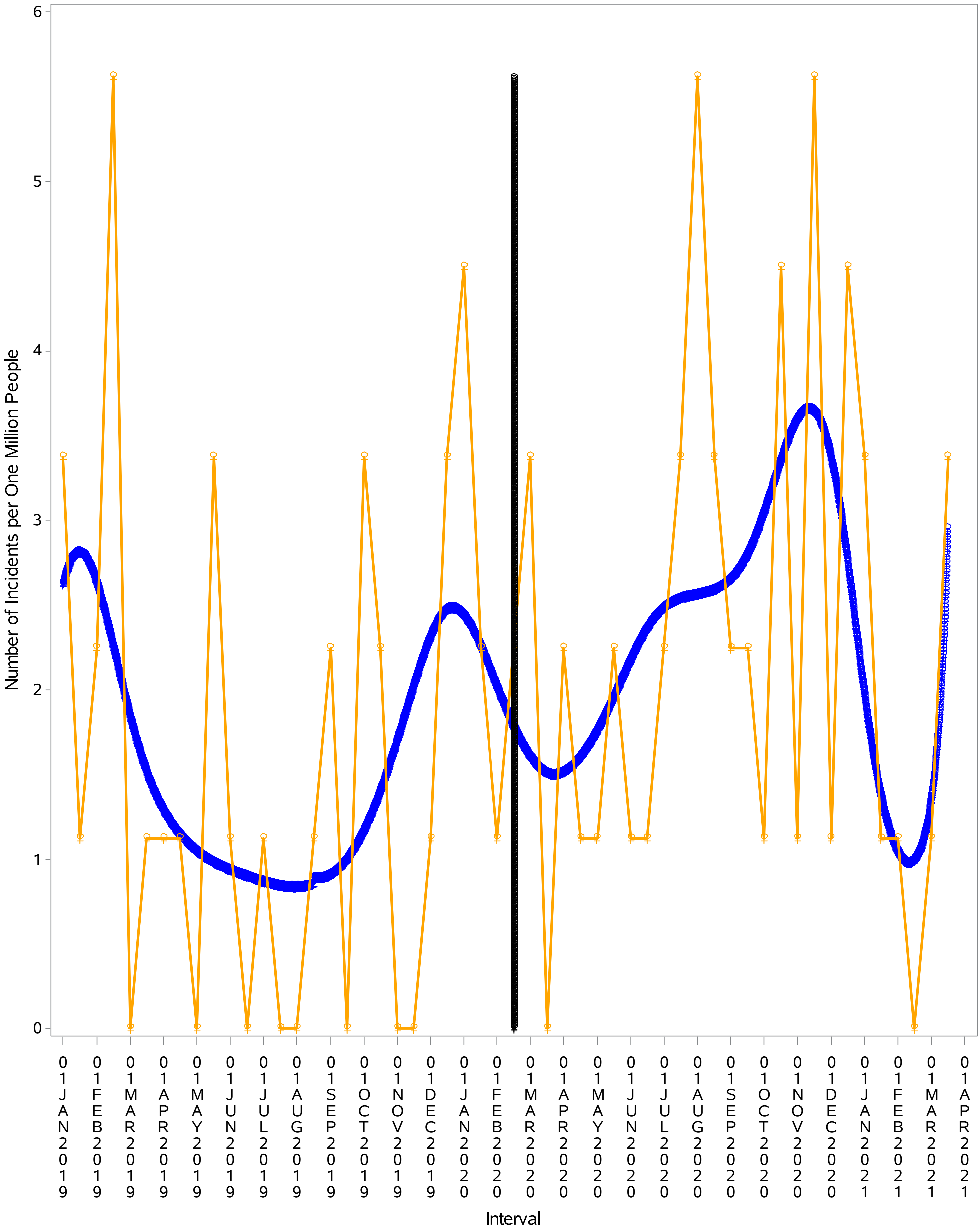

South Dakota
Bimonthly Data

Comparison	IntensityRatio	IntensityRatio_LowerCL	IntensityRatio_UpperCL	P_Value
**[01MAR2020 thru 15MAR2020] vs [01MAR2019 thru 15MAR2019]**	0.870	0.304	2.486	0.7902
**[16MAR2020 thru 31MAR2020] vs [16MAR2019 thru 31MAR2019]**	0.998	0.312	3.198	0.9978
**[01APR2020 thru 15APR2020] vs [01APR2019 thru 15APR2019]**	1.175	0.353	3.914	0.788
**[16APR2020 thru 30APR2020] vs [16APR2019 thru 30APR2019]**	1.402	0.449	4.378	0.5528
**[01MAY2020 thru 15MAY2020] vs [01MAY2019 thru 15MAY2019]**	1.676	0.578	4.861	0.3331
**[16MAY2020 thru 31MAY2020] vs [16MAY2019 thru 31MAY2019]**	1.989	0.679	5.823	0.2036
**[01JUN2020 thru 15JUN2020] vs [01JUN2019 thru 15JUN2019]**	2.316	0.723	7.420	0.1529
**[16JUN2020 thru 30JUN2020] vs [16JUN2019 thru 30JUN2019]**	2.619	0.761	9.018	0.1236
**[01JUL2020 thru 15JUL2020] vs [01JUL2019 thru 15JUL2019]**	2.853	0.854	9.530	0.0867
**[16JUL2020 thru 31JUL2020] vs [16JUL2019 thru 31JUL2019]**	2.999	0.990	9.086	0.0521
**[01AUG2020 thru 15AUG2020] vs [01AUG2019 thru 15AUG2019]**	3.055	1.092	8.541	0.034
**[16AUG2020 thru 31AUG2020] vs [16AUG2019 thru 31AUG2019]**	3.028	1.085	8.450	0.035
**[01SEP2020 thru 15SEP2020] vs [01SEP2019 thru 15SEP2019]**	2.918	0.999	8.528	0.0503
**[16SEP2020 thru 30SEP2020] vs [16SEP2019 thru 30SEP2019]**	2.791	0.921	8.458	0.0686
**[01OCT2020 thru 15OCT2020] vs [01OCT2019 thru 15OCT2019]**	2.626	0.926	7.450	0.0686
**[16OCT2020 thru 31OCT2020] vs [16OCT2019 thru 31OCT2019]**	2.388	0.941	6.059	0.0661
**[01NOV2020 thru 15NOV2020] vs [01NOV2019 thru 15NOV2019]**	2.115	0.906	4.940	0.0819
**[16NOV2020 thru 30NOV2020] vs [16NOV2019 thru 30NOV2019]**	1.798	0.773	4.184	0.168
**[01DEC2020 thru 15DEC2020] vs [01DEC2019 thru 15DEC2019]**	1.455	0.600	3.529	0.3976
**[16DEC2020 thru 31DEC2020] vs [16DEC2019 thru 31DEC2019]**	1.111	0.446	2.766	0.8175
**[01JAN2021 thru 15JAN2021] vs [01JAN2020 thru 15JAN2020]**	0.807	0.313	2.080	0.6501
**[16JAN2021 thru 31JAN2021] vs [16JAN2020 thru 31JAN2020]**	0.606	0.207	1.774	0.3518
**[01FEB2021 thru 15FEB2021] vs [01FEB2020 thru 15FEB2020]**	0.519	0.152	1.766	0.2858
**[16FEB2021 thru 28FEB2021] vs [16FEB2020 thru 29FEB2020]**	0.560	0.160	1.967	0.3573
**[01MAR2020 thru 31MAR2021] vs [01FEB2019 thru 29FEB2020]**	1.528	0.948	2.462	0.0804

Tennessee
Bimonthly Data

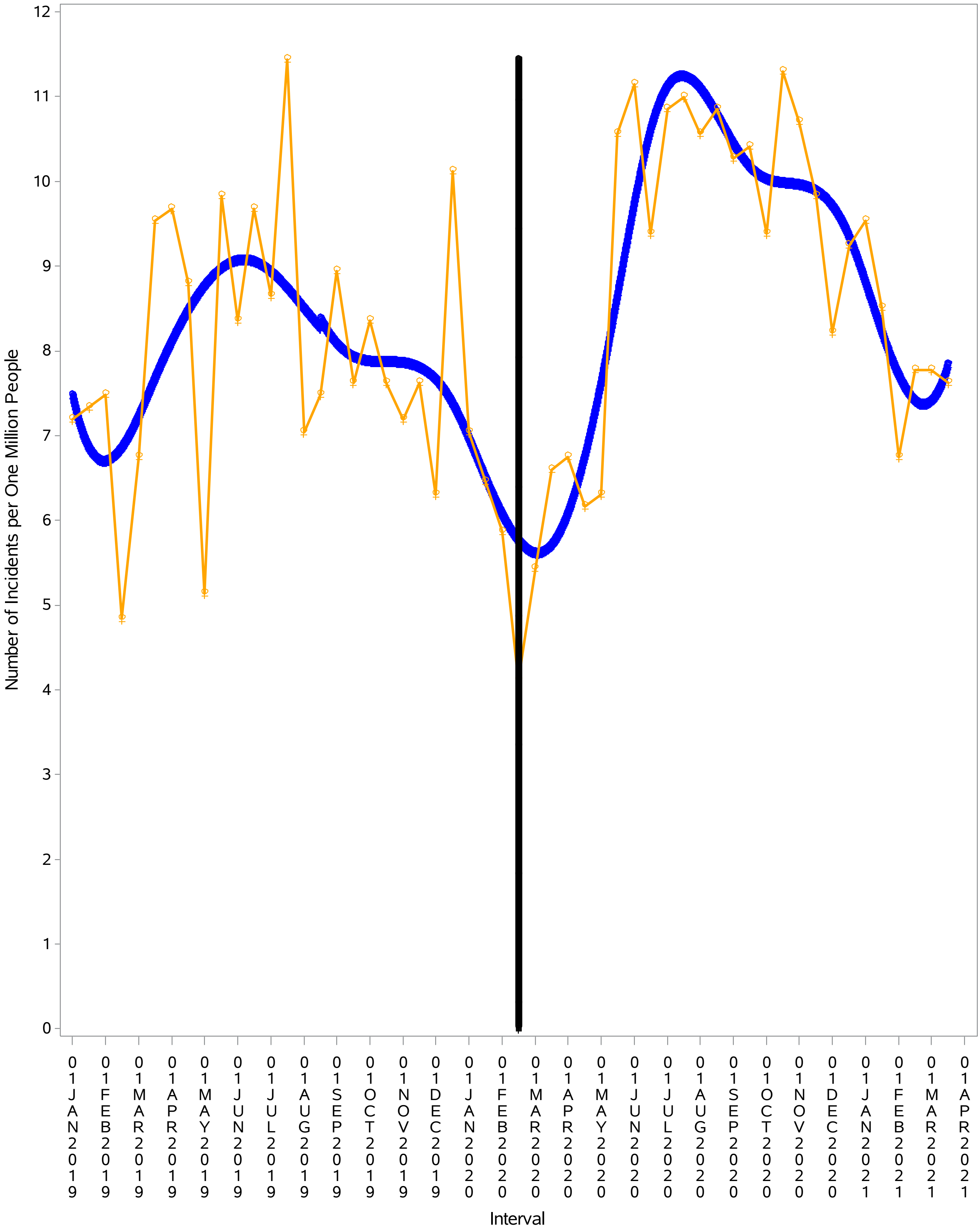

Tennessee
Bimonthly Data

Comparison	IntensityRatio	IntensityRatio_LowerCL	IntensityRatio_UpperCL	P_Value
**[01MAR2020 thru 15MAR2020] vs [01MAR2019 thru 15MAR2019]**	0.777	0.626	0.965	0.0233
**[16MAR2020 thru 31MAR2020] vs [16MAR2019 thru 31MAR2019]**	0.743	0.598	0.923	0.0085
**[01APR2020 thru 15APR2020] vs [01APR2019 thru 15APR2019]**	0.751	0.605	0.932	0.0106
**[16APR2020 thru 30APR2020] vs [16APR2019 thru 30APR2019]**	0.796	0.652	0.972	0.026
**[01MAY2020 thru 15MAY2020] vs [01MAY2019 thru 15MAY2019]**	0.871	0.728	1.042	0.1263
**[16MAY2020 thru 31MAY2020] vs [16MAY2019 thru 31MAY2019]**	0.967	0.814	1.148	0.6943
**[01JUN2020 thru 15JUN2020] vs [01JUN2019 thru 15JUN2019]**	1.073	0.897	1.285	0.4304
**[16JUN2020 thru 30JUN2020] vs [16JUN2019 thru 30JUN2019]**	1.173	0.971	1.415	0.0953
**[01JUL2020 thru 15JUL2020] vs [01JUL2019 thru 15JUL2019]**	1.245	1.037	1.493	0.0197
**[16JUL2020 thru 31JUL2020] vs [16JUL2019 thru 31JUL2019]**	1.287	1.088	1.523	0.0041
**[01AUG2020 thru 15AUG2020] vs [01AUG2019 thru 15AUG2019]**	1.305	1.112	1.532	0.0017
**[16AUG2020 thru 31AUG2020] vs [16AUG2019 thru 31AUG2019]**	1.305	1.103	1.544	0.0027
**[01SEP2020 thru 15SEP2020] vs [01SEP2019 thru 15SEP2019]**	1.292	1.077	1.550	0.007
**[16SEP2020 thru 30SEP2020] vs [16SEP2019 thru 30SEP2019]**	1.281	1.058	1.552	0.0124
**[01OCT2020 thru 15OCT2020] vs [01OCT2019 thru 15OCT2019]**	1.274	1.062	1.529	0.0105
**[16OCT2020 thru 31OCT2020] vs [16OCT2019 thru 31OCT2019]**	1.269	1.070	1.504	0.0074
**[01NOV2020 thru 15NOV2020] vs [01NOV2019 thru 15NOV2019]**	1.267	1.069	1.501	0.0074
**[16NOV2020 thru 30NOV2020] vs [16NOV2019 thru 30NOV2019]**	1.267	1.055	1.522	0.0124
**[01DEC2020 thru 15DEC2020] vs [01DEC2019 thru 15DEC2019]**	1.268	1.040	1.545	0.0199
**[16DEC2020 thru 31DEC2020] vs [16DEC2019 thru 31DEC2019]**	1.267	1.036	1.549	0.0221
**[01JAN2021 thru 15JAN2021] vs [01JAN2020 thru 15JAN2020]**	1.265	1.041	1.537	0.0193
**[16JAN2021 thru 31JAN2021] vs [16JAN2020 thru 31JAN2020]**	1.265	1.034	1.547	0.0232
**[01FEB2021 thru 15FEB2021] vs [01FEB2020 thru 15FEB2020]**	1.271	1.023	1.579	0.0315
**[16FEB2021 thru 28FEB2021] vs [16FEB2020 thru 29FEB2020]**	1.287	1.030	1.609	0.0271
**[01MAR2020 thru 31MAR2021] vs [01FEB2019 thru 29FEB2020]**	1.129	1.037	1.228	0.0061

Texas
Bimonthly Data

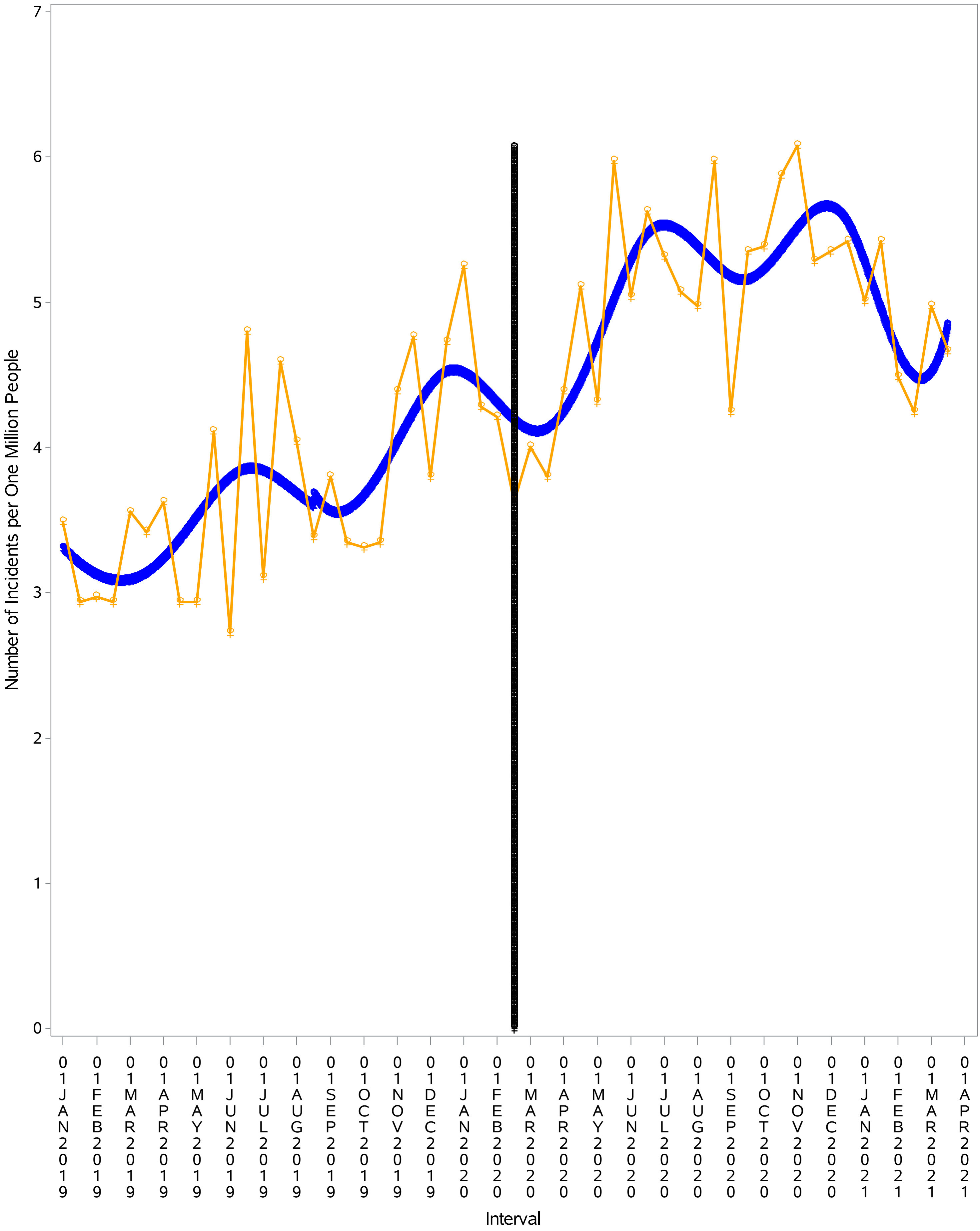

Texas
Bimonthly Data

Comparison	IntensityRatio	IntensityRatio_LowerCL	IntensityRatio_UpperCL	P_Value
**[01MAR2020 thru 15MAR2020] vs [01MAR2019 thru 15MAR2019]**	1.333	1.123	1.582	0.0015
**[16MAR2020 thru 31MAR2020] vs [16MAR2019 thru 31MAR2019]**	1.316	1.107	1.566	0.0026
**[01APR2020 thru 15APR2020] vs [01APR2019 thru 15APR2019]**	1.314	1.102	1.566	0.0031
**[16APR2020 thru 30APR2020] vs [16APR2019 thru 30APR2019]**	1.324	1.123	1.560	0.0013
**[01MAY2020 thru 15MAY2020] vs [01MAY2019 thru 15MAY2019]**	1.343	1.157	1.558	0.0003
**[16MAY2020 thru 31MAY2020] vs [16MAY2019 thru 31MAY2019]**	1.368	1.185	1.578	0.0001
**[01JUN2020 thru 15JUN2020] vs [01JUN2019 thru 15JUN2019]**	1.395	1.200	1.621	0.0001
**[16JUN2020 thru 30JUN2020] vs [16JUN2019 thru 30JUN2019]**	1.420	1.212	1.663	0.0001
**[01JUL2020 thru 15JUL2020] vs [01JUL2019 thru 15JUL2019]**	1.440	1.234	1.680	< 0.0001
**[16JUL2020 thru 31JUL2020] vs [16JUL2019 thru 31JUL2019]**	1.454	1.261	1.676	< 0.0001
**[01AUG2020 thru 15AUG2020] vs [01AUG2019 thru 15AUG2019]**	1.461	1.276	1.673	< 0.0001
**[16AUG2020 thru 31AUG2020] vs [16AUG2019 thru 31AUG2019]**	1.462	1.271	1.683	< 0.0001
**[01SEP2020 thru 15SEP2020] vs [01SEP2019 thru 15SEP2019]**	1.452	1.249	1.690	< 0.0001
**[16SEP2020 thru 30SEP2020] vs [16SEP2019 thru 30SEP2019]**	1.444	1.233	1.692	< 0.0001
**[01OCT2020 thru 15OCT2020] vs [01OCT2019 thru 15OCT2019]**	1.427	1.228	1.660	< 0.0001
**[16OCT2020 thru 31OCT2020] vs [16OCT2019 thru 31OCT2019]**	1.399	1.218	1.607	< 0.0001
**[01NOV2020 thru 15NOV2020] vs [01NOV2019 thru 15NOV2019]**	1.366	1.195	1.562	< 0.0001
**[16NOV2020 thru 30NOV2020] vs [16NOV2019 thru 30NOV2019]**	1.326	1.153	1.525	0.0002
**[01DEC2020 thru 15DEC2020] vs [01DEC2019 thru 15DEC2019]**	1.279	1.102	1.485	0.0018
**[16DEC2020 thru 31DEC2020] vs [16DEC2019 thru 31DEC2019]**	1.226	1.054	1.425	0.0093
**[01JAN2021 thru 15JAN2021] vs [01JAN2020 thru 15JAN2020]**	1.168	1.010	1.350	0.0363
**[16JAN2021 thru 31JAN2021] vs [16JAN2020 thru 31JAN2020]**	1.116	0.963	1.295	0.1416
**[01FEB2021 thru 15FEB2021] vs [01FEB2020 thru 15FEB2020]**	1.081	0.922	1.268	0.3306
**[16FEB2021 thru 28FEB2021] vs [16FEB2020 thru 29FEB2020]**	1.071	0.910	1.260	0.3994
**[01MAR2020 thru 31MAR2021] vs [01FEB2019 thru 29FEB2020]**	1.339	1.250	1.433	< 0.0001

Utah

Bimonthly Data

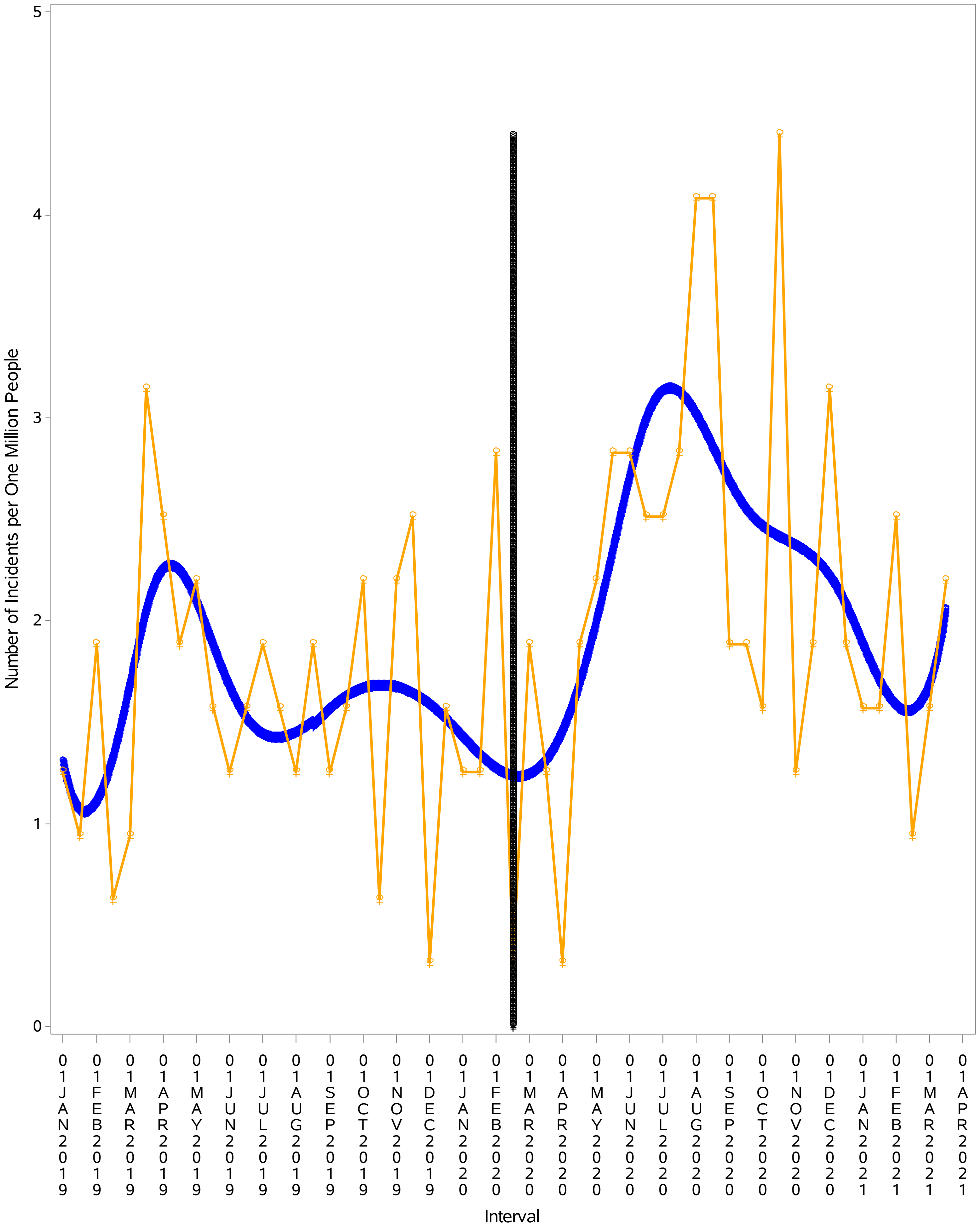

Utah
Bimonthly Data

Comparison	IntensityRatio	IntensityRatio_LowerCL	IntensityRatio_UpperCL	P_Value
**[01MAR2020 thru 15MAR2020] vs [01MAR2019 thru 15MAR2019]**	0.741	0.406	1.350	0.3184
**[16MAR2020 thru 31MAR2020] vs [16MAR2019 thru 31MAR2019]**	0.640	0.358	1.143	0.1278
**[01APR2020 thru 15APR2020] vs [01APR2019 thru 15APR2019]**	0.648	0.366	1.149	0.1337
**[16APR2020 thru 30APR2020] vs [16APR2019 thru 30APR2019]**	0.753	0.444	1.277	0.2839
**[01MAY2020 thru 15MAY2020] vs [01MAY2019 thru 15MAY2019]**	0.952	0.590	1.537	0.8377
**[16MAY2020 thru 31MAY2020] vs [16MAY2019 thru 31MAY2019]**	1.250	0.783	1.995	0.3415
**[01JUN2020 thru 15JUN2020] vs [01JUN2019 thru 15JUN2019]**	1.619	0.980	2.674	0.0594
**[16JUN2020 thru 30JUN2020] vs [16JUN2019 thru 30JUN2019]**	1.970	1.155	3.360	0.014
**[01JUL2020 thru 15JUL2020] vs [01JUL2019 thru 15JUL2019]**	2.170	1.289	3.653	0.0045
**[16JUL2020 thru 31JUL2020] vs [16JUL2019 thru 31JUL2019]**	2.191	1.355	3.541	0.002
**[01AUG2020 thru 15AUG2020] vs [01AUG2019 thru 15AUG2019]**	2.078	1.318	3.275	0.0023
**[16AUG2020 thru 31AUG2020] vs [16AUG2019 thru 31AUG2019]**	1.899	1.186	3.041	0.0088
**[01SEP2020 thru 15SEP2020] vs [01SEP2019 thru 15SEP2019]**	1.717	1.035	2.848	0.0369
**[16SEP2020 thru 30SEP2020] vs [16SEP2019 thru 30SEP2019]**	1.567	0.922	2.662	0.0949
**[01OCT2020 thru 15OCT2020] vs [01OCT2019 thru 15OCT2019]**	1.481	0.892	2.456	0.1252
**[16OCT2020 thru 31OCT2020] vs [16OCT2019 thru 31OCT2019]**	1.434	0.893	2.301	0.1318
**[01NOV2020 thru 15NOV2020] vs [01NOV2019 thru 15NOV2019]**	1.416	0.884	2.265	0.1434
**[16NOV2020 thru 30NOV2020] vs [16NOV2019 thru 30NOV2019]**	1.408	0.848	2.339	0.1803
**[01DEC2020 thru 15DEC2020] vs [01DEC2019 thru 15DEC2019]**	1.398	0.806	2.424	0.2259
**[16DEC2020 thru 31DEC2020] vs [16DEC2019 thru 31DEC2019]**	1.371	0.781	2.408	0.264
**[01JAN2021 thru 15JAN2021] vs [01JAN2020 thru 15JAN2020]**	1.322	0.762	2.295	0.3125
**[16JAN2021 thru 31JAN2021] vs [16JAN2020 thru 31JAN2020]**	1.271	0.719	2.250	0.4006
**[01FEB2021 thru 15FEB2021] vs [01FEB2020 thru 15FEB2020]**	1.244	0.674	2.296	0.4771
**[16FEB2021 thru 28FEB2021] vs [16FEB2020 thru 29FEB2020]**	1.262	0.678	2.348	0.4545
**[01MAR2020 thru 31MAR2021] vs [01FEB2019 thru 29FEB2020]**	1.357	1.071	1.719	0.0127

Vermont
Bimonthly Data

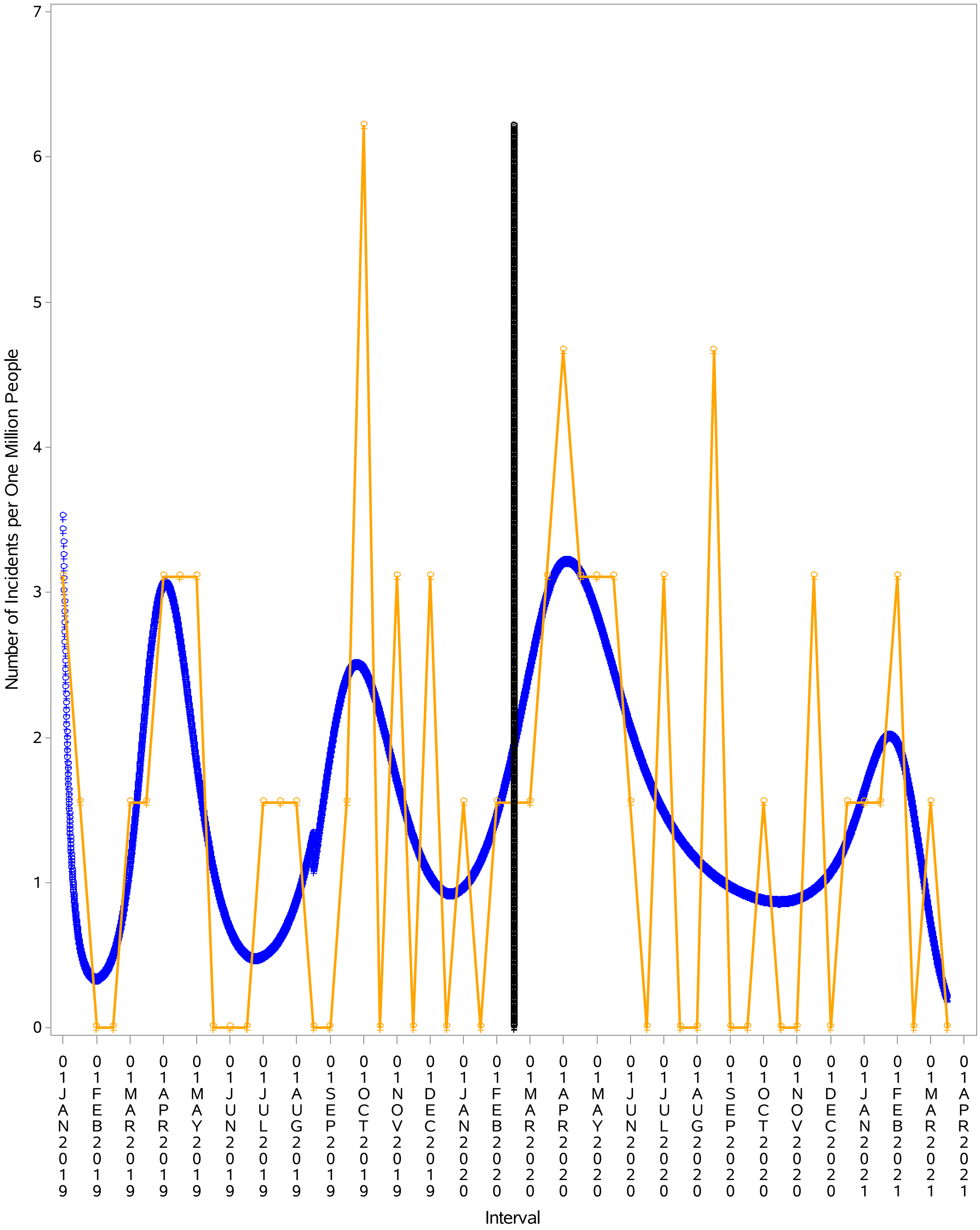

Vermont
Bimonthly Data

Comparison	IntensityRatio	IntensityRatio_LowerCL	IntensityRatio_UpperCL	P_Value
**[01MAR2020 thru 15MAR2020] vs [01MAR2019 thru 15MAR2019]**	2.226	0.554	8.939	0.2521
**[16MAR2020 thru 31MAR2020] vs [16MAR2019 thru 31MAR2019]**	1.289	0.423	3.932	0.6485
**[01APR2020 thru 15APR2020] vs [01APR2019 thru 15APR2019]**	1.052	0.365	3.029	0.9237
**[16APR2020 thru 30APR2020] vs [16APR2019 thru 30APR2019]**	1.173	0.421	3.264	0.755
**[01MAY2020 thru 15MAY2020] vs [01MAY2019 thru 15MAY2019]**	1.579	0.554	4.500	0.3836
**[16MAY2020 thru 31MAY2020] vs [16MAY2019 thru 31MAY2019]**	2.270	0.688	7.491	0.1732
**[01JUN2020 thru 15JUN2020] vs [01JUN2019 thru 15JUN2019]**	3.079	0.757	12.516	0.1131
**[16JUN2020 thru 30JUN2020] vs [16JUN2019 thru 30JUN2019]**	3.483	0.745	16.279	0.1099
**[01JUL2020 thru 15JUL2020] vs [01JUL2019 thru 15JUL2019]**	3.011	0.655	13.847	0.1523
**[16JUL2020 thru 31JUL2020] vs [16JUL2019 thru 31JUL2019]**	2.105	0.512	8.651	0.2942
**[01AUG2020 thru 15AUG2020] vs [01AUG2019 thru 15AUG2019]**	1.304	0.350	4.857	0.6857
**[16AUG2020 thru 31AUG2020] vs [16AUG2019 thru 31AUG2019]**	0.786	0.213	2.896	0.711
**[01SEP2020 thru 15SEP2020] vs [01SEP2019 thru 15SEP2019]**	0.519	0.134	2.009	0.3334
**[16SEP2020 thru 30SEP2020] vs [16SEP2019 thru 30SEP2019]**	0.379	0.095	1.509	0.164
**[01OCT2020 thru 15OCT2020] vs [01OCT2019 thru 15OCT2019]**	0.356	0.096	1.319	0.1191
**[16OCT2020 thru 31OCT2020] vs [16OCT2019 thru 31OCT2019]**	0.405	0.116	1.409	0.1508
**[01NOV2020 thru 15NOV2020] vs [01NOV2019 thru 15NOV2019]**	0.524	0.147	1.876	0.3127
**[16NOV2020 thru 30NOV2020] vs [16NOV2019 thru 30NOV2019]**	0.730	0.182	2.932	0.6502
**[01DEC2020 thru 15DEC2020] vs [01DEC2019 thru 15DEC2019]**	1.030	0.233	4.550	0.9682
**[16DEC2020 thru 31DEC2020] vs [16DEC2019 thru 31DEC2019]**	1.388	0.322	5.979	0.6526
**[01JAN2021 thru 15JAN2021] vs [01JAN2020 thru 15JAN2020]**	1.681	0.440	6.428	0.4388
**[16JAN2021 thru 31JAN2021] vs [16JAN2020 thru 31JAN2020]**	1.701	0.460	6.292	0.4174
**[01FEB2021 thru 15FEB2021] vs [01FEB2020 thru 15FEB2020]**	1.333	0.351	5.054	0.6659
**[16FEB2021 thru 28FEB2021] vs [16FEB2020 thru 29FEB2020]**	0.750	0.191	2.945	0.6736
**[01MAR2020 thru 31MAR2021] vs [01FEB2019 thru 29FEB2020]**	1.198	0.614	2.338	0.5884

Virginia
Bimonthly Data

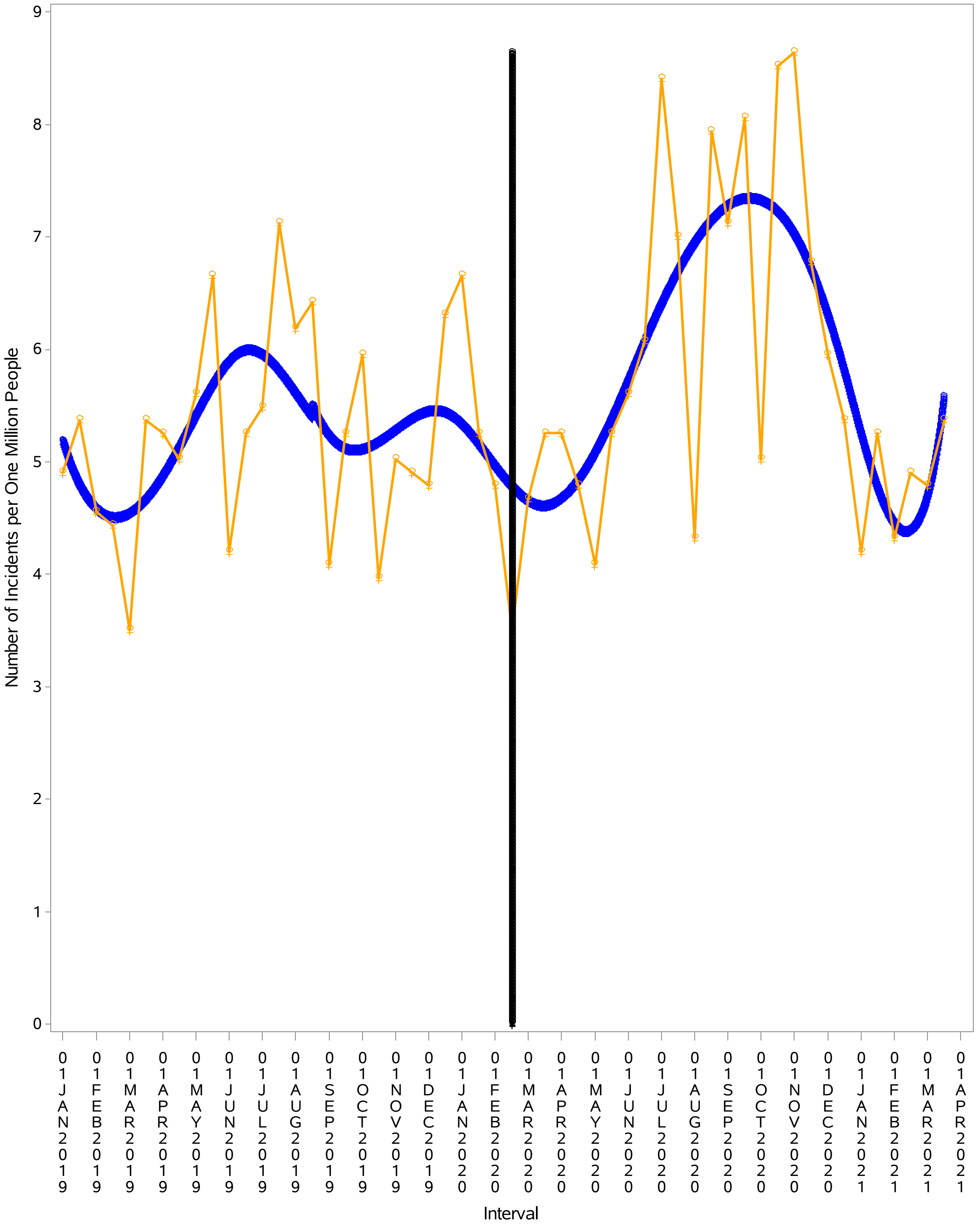

Virginia
Bimonthly Data

Comparison	IntensityRatio	IntensityRatio_LowerCL	IntensityRatio_UpperCL	P_Value
**[01MAR2020 thru 15MAR2020] vs [01MAR2019 thru 15MAR2019]**	1.025	0.805	1.304	0.8393
**[16MAR2020 thru 31MAR2020] vs [16MAR2019 thru 31MAR2019]**	0.988	0.774	1.261	0.9199
**[01APR2020 thru 15APR2020] vs [01APR2019 thru 15APR2019]**	0.960	0.751	1.226	0.7374
**[16APR2020 thru 30APR2020] vs [16APR2019 thru 30APR2019]**	0.943	0.750	1.185	0.6076
**[01MAY2020 thru 15MAY2020] vs [01MAY2019 thru 15MAY2019]**	0.938	0.762	1.155	0.5404
**[16MAY2020 thru 31MAY2020] vs [16MAY2019 thru 31MAY2019]**	0.947	0.775	1.158	0.5884
**[01JUN2020 thru 15JUN2020] vs [01JUN2019 thru 15JUN2019]**	0.971	0.786	1.199	0.7813
**[16JUN2020 thru 30JUN2020] vs [16JUN2019 thru 30JUN2019]**	1.013	0.812	1.264	0.9049
**[01JUL2020 thru 15JUL2020] vs [01JUL2019 thru 15JUL2019]**	1.075	0.869	1.332	0.4958
**[16JUL2020 thru 31JUL2020] vs [16JUL2019 thru 31JUL2019]**	1.154	0.948	1.404	0.1492
**[01AUG2020 thru 15AUG2020] vs [01AUG2019 thru 15AUG2019]**	1.240	1.030	1.493	0.0244
**[16AUG2020 thru 31AUG2020] vs [16AUG2019 thru 31AUG2019]**	1.325	1.092	1.607	0.0054
**[01SEP2020 thru 15SEP2020] vs [01SEP2019 thru 15SEP2019]**	1.391	1.130	1.713	0.0026
**[16SEP2020 thru 30SEP2020] vs [16SEP2019 thru 30SEP2019]**	1.435	1.154	1.784	0.0018
**[01OCT2020 thru 15OCT2020] vs [01OCT2019 thru 15OCT2019]**	1.434	1.165	1.765	0.0011
**[16OCT2020 thru 31OCT2020] vs [16OCT2019 thru 31OCT2019]**	1.395	1.150	1.693	0.0012
**[01NOV2020 thru 15NOV2020] vs [01NOV2019 thru 15NOV2019]**	1.330	1.097	1.611	0.0046
**[16NOV2020 thru 30NOV2020] vs [16NOV2019 thru 30NOV2019]**	1.247	1.014	1.532	0.0367
**[01DEC2020 thru 15DEC2020] vs [01DEC2019 thru 15DEC2019]**	1.156	0.926	1.445	0.1949
**[16DEC2020 thru 31DEC2020] vs [16DEC2019 thru 31DEC2019]**	1.066	0.850	1.337	0.57
**[01JAN2021 thru 15JAN2021] vs [01JAN2020 thru 15JAN2020]**	0.984	0.789	1.228	0.886
**[16JAN2021 thru 31JAN2021] vs [16JAN2020 thru 31JAN2020]**	0.924	0.733	1.165	0.4952
**[01FEB2021 thru 15FEB2021] vs [01FEB2020 thru 15FEB2020]**	0.898	0.698	1.156	0.395
**[16FEB2021 thru 28FEB2021] vs [16FEB2020 thru 29FEB2020]**	0.919	0.711	1.189	0.5132
**[01MAR2020 thru 31MAR2021] vs [01FEB2019 thru 29FEB2020]**	1.102	0.999	1.214	0.0512

Washington
Bimonthly Data

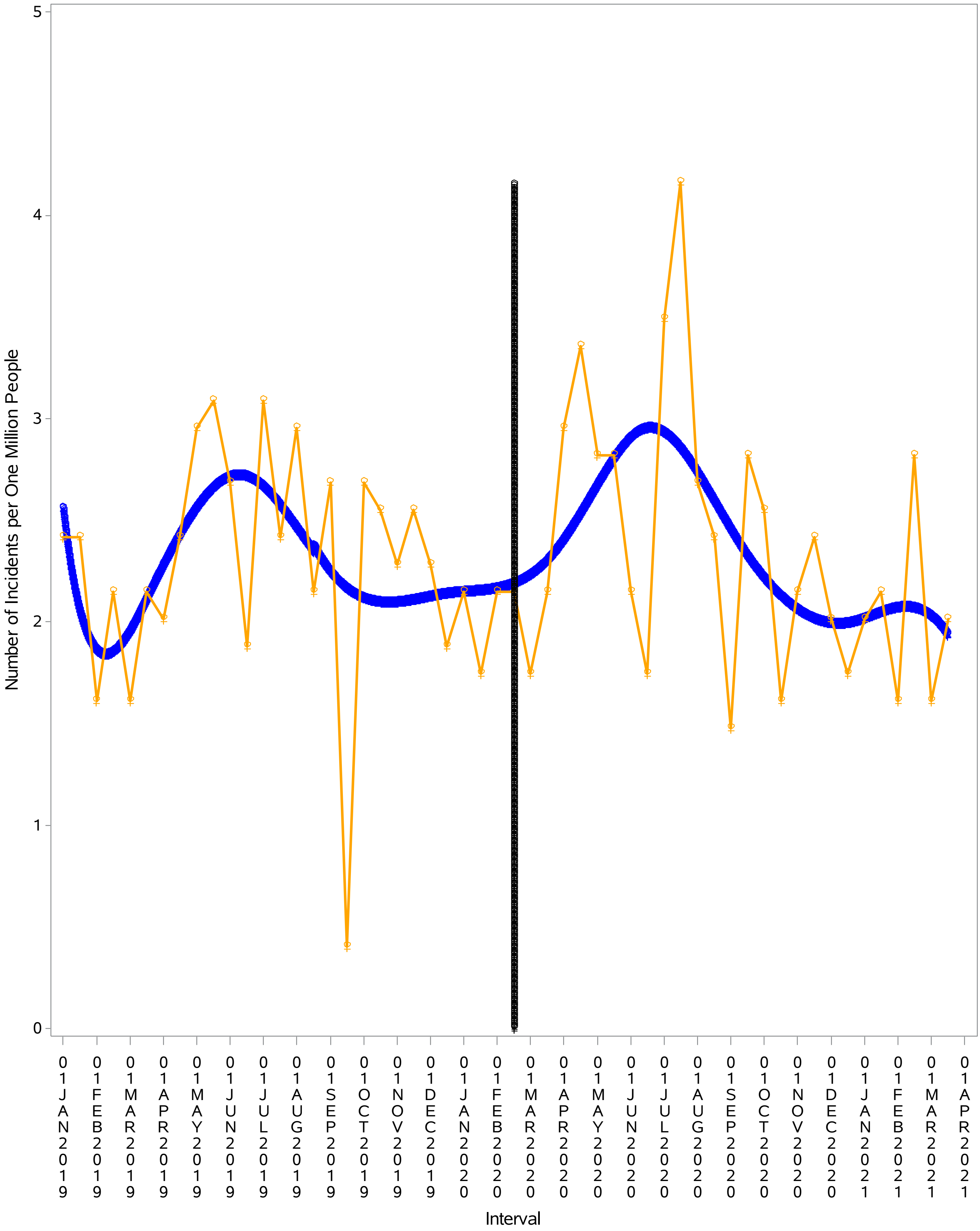

Washington
Bimonthly Data

Comparison	IntensityRatio	IntensityRatio_LowerCL	IntensityRatio_UpperCL	P_Value
**[01MAR2020 thru 15MAR2020] vs [01MAR2019 thru 15MAR2019]**	1.142	0.826	1.580	0.4123
**[16MAR2020 thru 31MAR2020] vs [16MAR2019 thru 31MAR2019]**	1.090	0.789	1.506	0.5946
**[01APR2020 thru 15APR2020] vs [01APR2019 thru 15APR2019]**	1.056	0.765	1.459	0.734
**[16APR2020 thru 30APR2020] vs [16APR2019 thru 30APR2019]**	1.043	0.772	1.408	0.7803
**[01MAY2020 thru 15MAY2020] vs [01MAY2019 thru 15MAY2019]**	1.044	0.795	1.371	0.754
**[16MAY2020 thru 31MAY2020] vs [16MAY2019 thru 31MAY2019]**	1.054	0.809	1.374	0.6895
**[01JUN2020 thru 15JUN2020] vs [01JUN2019 thru 15JUN2019]**	1.070	0.808	1.417	0.6278
**[16JUN2020 thru 30JUN2020] vs [16JUN2019 thru 30JUN2019]**	1.087	0.809	1.460	0.5715
**[01JUL2020 thru 15JUL2020] vs [01JUL2019 thru 15JUL2019]**	1.100	0.826	1.466	0.5051
**[16JUL2020 thru 31JUL2020] vs [16JUL2019 thru 31JUL2019]**	1.108	0.848	1.448	0.4413
**[01AUG2020 thru 15AUG2020] vs [01AUG2019 thru 15AUG2019]**	1.111	0.856	1.442	0.4216
**[16AUG2020 thru 31AUG2020] vs [16AUG2019 thru 31AUG2019]**	1.106	0.837	1.461	0.4702
**[01SEP2020 thru 15SEP2020] vs [01SEP2019 thru 15SEP2019]**	1.092	0.805	1.480	0.5635
**[16SEP2020 thru 30SEP2020] vs [16SEP2019 thru 30SEP2019]**	1.073	0.779	1.477	0.6585
**[01OCT2020 thru 15OCT2020] vs [01OCT2019 thru 15OCT2019]**	1.045	0.769	1.419	0.7736
**[16OCT2020 thru 31OCT2020] vs [16OCT2019 thru 31OCT2019]**	1.013	0.759	1.353	0.9263
**[01NOV2020 thru 15NOV2020] vs [01NOV2019 thru 15NOV2019]**	0.983	0.735	1.314	0.9033
**[16NOV2020 thru 30NOV2020] vs [16NOV2019 thru 30NOV2019]**	0.956	0.699	1.308	0.7755
**[01DEC2020 thru 15DEC2020] vs [01DEC2019 thru 15DEC2019]**	0.939	0.671	1.313	0.705
**[16DEC2020 thru 31DEC2020] vs [16DEC2019 thru 31DEC2019]**	0.932	0.666	1.305	0.6759
**[01JAN2021 thru 15JAN2021] vs [01JAN2020 thru 15JAN2020]**	0.939	0.682	1.292	0.6916
**[16JAN2021 thru 31JAN2021] vs [16JAN2020 thru 31JAN2020]**	0.950	0.688	1.312	0.7515
**[01FEB2021 thru 15FEB2021] vs [01FEB2020 thru 15FEB2020]**	0.956	0.677	1.351	0.7947
**[16FEB2021 thru 28FEB2021] vs [16FEB2020 thru 29FEB2020]**	0.946	0.667	1.340	0.7485
**[01MAR2020 thru 31MAR2021] vs [01FEB2019 thru 29FEB2020]**	1.037	0.904	1.189	0.5945

West Virginia
Bimonthly Data

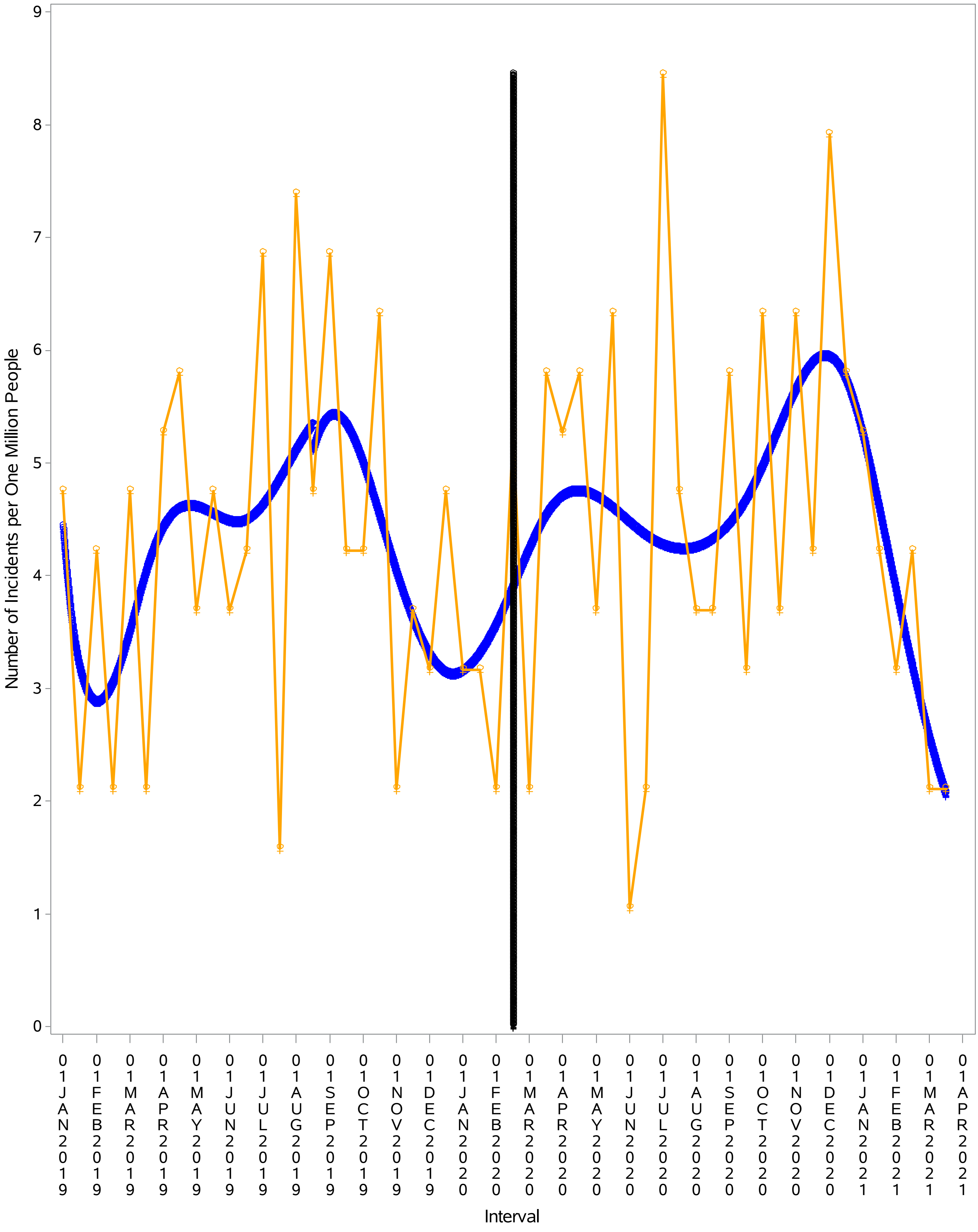

West Virginia
Bimonthly Data

Comparison	IntensityRatio	IntensityRatio_LowerCL	IntensityRatio_UpperCL	P_Value
**[01MAR2020 thru 15MAR2020] vs [01MAR2019 thru 15MAR2019]**	1.212	0.728	2.020	0.4504
**[16MAR2020 thru 31MAR2020] vs [16MAR2019 thru 31MAR2019]**	1.123	0.683	1.846	0.6409
**[01APR2020 thru 15APR2020] vs [01APR2019 thru 15APR2019]**	1.064	0.649	1.746	0.8014
**[16APR2020 thru 30APR2020] vs [16APR2019 thru 30APR2019]**	1.034	0.650	1.644	0.8858
**[01MAY2020 thru 15MAY2020] vs [01MAY2019 thru 15MAY2019]**	1.020	0.665	1.564	0.9277
**[16MAY2020 thru 31MAY2020] vs [16MAY2019 thru 31MAY2019]**	1.011	0.661	1.544	0.9598
**[01JUN2020 thru 15JUN2020] vs [01JUN2019 thru 15JUN2019]**	0.997	0.634	1.570	0.9904
**[16JUN2020 thru 30JUN2020] vs [16JUN2019 thru 30JUN2019]**	0.970	0.601	1.565	0.8979
**[01JUL2020 thru 15JUL2020] vs [01JUL2019 thru 15JUL2019]**	0.924	0.581	1.470	0.7342
**[16JUL2020 thru 31JUL2020] vs [16JUL2019 thru 31JUL2019]**	0.874	0.570	1.338	0.5258
**[01AUG2020 thru 15AUG2020] vs [01AUG2019 thru 15AUG2019]**	0.831	0.556	1.244	0.3602
**[16AUG2020 thru 31AUG2020] vs [16AUG2019 thru 31AUG2019]**	0.809	0.534	1.227	0.3107
**[01SEP2020 thru 15SEP2020] vs [01SEP2019 thru 15SEP2019]**	0.823	0.527	1.286	0.3844
**[16SEP2020 thru 30SEP2020] vs [16SEP2019 thru 30SEP2019]**	0.875	0.550	1.392	0.5642
**[01OCT2020 thru 15OCT2020] vs [01OCT2019 thru 15OCT2019]**	0.997	0.642	1.547	0.9877
**[16OCT2020 thru 31OCT2020] vs [16OCT2019 thru 31OCT2019]**	1.176	0.779	1.773	0.4315
**[01NOV2020 thru 15NOV2020] vs [01NOV2019 thru 15NOV2019]**	1.401	0.929	2.113	0.1055
**[16NOV2020 thru 30NOV2020] vs [16NOV2019 thru 30NOV2019]**	1.631	1.044	2.550	0.0325
**[01DEC2020 thru 15DEC2020] vs [01DEC2019 thru 15DEC2019]**	1.801	1.109	2.924	0.0187
**[16DEC2020 thru 31DEC2020] vs [16DEC2019 thru 31DEC2019]**	1.826	1.115	2.990	0.018
**[01JAN2021 thru 15JAN2021] vs [01JAN2020 thru 15JAN2020]**	1.665	1.028	2.696	0.0388
**[16JAN2021 thru 31JAN2021] vs [16JAN2020 thru 31JAN2020]**	1.387	0.839	2.293	0.1962
**[01FEB2021 thru 15FEB2021] vs [01FEB2020 thru 15FEB2020]**	1.083	0.631	1.861	0.7663
**[16FEB2021 thru 28FEB2021] vs [16FEB2020 thru 29FEB2020]**	0.814	0.469	1.413	0.456
**[01MAR2020 thru 31MAR2021] vs [01FEB2019 thru 29FEB2020]**	1.082	0.874	1.340	0.462

Wisconsin
Bimonthly Data

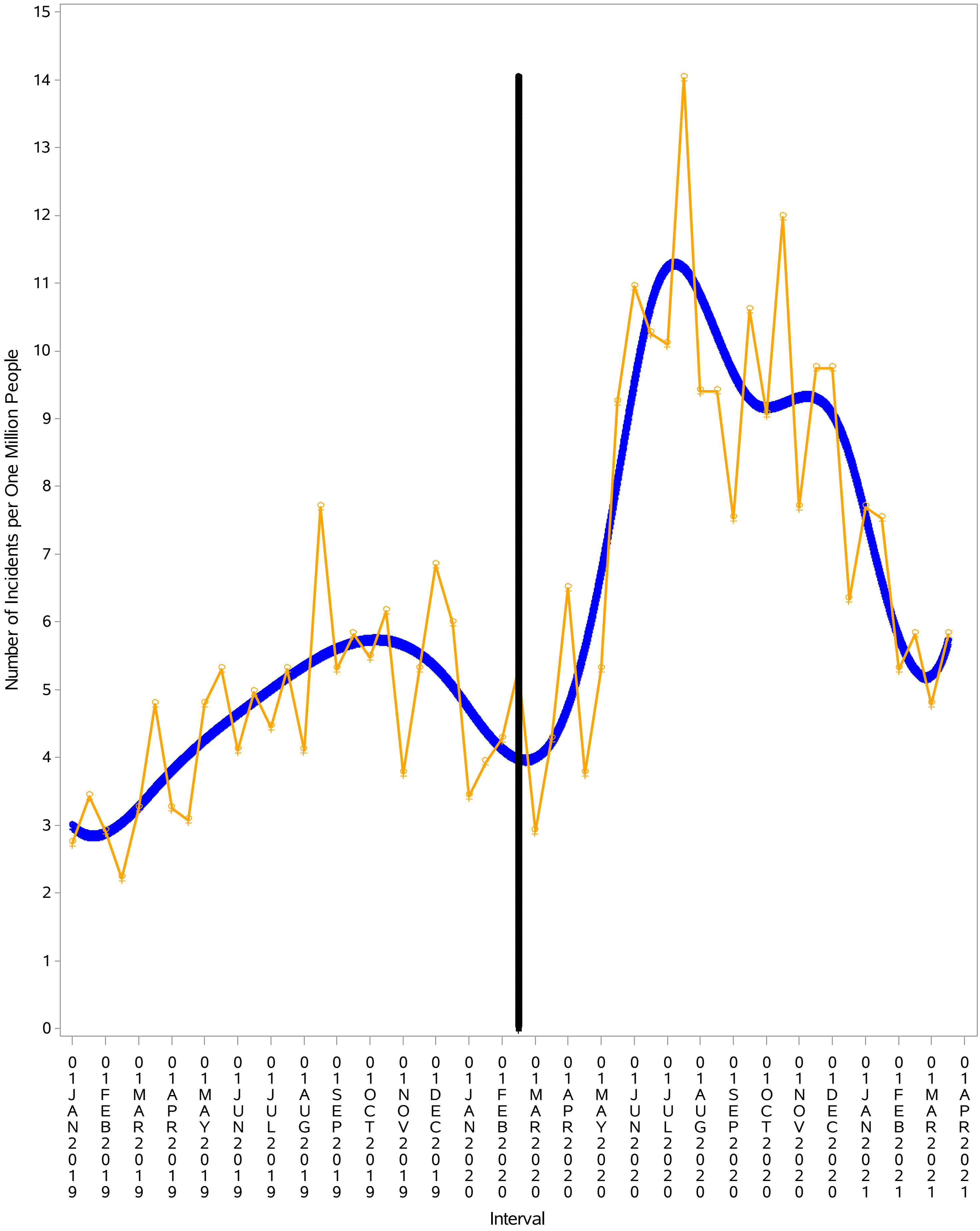

Wisconsin
Bimonthly Data

Comparison	IntensityRatio	IntensityRatio_LowerCL	IntensityRatio_UpperCL	P_Value
**[01MAR2020 thru 15MAR2020] vs [01MAR2019 thru 15MAR2019]**	1.221	0.881	1.692	0.2229
**[16MAR2020 thru 31MAR2020] vs [16MAR2019 thru 31MAR2019]**	1.194	0.868	1.643	0.2686
**[01APR2020 thru 15APR2020] vs [01APR2019 thru 15APR2019]**	1.253	0.910	1.725	0.1629
**[16APR2020 thru 30APR2020] vs [16APR2019 thru 30APR2019]**	1.388	1.031	1.868	0.0313
**[01MAY2020 thru 15MAY2020] vs [01MAY2019 thru 15MAY2019]**	1.586	1.220	2.061	0.001
**[16MAY2020 thru 31MAY2020] vs [16MAY2019 thru 31MAY2019]**	1.821	1.430	2.319	< 0.0001
**[01JUN2020 thru 15JUN2020] vs [01JUN2019 thru 15JUN2019]**	2.052	1.604	2.625	< 0.0001
**[16JUN2020 thru 30JUN2020] vs [16JUN2019 thru 30JUN2019]**	2.213	1.711	2.863	< 0.0001
**[01JUL2020 thru 15JUL2020] vs [01JUL2019 thru 15JUL2019]**	2.245	1.749	2.883	< 0.0001
**[16JUL2020 thru 31JUL2020] vs [16JUL2019 thru 31JUL2019]**	2.166	1.723	2.722	< 0.0001
**[01AUG2020 thru 15AUG2020] vs [01AUG2019 thru 15AUG2019]**	2.023	1.635	2.503	< 0.0001
**[16AUG2020 thru 31AUG2020] vs [16AUG2019 thru 31AUG2019]**	1.864	1.500	2.316	< 0.0001
**[01SEP2020 thru 15SEP2020] vs [01SEP2019 thru 15SEP2019]**	1.724	1.365	2.176	< 0.0001
**[16SEP2020 thru 30SEP2020] vs [16SEP2019 thru 30SEP2019]**	1.631	1.276	2.084	0.0002
**[01OCT2020 thru 15OCT2020] vs [01OCT2019 thru 15OCT2019]**	1.602	1.267	2.026	0.0002
**[16OCT2020 thru 31OCT2020] vs [16OCT2019 thru 31OCT2019]**	1.610	1.294	2.004	0.0001
**[01NOV2020 thru 15NOV2020] vs [01NOV2019 thru 15NOV2019]**	1.645	1.326	2.042	< 0.0001
**[16NOV2020 thru 30NOV2020] vs [16NOV2019 thru 30NOV2019]**	1.683	1.335	2.123	< 0.0001
**[01DEC2020 thru 15DEC2020] vs [01DEC2019 thru 15DEC2019]**	1.702	1.323	2.189	0.0001
**[16DEC2020 thru 31DEC2020] vs [16DEC2019 thru 31DEC2019]**	1.679	1.296	2.174	0.0002
**[01JAN2021 thru 15JAN2021] vs [01JAN2020 thru 15JAN2020]**	1.602	1.241	2.068	0.0006
**[16JAN2021 thru 31JAN2021] vs [16JAN2020 thru 31JAN2020]**	1.499	1.149	1.955	0.0037
**[01FEB2021 thru 15FEB2021] vs [01FEB2020 thru 15FEB2020]**	1.399	1.051	1.862	0.0224
**[16FEB2021 thru 28FEB2021] vs [16FEB2020 thru 29FEB2020]**	1.328	0.993	1.776	0.0558
**[01MAR2020 thru 31MAR2021] vs [01FEB2019 thru 29FEB2020]**	1.663	1.480	1.868	< 0.0001

Wyoming
Bimonthly Data

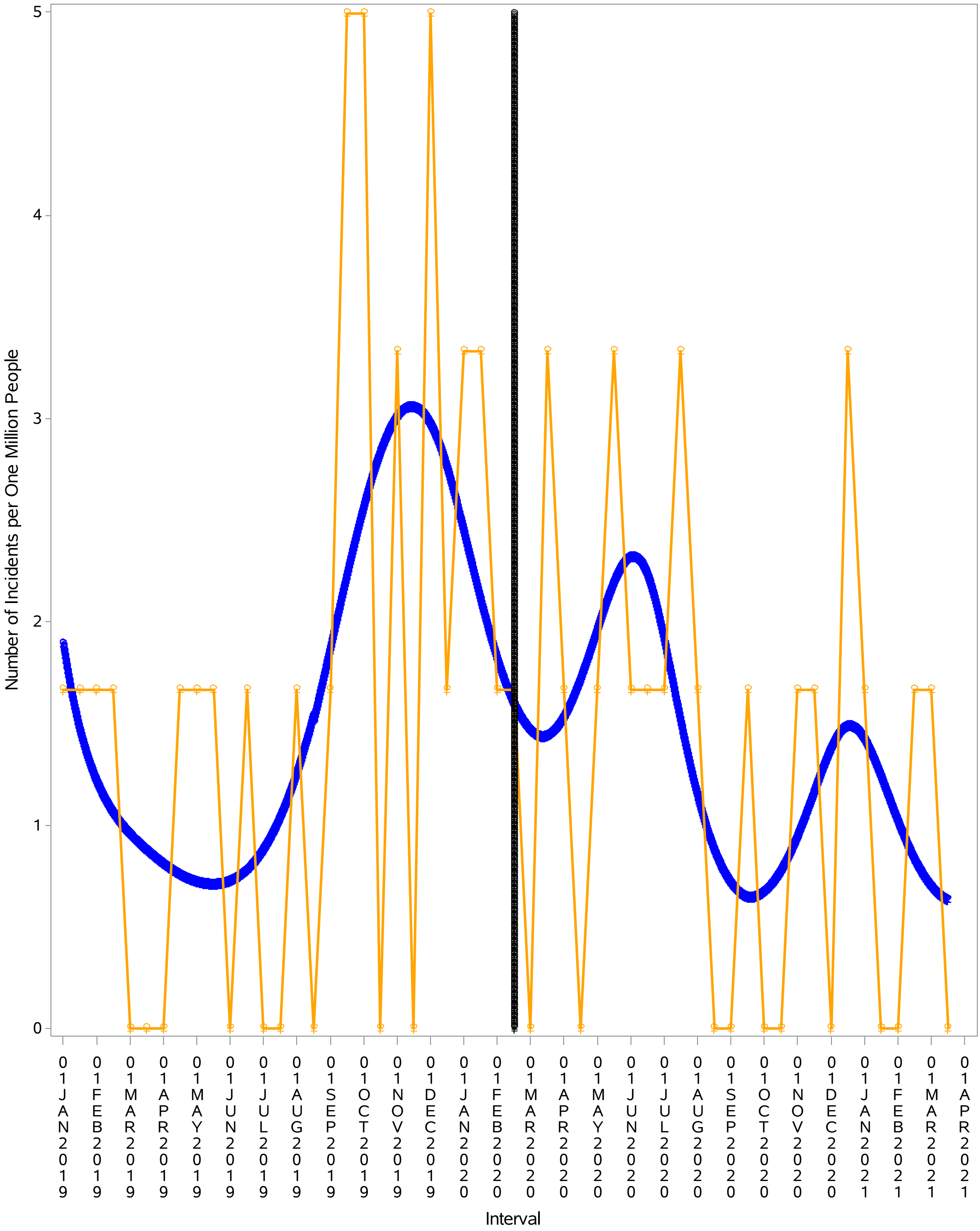

Wyoming
Bimonthly Data

Comparison	IntensityRatio	IntensityRatio_LowerCL	IntensityRatio_UpperCL	P_Value
**[01MAR2020 thru 15MAR2020] vs [01MAR2019 thru 15MAR2019]**	1.535	0.443	5.320	0.4907
**[16MAR2020 thru 31MAR2020] vs [16MAR2019 thru 31MAR2019]**	1.641	0.433	6.224	0.4576
**[01APR2020 thru 15APR2020] vs [01APR2019 thru 15APR2019]**	1.889	0.474	7.528	0.3585
**[16APR2020 thru 30APR2020] vs [16APR2019 thru 30APR2019]**	2.268	0.611	8.424	0.2147
**[01MAY2020 thru 15MAY2020] vs [01MAY2019 thru 15MAY2019]**	2.713	0.817	9.006	0.1007
**[16MAY2020 thru 31MAY2020] vs [16MAY2019 thru 31MAY2019]**	3.087	0.977	9.752	0.0546
**[01JUN2020 thru 15JUN2020] vs [01JUN2019 thru 15JUN2019]**	3.191	0.968	10.517	0.0563
**[16JUN2020 thru 30JUN2020] vs [16JUN2019 thru 30JUN2019]**	2.862	0.824	9.933	0.0956
**[01JUL2020 thru 15JUL2020] vs [01JUL2019 thru 15JUL2019]**	2.166	0.642	7.303	0.2065
**[16JUL2020 thru 31JUL2020] vs [16JUL2019 thru 31JUL2019]**	1.447	0.462	4.529	0.5174
**[01AUG2020 thru 15AUG2020] vs [01AUG2019 thru 15AUG2019]**	0.908	0.300	2.748	0.8615
**[16AUG2020 thru 31AUG2020] vs [16AUG2019 thru 31AUG2019]**	0.571	0.180	1.812	0.3329
**[01SEP2020 thru 15SEP2020] vs [01SEP2019 thru 15SEP2019]**	0.383	0.111	1.321	0.1252
**[16SEP2020 thru 30SEP2020] vs [16SEP2019 thru 30SEP2019]**	0.291	0.082	1.035	0.0563
**[01OCT2020 thru 15OCT2020] vs [01OCT2019 thru 15OCT2019]**	0.265	0.081	0.869	0.0292
**[16OCT2020 thru 31OCT2020] vs [16OCT2019 thru 31OCT2019]**	0.274	0.093	0.808	0.0201
**[01NOV2020 thru 15NOV2020] vs [01NOV2019 thru 15NOV2019]**	0.315	0.113	0.883	0.0289
**[16NOV2020 thru 30NOV2020] vs [16NOV2019 thru 30NOV2019]**	0.381	0.131	1.108	0.0752
**[01DEC2020 thru 15DEC2020] vs [01DEC2019 thru 15DEC2019]**	0.464	0.152	1.413	0.1712
**[16DEC2020 thru 31DEC2020] vs [16DEC2019 thru 31DEC2019]**	0.540	0.181	1.611	0.2618
**[01JAN2021 thru 15JAN2021] vs [01JAN2020 thru 15JAN2020]**	0.583	0.204	1.667	0.3057
**[16JAN2021 thru 31JAN2021] vs [16JAN2020 thru 31JAN2020]**	0.586	0.183	1.873	0.359
**[01FEB2021 thru 15FEB2021] vs [01FEB2020 thru 15FEB2020]**	0.561	0.147	2.142	0.3889
**[16FEB2021 thru 28FEB2021] vs [16FEB2020 thru 29FEB2020]**	0.520	0.127	2.125	0.3541
**[01MAR2020 thru 31MAR2021] vs [01FEB2019 thru 29FEB2020]**	0.844	0.488	1.459	0.5353

USA
Bimonthly Data

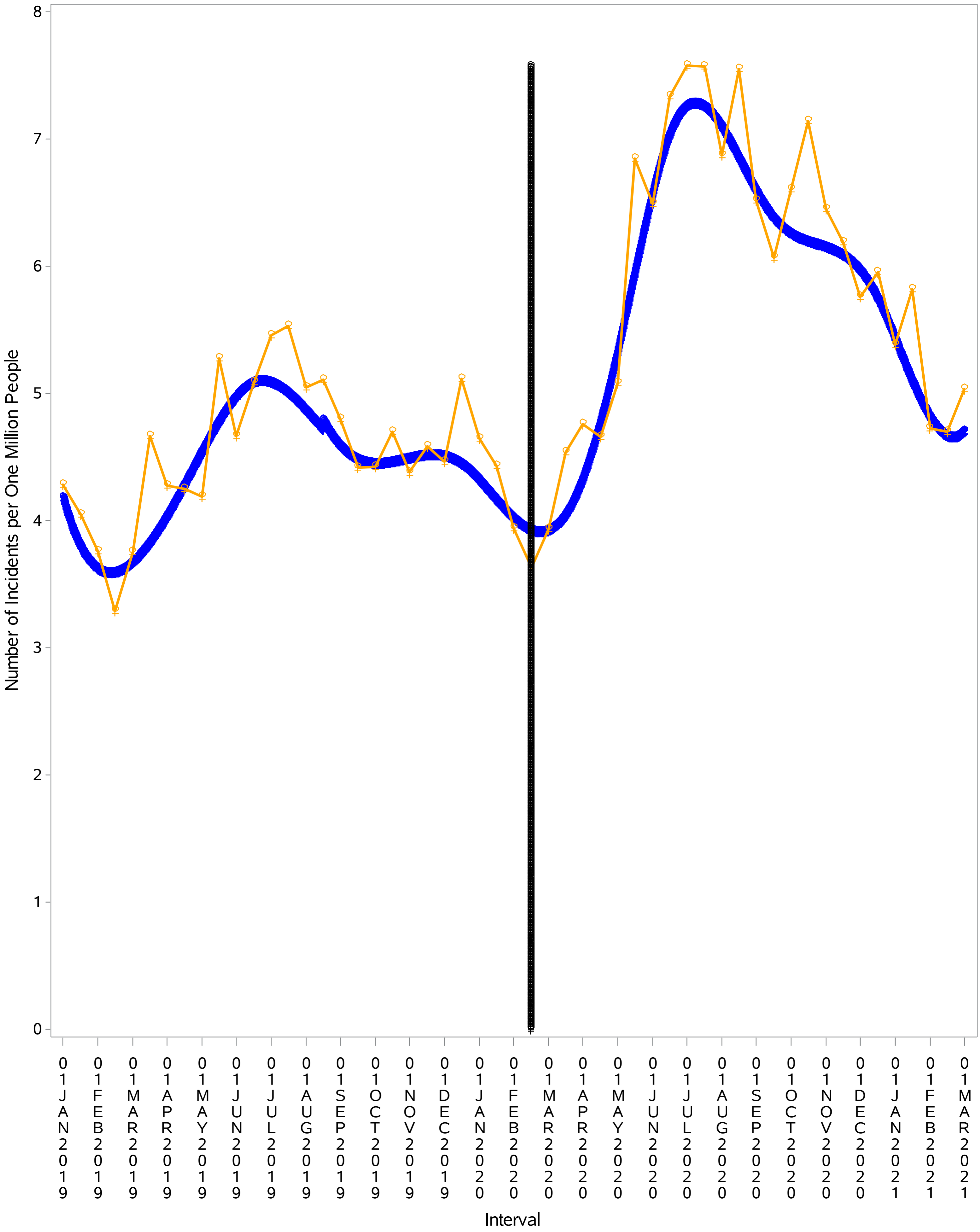

USA
Bimonthly Data

Comparison	IntensityRatio	IntensityRatio_LowerCL	IntensityRatio_UpperCL	P_Value
**Median Age**	0.998	0.939	1.060	0.9451
**Black-White Ratio**	6.575	2.805	15.412	< 0.0001
**Hispanic-White Ratio**	0.846	0.344	2.083	0.7158
**Male-Female Ratio**	0.033	0.000	7.478	0.2173
**[01MAR2020 thru 15MAR2020] vs [01MAR2019 thru 15MAR2019]**	1.069	1.034	1.105	0.0001
**[16MAR2020 thru 31MAR2020] vs [16MAR2019 thru 31MAR2019]**	1.058	1.023	1.093	0.001
**[01APR2020 thru 15APR2020] vs [01APR2019 thru 15APR2019]**	1.074	1.039	1.110	< 0.0001
**[16APR2020 thru 30APR2020] vs [16APR2019 thru 30APR2019]**	1.115	1.081	1.150	< 0.0001
**[01MAY2020 thru 15MAY2020] vs [01MAY2019 thru 15MAY2019]**	1.175	1.143	1.208	< 0.0001
**[16MAY2020 thru 31MAY2020] vs [16MAY2019 thru 31MAY2019]**	1.246	1.213	1.279	< 0.0001
**[01JUN2020 thru 15JUN2020] vs [01JUN2019 thru 15JUN2019]**	1.319	1.283	1.355	< 0.0001
**[16JUN2020 thru 30JUN2020] vs [16JUN2019 thru 30JUN2019]**	1.382	1.343	1.423	< 0.0001
**[01JUL2020 thru 15JUL2020] vs [01JUL2019 thru 15JUL2019]**	1.426	1.387	1.467	< 0.0001
**[16JUL2020 thru 31JUL2020] vs [16JUL2019 thru 31JUL2019]**	1.450	1.414	1.488	< 0.0001
**[01AUG2020 thru 15AUG2020] vs [01AUG2019 thru 15AUG2019]**	1.458	1.422	1.494	< 0.0001
**[16AUG2020 thru 31AUG2020] vs [16AUG2019 thru 31AUG2019]**	1.452	1.416	1.490	< 0.0001
**[01SEP2020 thru 15SEP2020] vs 01SEP2019 thru 15SEP2019]**	1.436	1.397	1.477	< 0.0001
**[16SEP2020 thru 30SEP2020] vs 16SEP2019 thru 30SEP2019]**	1.422	1.381	1.465	< 0.0001
**[01OCT2020 thru 15OCT2020] vs 01OCT2019 thru 15OCT2019]**	1.407	1.368	1.447	< 0.0001
**[16OCT2020 thru 31OCT2020] vs 16OCT2019 thru 31OCT2019]**	1.389	1.353	1.425	< 0.0001
**[01NOV2020 thru 15NOV2020] vs 01NOV2019 thru 15NOV2019]**	1.370	1.335	1.406	< 0.0001
**[16NOV2020 thru 30NOV2020] vs 16NOV2019 thru 30NOV2019]**	1.348	1.311	1.386	< 0.0001
**[01DEC2020 thru 15DEC2020] vs 01DEC2019 thru 15DEC2019]**	1.323	1.284	1.363	< 0.0001
**[16DEC2020 thru 31DEC2020] vs 16DEC2019 thru 31DEC2019]**	1.292	1.254	1.332	< 0.0001
**[01JAN2021 thru 15JAN2021] vs 01JAN2020 thru 15JAN2020]**	1.257	1.221	1.295	< 0.0001
**[16JAN2021 thru 31JAN2021] vs 16JAN2020 thru 31JAN2020]**	1.224	1.188	1.261	< 0.0001
**[01FEB2021 thru 15FEB2021] vs 01FEB2020 thru 15FEB2020]**	1.199	1.161	1.238	< 0.0001
**[16FEB2021 thru 28FEB2021] vs 16FEB2020 thru 29FEB2020]**	1.189	1.150	1.228	< 0.0001
**[01MAR2020 thru 31MAR2021] vs [01FEB2019 thru 29FEB2020]**	1.293	1.276	1.309	< 0.0001
